# Meta-analysis of multi-center transcriptomic profiles and machine learning reveal phospholipase Cβ4 as a Wnt/Ca²^+^ signaling mediator in glioblastoma immunotherapy

**DOI:** 10.3389/fimmu.2025.1610683

**Published:** 2025-08-07

**Authors:** Zhaoming Song, Fei Wang, Chen Yang, Yanao Guo, Jinfeng Li, Run Huang, Hongyi Ling, Guosheng Cheng, Zhouqing Chen, Zhanchi Zhu, Zhong Wang

**Affiliations:** ^1^ Department of Neurosurgery, The First Affiliated Hospital of Soochow University, Suzhou, Jiangsu, China; ^2^ Suzhou Medical College of Soochow University, Suzhou, Jiangsu, China; ^3^ Chinese Academy of Sciences (CAS) Key Laboratory of Nano-Bio Interface, Suzhou Institute of Nano-Tech and Nano-Bionics, Chinese Academy of Sciences, Suzhou, Jiangsu, China

**Keywords:** glioblastoma, PLCB4, machine learning, tumor microenvironment, multi-omics, immunotherapy

## Abstract

**Introduction:**

Glioblastoma (GBM) is a highly aggressive brain tumor characterized by pronounced invasiveness, rapid progression, frequent recurrence, and poor clinical prognosis. Current treatment strategies remain inadequate due to the lack of effective molecular targets, underscoring the urgent need to identify novel therapeutic avenues.

**Methods:**

In this study, we employed weighted gene co-expression network analysis and meta-analysis, incorporating clinical immunotherapy datasets, to identify ten candidate genes associated with GBM initiation, progression, prognosis, and response to immunotherapy. Multi-omics analyses across glioma and pan-cancer datasets revealed that these genes play pivotal roles in cancer biology.

**Results:**

Phospholipase Cb4 (PLCB4) showed a negative correlation with tumor grade in clinical samples, suggesting its potential role as a tumor suppressor. Evidence indicated that PLCB4 expression is modulated by Wnt signaling, and its overexpression may activate the calcium ion signaling pathway. Notably, *PLCB4* is strongly associated with aberrant tumor proliferation, making it a compelling therapeutic target. Through structure-based virtual screening, five small molecules with high predicted affinity for *PLCB4* were identified as potential drug candidates.

**Discussion:**

This study’s integrative approach—combining target identification, pathway inference, and in silico drug screening—offers a promising framework for rational drug development in GBM. The findings may reduce unnecessary experimental screening and medical costs, and represent a significant step toward improving therapeutic outcomes and prognosis for GBM patients.

## 1 Introduction

Glioblastoma (GBM) is the most aggressive and lethal form of glioma, accounting for 70–75% of all diffuse gliomas, with a 5-year survival rate of only 10% (1, 2). Current treatment strategies involve a multimodal approach: maximal safe surgical resection, followed by radiotherapy and chemotherapy with the alkylating agent temozolomide, and often supplemented by tumor-treating fields, which offer modest improvements in prognosis (3, 4). However, GBM’s highly invasive nature and the presence of intratumoral hypoxic regions contribute to the establishment of an immunosuppressive microenvironment. This environment supports the survival of GBM-initiating cells, promoting more aggressive tumor recurrence (5, 6). GBM also severely impairs the p53 signaling pathway, a key tumor suppressor, thereby facilitating malignant progression (7). Concurrently, the tumor activates the phosphoinositide 3-kinase (PI3K) and receptor tyrosine kinase–RAS (RTK–RAS) signaling pathways, resulting in unchecked cell proliferation and suppression of anti-tumor immune responses (8). Therefore, identifying therapeutic targets capable of modulating these pathways is critical for developing effective treatments and improving patient outcomes.

The advent of next-generation sequencing and third-generation sequencing technologies has enabled the establishment of large-scale clinical genomic cohorts, such as The Cancer Genome Atlas (TCGA), the Cancer Cell Line Encyclopedia (CCLE), and the Clinical Proteomic Tumor Analysis Consortium (CPTAC). These resources provide comprehensive multi-omics datasets that have significantly advanced tumor biology research. However, heterogeneity in findings across cohorts—due to differences in sample sources, experimental designs, and data processing—necessitates further investigation to uncover the underlying causes of these discrepancies (9). In this context, multicenter meta-analyses offer a powerful tool to increase statistical robustness, enhance sample size, reduce single-study bias, and improve the reliability of conclusions. Moreover, through subgroup analyses, these approaches can identify heterogeneity across clinical contexts. For example, the ARDS Berlin Definition Study demonstrated the superiority of a revised diagnostic criterion over the traditional standard in predicting mortality via multicenter meta-analysis analysis (10). Such integrative strategies can transform biological and clinical heterogeneity into insights that refine diagnostics and therapeutic decision-making.

Machine learning (ML) has shown substantial promise in patient stratification and treatment response prediction, particularly in glioma research, where it enhances diagnostic accuracy, refines prognostic models, and supports personalized treatment strategies (11, 12). Traditional ML-based predictor screening has primarily relied on the Least Absolute Shrinkage and Selection Operator (LASSO), although each feature selection algorithm uses distinct criteria to identify relevant variables. Employing multiple algorithms can mitigate the randomness and potential bias inherent in single-method approaches (13). For instance, the “GLM with Elastic Net Regularization Classification Learner” algorithm identified integrated molecular and functional signatures of intrinsic apoptotic pathways that best predicted therapeutic vulnerability in glioma (14, 15). The “Classification Abess Learner” has proven effective in selecting statistically robust feature subsets from high-dimensional datasets through adaptive best subset selection (16). Similarly, the “Classification Priority LASSO Learner” employs hierarchical prioritization to identify clinically relevant predictors, thereby improving both the accuracy and interpretability of models applied to complex multi-omics data (17). Finally, the “Classification Tree Learner” has been successfully utilized to analyze human microbiome profiles for distinguishing colorectal cancer from normal tissue (18, 19). Numerous clinical cohorts featuring RNA sequencing (RNA-seq) data and immunotherapy response profiles have been published to date (20). In GBM, responses to anti-PD-1 immunotherapy have been linked to specific molecular alterations, immune infiltration patterns, and immune expression signatures, which collectively reflect the tumor’s clonal evolution during treatment (21). Consequently, extracting immunotherapy response predictors from pan-cancer immunotherapy cohorts using ML-integrated features holds significant translational potential.

In this study, weighted gene co-expression network analysis (WGCNA) was first used to identify gene modules most strongly associated with GBM development and progression. Subsequently, a meta-analysis was conducted to identify genes with stable prognostic relevance and predictive power for immunotherapy response. By intersecting the outcomes of these three layers of screening, we isolated genes that were consistently associated with GBM progression, predictive of immunotherapy efficacy, and prognostically informative. An ML pipeline employing four feature selection algorithms was then implemented to identify 10 key regulators of anti-tumor immunity. These genes were comprehensively evaluated across multiple layers of biological information, including GBM-specific and pan-cancer multi-omics data, immune infiltration characteristics, and immunotherapy outcomes. *In vitro* validation and high-throughput sequencing identified PLCB4 as a promising therapeutic target. Finally, molecular docking and CCK-8 assays were used to screen for candidate compounds targeting PLCB4.

## 2 Methods

### 2.1 Data acquisition

Data were derived from seven independent GBM cohorts. The cohorts were as follows. The Chinese Glioma Genome Atlas (CGGA, http://www.cgga.org.cn/index.jsp) (CGGA325 and CGGA693), The Cancer Genome Atlas Program (TCGA, https://portal.gdc.cancer.gov), The Glioma Longitudinal AnalySiS (GLASS, http://www.synapse.org/glass), Clinical Proteomic Tumor Analysis Consortium (CPTAC, https://pdc.cancer.gov/pdc) and Gene Expression Omnibus (GEO; GSE121720 and GSE147352). The RNA-seq validation cohort comprised 1,817 patients from CGGA, TCGA, and GLASS. An additional 1,151 patients with microarray data were sourced from GEO (Gravendeel and Rembrandt), CGGA301, and ArrayExpress (https://www.ebi.ac.uk/biostudies/arrayexpress; Kamoun). Pan-cancer clinical and multi-omics data were obtained from TCGA (n = 12,106) and LinkedOmicsKB (n = 1042; https://kb.linkedomics.org). Immunotherapy cohorts were accessed via The Tumor Immunotherapy Gene Expression Resource (TIGER, http://tiger.canceromics.org), which included PRJNA482620 (anti-PD-1, GBM), GSE78220 (anti-PD-1, melanoma), GSE91061 (anti-PD-1, melanoma), Braun (anti-PD-1, renal cell carcinoma), PRJEB23709 (anti-PD-1+anti-CTLA-4, melanoma). The RNA-seq data of IMvigor210Core (anti-PD-1, muscle-invasive urothelial carcinoma) were downloaded from the IMvigor210CoreBiologies package.

### 2.2 Data processing

Raw RNA-seq read counts were transformed to transcripts per kilobase million (TPM), followed by log2 and z-score normalization to align gene expression values with microarray-based measurements and enhance inter-sample comparability. Microarray data obtained from GEO were processed using the robust multi-array average (RMA) method implemented in the “Affy” package. To construct a unified meta-cohort, the “ComBat” function from the “sva” package was applied to correct for batch effects caused by non-biological technical variations, using a Bayesian framework. Risk scores for each patient within the meta-cohort were subsequently calculated using the specified formula.


$$Risk\;Score=\sum_{k=1}^{n}\left(Coef*x^{k}\right)$$

### 2.3 Meta-analysis

A meta-analysis was performed using the “meta” package (22). Gene expression values were first log2-transformed and standardized to z-scores across patients to reduce inter-cohort heterogeneity. Risk ratios (RRs) for treatment response and Benjamini-adjusted hazard ratios (HRs) derived from univariate Cox regression—with corresponding 95% confidence intervals (CIs)—were computed using a random-effects model. Statistical significance was set at p< 0.05. To evaluate between-study heterogeneity, chi-square tests and I² statistics were employed, with I² values >50% and significant *p*-values indicating substantial heterogeneity.

### 2.4 Calculation of GBM score and weighted correlation network analysis

Three canonical signaling pathways—p53/cell cycle, PI3K, and RTK-RAS—have been widely implicated in GBM initiation and progression (23). To assess their activity at the individual patient level, single-sample gene set enrichment analysis (ssGSEA) was performed using curated gene sets (24). Co-expression networks were constructed within discovery cohorts using the “WGCNA” package. An optimal soft-thresholding power was selected to approximate scale-free topology. The resulting weighted adjacency matrix was converted into a topological overlap matrix (TOM), and corresponding dissimilarities (1–TOM) were calculated. Distinct gene modules were identified using dynamic tree cutting, and for each gene, both gene significance (GS) and module membership (MM) values were computed.

### 2.5 Machine learning prediction of response to immune checkpoint block therapy

The “mlr3” package, along with its extensions, provided a comprehensive and extensible ML framework for feature selection and classification. Four feature selection algorithms—GLM with Elastic Net Regularization (classif.cv_glmnet), Classification Abess Learner (classif.abess) (16), Priority Lasso Learner (classif.priority_lasso) (17), and Classification Tree Learner (classif.rpart) (19)—were applied to identify key predictors, including the risk score. To assess the predictive power of these features for immune response, the GLM with Elastic Net model was fitted within a nested resampling and hyperparameter tuning workflow, and its performance was evaluated using the area under the curve (AUC). The AUC quantifies the discriminative ability of diagnostic models in medical research, such as in evaluating treatment efficacy or predicting disease risk (25).Genes selected as key features in at least two immunotherapy-treated cohorts (PRJNA482620, GSE91061, Braun, IMvigor, PRJEB23709, PRJEB25780) were classified as regulators of antitumor immune response.

### 2.6 Immune infiltration

To provide a comprehensive immune genomic profile, we analyzed 73 immune-related molecules across seven functional categories: 21 pan-cancer ligands, 19 immune receptors, 14 antigen-presenting molecules, 7 T-cell co-inhibitors, 3 cell adhesion molecules, 3 T-cell co-stimulatory molecules, and 6 additional immunoregulatory components. Spearman correlation analysis was performed to explore their associations. Multiple computational methods were used to quantify immune cell infiltration in the TME. Particularly, the “IOBR” package (26). enabled integration of various deconvolution algorithms—including MCP-counter, xCell, EPIC, CIBERSORT, and quanTIseq—to estimate immune cell abundance. The Immune Cell Abundance Identifier portal was employed to quantify 18 T-cell subtypes and 6 other immune cell types. To investigate the interaction between the ProImmuML signature and immune signaling, we evaluated 15 pre-defined immune regulatory pathways (e.g., T-cell receptor signaling, cytokine–cytokine receptor interactions, T-cell exhaustion (21, 27, 28)) using ssGSEA to derive pathway activity scores, which were then correlated with the ProImmuML signature via Spearman analysis. Additional immune profiling included T-cell dysfunction scores and gene–CTL correlations obtained from the Tumor Immune Dysfunction and Exclusion (TIDE) platform, T-cell subtype abundances from ImmuneCellAI, and analysis of cancer–immunity cycle activity via the Tracking Tumor Immunophenotype portal.

### 2.7 Query genetic necessity in DepMap

The Cancer Dependency Map (DepMap; https://depmap.org/portal/), constructed using experimental techniques such as RNA interference (RNAi) and CRISPR-Cas9, is a comprehensive pan-cancer susceptibility database. It integrates data from cancer cell lines, gene knockout experiments, and offers online analytical tools. DepMap enables queries on gene expression levels, mutational profiles, and gene essentiality across cancer cell lines. A gene was defined as essential if its Chronos Gene Effect Score (29) was significantly lower in mutant cell lines compared to wild-type. Additionally, genes with a correlation between copy number variation (CNV) and Chronos Gene Effect Score< –0.4, or between mRNA expression and Chronos Score< –0.4, were also considered essential.

### 2.8 Transcriptome sequencing

For our RNA sequencing study, glioma tissue samples were obtained from the in-house Gusu dataset. Tissues were pulverized in liquid nitrogen and processed following a protocol approved by the Ethics Committee of the First Affiliated Hospital of Soochow University. Total RNA was extracted using TRIzol reagent, and its quality was assessed using both a NanoPhotometer and the RNA Nano 6000 Assay System to ensure integrity. Library preparation was performed using 1 μg of total RNA per sample, involving mRNA enrichment, cDNA synthesis, end repair, dA-tailing, and adaptor ligation. Libraries were sequenced on Illumina HiSeq, NovaSeq, or MGI2000 platforms using 2×150 bp paired-end (PE) configurations. Gene expression levels were quantified using FPKM values (**Supplementary Table S7**).

### 2.9 Immunohistochemistry

A subset of glioma samples used in RNA sequencing was selected from the Gusu dataset for immunohistochemical (IHC) analysis. Samples were fixed in 4% paraformaldehyde (PFA), embedded in paraffin, and sectioned. Sections were stained with hematoxylin (Cat. No. G1005; Servicebio) and processed for IHC using an anti-PLCB4 antibody (1:1000 dilution; Cat. No. abx131466; Abbexa, Cambridge, UK) in accordance with the manufacturer’s protocol for the IHC detection kit (Cat. No. PK10006; Proteintech).

### 2.10 Cell culture

U87 glioma cells were cultured in Dulbecco’s modified Eagle medium (DMEM; Gibco, USA) supplemented with 10% fetal bovine serum (FBS; Gibco, USA) and 1% penicillin-streptomycin (Servicebio, China). Cells were maintained at 37°C in a humidified incubator with 5% CO_2_ and subcultured at 80–90% confluence using 0.25% trypsin-EDTA (Servicebio, China). Cell viability and morphology were monitored via phase-contrast microscopy. Cells from passages 5–15 were used in all experiments to ensure genetic consistency.

### 2.11 Lentiviral transduction for PLCB4 overexpression

The human PLCB4 coding sequence (Gene ID: 5332) was cloned into a lentiviral overexpression vector driven by a CMV promoter. A scrambled, non-targeting sequence served as the negative control (NC). Lentiviral particles were generated in 293T cells using a third-generation packaging system and transfected with Lipofectamine 3000 (Invitrogen, USA). Viral supernatants were collected at 48 h and 72 h post-transfection, filtered through a 0.45 μm PVDF membrane, and concentrated using Lenti-X™ Concentrator. U87 cells were seeded into 6-well plates at 5 × 10^4^ cells/well and transduced at 50–60% confluence with lentiviral particles at a multiplicity of infection (MOI) of 10 in the presence of 8 μg/mL polybrene. After 24 h, the medium was replaced with fresh complete DMEM, and stable transductants were selected using 2 μg/mL puromycin (Beyotime, China) for 7 days.

### 2.12 Quantitative real-time PCR

Quantitative real-time PCR (qRT-PCR) was performed to assess the expression of APCDD1, RAC2, and PLCB4. Total RNA was isolated from U87 cells using TRIzol^®^ reagent (Invitrogen, USA), and its concentration and purity were determined with a NanoDrop spectrophotometer (Thermo Fisher Scientific, USA). First-strand cDNA synthesis was carried out using 1 μg RNA and the PrimeScript™ RT Reagent Kit (ACE, China). qPCR was performed using SYBR Green Premix (Vazyme, China) on an Applied Biosystems system. Primers for target and reference genes were designed using Primer-BLAST and validated for specificity. The thermal cycling conditions were: initial denaturation at 95°C for 30 s, followed by 40 cycles of 95°C for 5 s and 60°C for 30 s. Melting curve analysis confirmed the specificity of amplification. Relative gene expression was calculated using the 2^−ΔΔCT^ method. Primer sequences are provided in **Supplementary Table S8**.

### 2.13 EdU staining assay

Cell proliferation was evaluated using the Click-iT™ EdU Imaging Kit (Beyotime, China). Cells were incubated with 10 μM EdU for 2 h, fixed in 4% paraformaldehyde, permeabilized with 0.5% Triton X-100, and stained with Alexa Fluor^®^ 594-conjugated EdU detection reagent. Nuclei were counterstained with Hoechst 33342 (5 μg/mL). Fluorescence microscopy was used for imaging, and EdU-positive cells were quantified using ImageJ software.

### 2.14 Cell proliferation assay

For viability assays, cells were seeded in 96-well plates at 3000 cells/well and incubated for 24 h. Following treatment, 10 μL of CCK-8 reagent (Beyotime, China) was added to each well and incubated at 37°C for 4 h. Absorbance was measured at 450 nm using a microplate reader. Cell viability was expressed relative to the untreated control group.

### 2.15 Drug intervention

We employed quantitative real-time polymerase chain reaction (qPCR) to validate the regulatory effects of modulators of the three Wnt signaling pathways on the gene expression levels of PLCB4, APCDD1, and RAC. Specifically, SKL-2001 and MSAB (TargetMol, China) were used as the activator and inhibitor of the canonical Wnt/β-catenin pathway, respectively. Lonomycin (LON; TargetMol, China) and 2-APB (TargetMol, China) served as the activator and inhibitor of the Wnt/Ca²^+^ pathway, while Wnt5a (TargetMol, China) and blebbistatin (TargetMol, China) were used to modulate the Wnt/PCP pathway. When cell confluency reached approximately 80% (assessed via automated cell counter) or the cell concentration was between 2.8 × 10^6^ and 3.2 × 10^6^ (measured using a cell counting plate), the culture medium was removed, cells were washed with phosphate-buffered saline, and fresh medium containing the corresponding compounds was added. Drug concentrations were selected based on manufacturer recommendations and previous studies, resulting in final concentrations of 20 μM for the skl2001 group (30), 20 μM for the MSAB group (31), 100 μM for the 2-APB group (32), 5 μM for the lonomycin group, 20 μM for the blebbistatin group (33), and 20 μM for the Wnt5a group, among others. Following 24 hours of incubation, total RNA was extracted and analyzed using qPCR.

### 2.16 RNA sequencing and downstream analysis

Total RNA was isolated using TRIzol reagent (Invitrogen) according to the manufacturer’s protocol. RNA purity and concentration were assessed with a NanoDrop 2000 spectrophotometer (Thermo Scientific), and RNA integrity was evaluated using the Agilent 2100 Bioanalyzer (Agilent Technologies). RNA-seq libraries were constructed using the VAHTS Universal V6 RNA-seq Library Prep Kit and sequenced on an Illumina NovaSeq 6000 platform, generating 150 bp paired-end reads. Raw FASTQ reads were aligned to the reference genome using STAR (34).

Differential gene expression analysis was performed using DESeq2. Significantly differentially expressed genes (DEGs) were defined by a false discovery rate (FDR)-adjusted *p*-value< 0.05, *Q*-value< 0.05, and fold change > 2 or< 0.5. Functional enrichment analyses, including Kyoto Encyclopedia of Genes and Genomes (KEGG) and Gene Set Enrichment Analysis (GSEA) of HALLMARK datasets, were conducted using the “clusterProfiler” R package (35).

### 2.17 Molecular docking–based virtual screening targeting PLCB4

To identify potential small molecules targeting *PLCB4*, virtual screening based on molecular docking was performed. The active sites H328 and H375 were predicted using the Site Finder module in MOE, and Site 1 was selected for screening. The Protein Preparation Wizard in Schrödinger was used to optimize the Alphafold-predicted structure of *PLCB4*, including bond order correction and protonation (pH 7.0) using the PROPKA method. Structural energy minimization was carried out using the OPLS4 force field, with a convergence criterion of 0.3 Å for the RMSD of heavy atoms. The T001 compound library (TopScience Co., Ltd.) was processed using LigPrep (Schrödinger, LLC, New York, NY, 2021). Candidate molecules were evaluated through molecular docking, protein-ligand interaction fingerprint (PLIF) analysis, clustering, and binding mode analysis. Compounds with the highest binding affinities for PLCB4 were shortlisted as potential therapeutic agents.

### 2.18 Statistical analysis

All data processing, statistical analyses, and visualizations were performed in R version 4.3.3. Group comparisons of continuous variables were conducted using the Wilcoxon rank-sum test. Survival analysis was carried out using Kaplan–Meier (K-M) curves and log-rank tests. Spearman correlation analysis was employed to evaluate associations between continuous variables. Both univariate and multivariate Cox regression analyses were performed using the “survival” R package. The single-sample GSEA (ssGSEA) algorithm implemented in the “GSVA” package was used to estimate pathway activity based on curated gene sets. A p-value< 0.05 was considered statistically significant.

## 3 Results

### 3.1 Identifying ProImmuML signature as key regulators in GBM predicting immunotherapy response and prognosis through meta-analysis and machine learning

**Figure 1:**
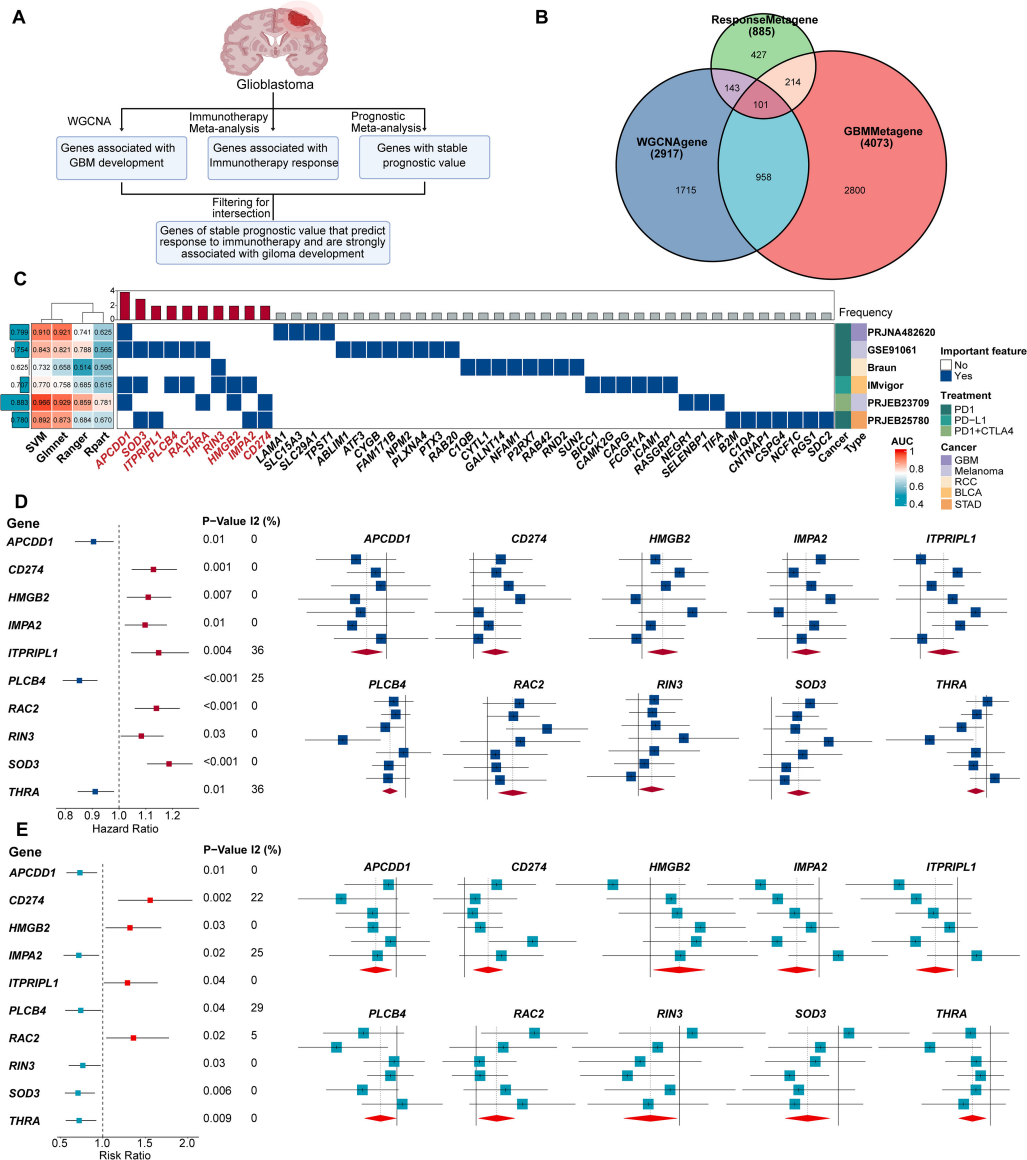
10 genes of stable prognostic value, predicting response to immunotherapy and strongly associated with GBM development were screened through ProImmuML. **(A)** Screening of key genes for GBM through WGCNA, immunotherapy meta-analysis and prognostic meta-analysis. **(B)** Venn diagram showing the interaction of the three sets of screening results. **(C)** Identification of important features by 4 machine learning algorithms for feature selection in 6 cancer immunotherapy cohorts. **(D)** Prognostic meta-analysis of the ProImmuML signature. **(E)** Immune response meta-analysis of the ProImmuML signature.

To identify genes predictive of immunotherapy response and with consistent prognostic value in GBM, we designed a comprehensive analysis pipeline (**Figure 1A**). We incorporated data from seven GBM cohorts and evaluated pathway activities across p53, PI3K, and RTK signaling axes, which exhibited substantial inter-patient variability (**Supplementary Figure S1A**). WGCNA identified 12 gene modules (**Supplementary Figure S1B**), with the blue, brown, and green modules positively associated with GBM progression, while the turquoise module showed a strong negative association (**Supplementary Figure S1C**).

The initial WGCNA screening yielded 2,917 genes associated with GBM development across all seven cohorts (**Figure 1B**, **Supplementary Table S2**). Concurrently, a meta-analysis of immunotherapy response identified 886 genes (**Supplementary Table S3**), and a separate prognostic meta-analysis identified 4,073 genes with stable prognostic value (**Supplementary Table S4**). Intersection of these three gene sets resulted in 101 candidate genes that met all criteria—associated with GBM development, predictive of immunotherapy response, and possessing stable prognostic value (**Figure 1B**, **Supplementary Table S5**). Functional enrichment using Metascape revealed that these genes were largely involved in anti-tumor immunity (**Supplementary Figure S2**). We then employed multiple ML algorithms across six immunotherapy cohorts to identify the most predictive features, ultimately defining a ten-gene signature termed ProImmuML (Prognostic and Immunotherapy Meta-analysis and Machine Learning). This signature includes APCDD1, SOD3, ITPRIPL1, PLCB4, RAC2, THRA, RIN3, HMGB2, IMPA2, and CD274 (**Figure 1C**). Prognostic meta-analysis identified PLCB4, APCDD1, IMPA2, RIN3, SOD3, and THRA as protective factors, whereas CD274, HMGB2, ITPRIPL1, and RAC2 were classified as risk factors (**Figure 1D**). Notably, higher expression of PLCB4, APCDD1, and THRA correlated with reduced responsiveness to immunotherapy (**Figure 1E**).

### 3.2 Exploring the characteristics of the ProImmuML signature at a multi-omics level in GBM

**Figure 2:**
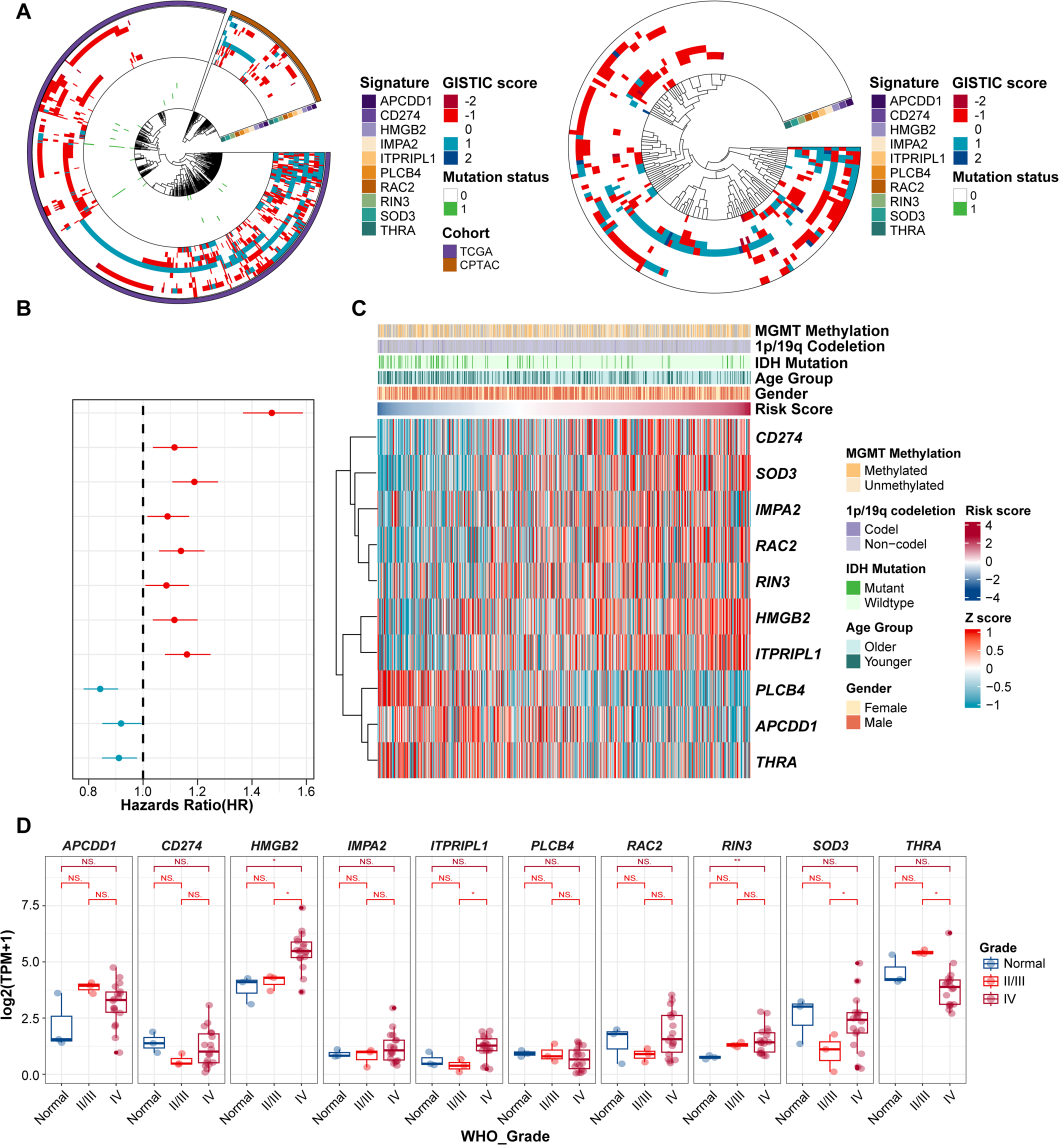
Evaluation of ProImmuML signature in GBM with Multi-omics landscape. **(A)** CNVs and SNVs of ProImmuML signature in genomic data of TCGA cohort and CPTAC cohort (left panel), and GLASS cohort (right panel). **(B)** Prognostic value of the ProImmuML signature by univariate cox regression analysis. **(C)** Relationship between clinical characteristics and the increasing order of risk score. **(D)** The expression of ProImmuML signature in the Gusu in-house dataset. Significant difference, *P<0.05, **P<0.01. NS, Not Significant.

We next investigated the potential of ProImmuML signature genes as therapeutic targets for GBM from a multi-omics perspective. At the genomic level, CNV deletion of PLCB4 was observed in more than half of the patients across the TCGA, CPTAC, and GLASS cohorts, potentially explaining its downregulation in GBM. Notably, the high mutation frequency of PLCB4 further supported its potential role as a tumor suppressor gene (36) (**Figure 2A**). To assess the transcriptomic relevance of the ProImmuML signature, we computed individual patient risk scores based on gene expression and evaluated their association with clinical characteristics. Univariate Cox regression analysis aligned with results from our prognostic meta-analysis (**Figure 2B**). Stratifying patients by increasing risk scores revealed significant correlations with clinical variables such as MGMT methylation, 1p/19q codeletion, IDH mutation status, age, and sex (**Figure 2C**). In our in-house cohort (**Supplementary Table S6**), the expression levels of CD274, HMGB2, RAC2, RIN3, and SOD3 were elevated in WHO grade IV gliomas compared to grades II/III. In contrast, APCDD1, THRA, and PLCB4 were downregulated, while ITPRIPL1 exhibited an increasing trend (**Figure 2D**). We validated these findings through a meta-analysis that integrated RNA-seq (n = 1,817) and microarray (n = 1,151) datasets from LGG and GBM samples, confirming that low PLCB4 expression was associated with poor overall survival (HR = 1.32, 95% CI: 1.11–1.57, p< 0.001) (**Supplementary Figure S3**). This association was corroborated in our internal cohort, where PLCB4 expression significantly decreased with increasing tumor grade (WHO IV vs. II/III: p< 0.01). However, the limited sample size of this cohort introduces certain limitations to the findings.

In the CPTAC dataset, PLCB4 transcript levels were elevated in tumors harboring ATRX, TP53, and IDH mutations, and downregulated in PTEN-mutant tumors (**Supplementary Figure S4A**). Since proteins execute gene functions, we extended our transcriptomic analysis to the proteomic level. Correlation analyses demonstrated strong concordance between mRNA and protein levels for RIN3 (R = 0.714, p< 0.05), CD274 (R = 0.763, p< 0.05), and RAC2 (R = 0.818, p< 0.05), while PLCB4 showed only a moderate positive correlation (R = 0.348, *p*< 0.05; **Supplementary Figure S4B**).

### 3.3 ProImmuML Signature was a promising therapeutic target for pan-cancer

**Figure 3:**
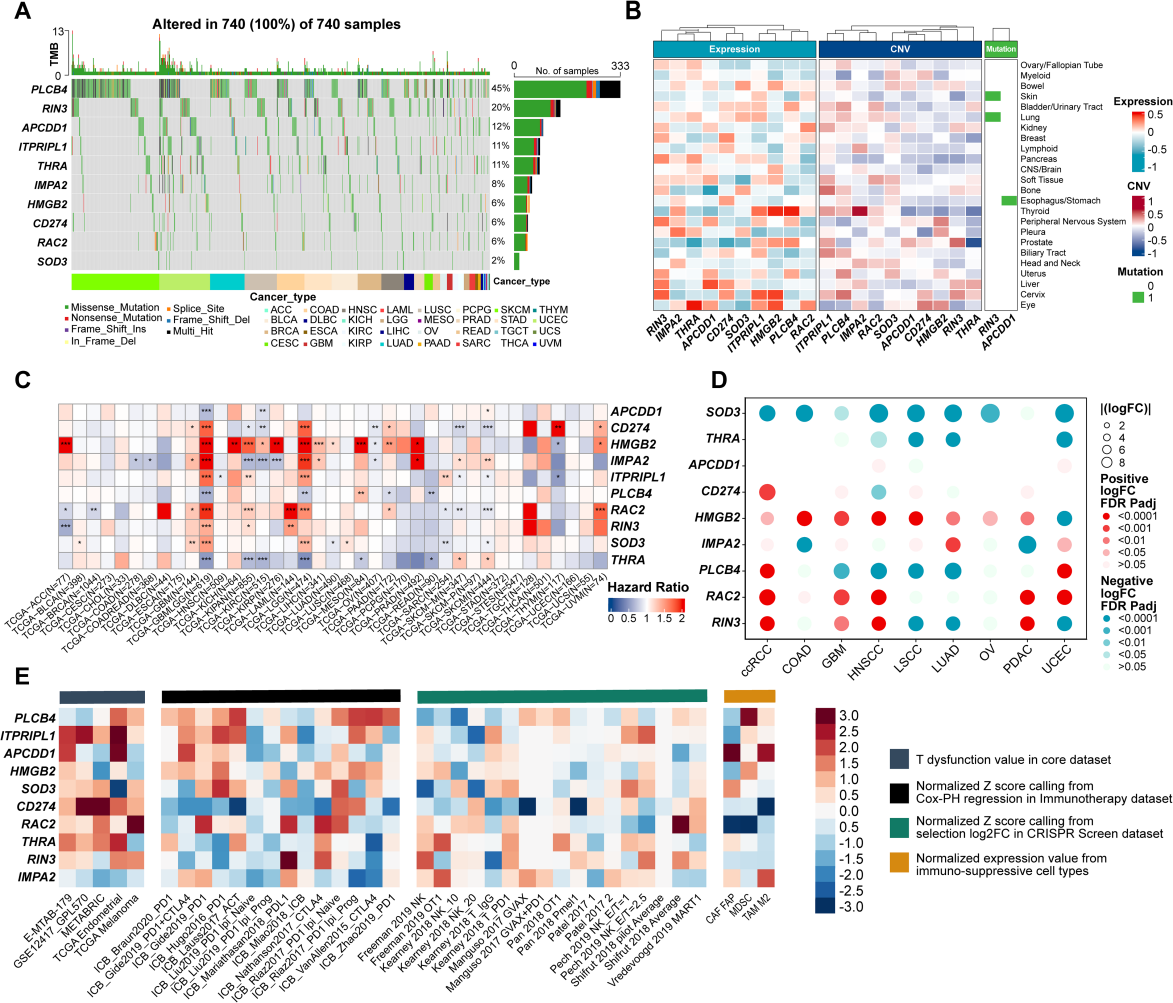
Evaluation of ProImmuML signature at the multi-omics level in pan-cancer. **(A)** Count of deleterious mutations (Missense_Mutaton, Nonsense_Mutation, Frame_Shift_Ins, Splice_Site, Frame_Shift_Del, In_Frame_Del, Multi_Hit) of the ProImmuML signature in 29 cancer types. **(B)** Correlation between mRNA expression (left panel), CNVs (middle panel) and mutation status (right panel) of the ProImmuML signature with the CRISPR effect in each cancer cell line in DepMap database. **(C)** The heatmap showed the hazard ratio of the ProImmuML signature including 36 cancer types from TCGA. **(D)** Bubble plot showing the result of differential analysis of the protein expression of the ProImmuML signature including 9 different cancer types from CPTAC database and GTEx database. **(E)** The heatmap from the “Gene set prioritization” module of the TIDE portal identified the role of the ProImmuML signature in resistance to ICB. Genes (row) were ranked by their weighted average value across four immunosuppressive indices (columns), including T cell dysfunction score, association with ICB survival outcome, log-fold change (logFC) in CRISPR screens, and T cell exclusion score. Significant difference, *P<0.05, **P<0.01, ***P<0.001.

A waterfall plot of genomic alterations revealed that 740 samples had at least one single nucleotide variant (SNV), with PLCB4 mutated in 45% of patients—compared to only 2% for SOD3. All ten ProImmuML genes exhibited mutations, predominantly missense, reinforcing their relevance in GBM pathogenesis. These genomic alterations suggest that PLCB4 may function upstream in tumor regulatory pathways (**Figure 3A**). We also examined correlations between gene CNVs, mRNA expression, and cancer cell lethality following CRISPR knockout. Notably, IMPA2, SOD3, CD274, ITPRIPL1, and RAC2 displayed relatively negative correlations across multiple cancers, including glioma (**Figure 3B**). Analysis of gene expression differences across 34 cancer types versus adjacent normal tissues showed significant dysregulation in all key genes (**Supplementary Figure S5**). Transcriptomic data from 36 tumor types identified PLCB4 as a protective factor in more than 10 cancers (**Figure 3C**). Protein-level analyses in nine cancer types revealed decreased expression of PLCB4, SOD3, and THRA, alongside increased expression of HMGB2 and RAC2, relative to normal tissues (**Figure 3D**). Consistent with findings by Waugh, PLCB4 was significantly downregulated in various cancers, including glioma (36). To further explore its role in immunotherapy, we used the “Gene Set Prioritization” module to assess relevance to immune checkpoint blockade (ICB) resistance. The results indicated that PLCB4 may contribute to immunotherapy response modulation (**Figure 3E**).

### 3.4 Analysis of immune cell infiltration status reveals ProImmuML Signature was a key regulator in the anti-tumor immunity

**Figure 4:**
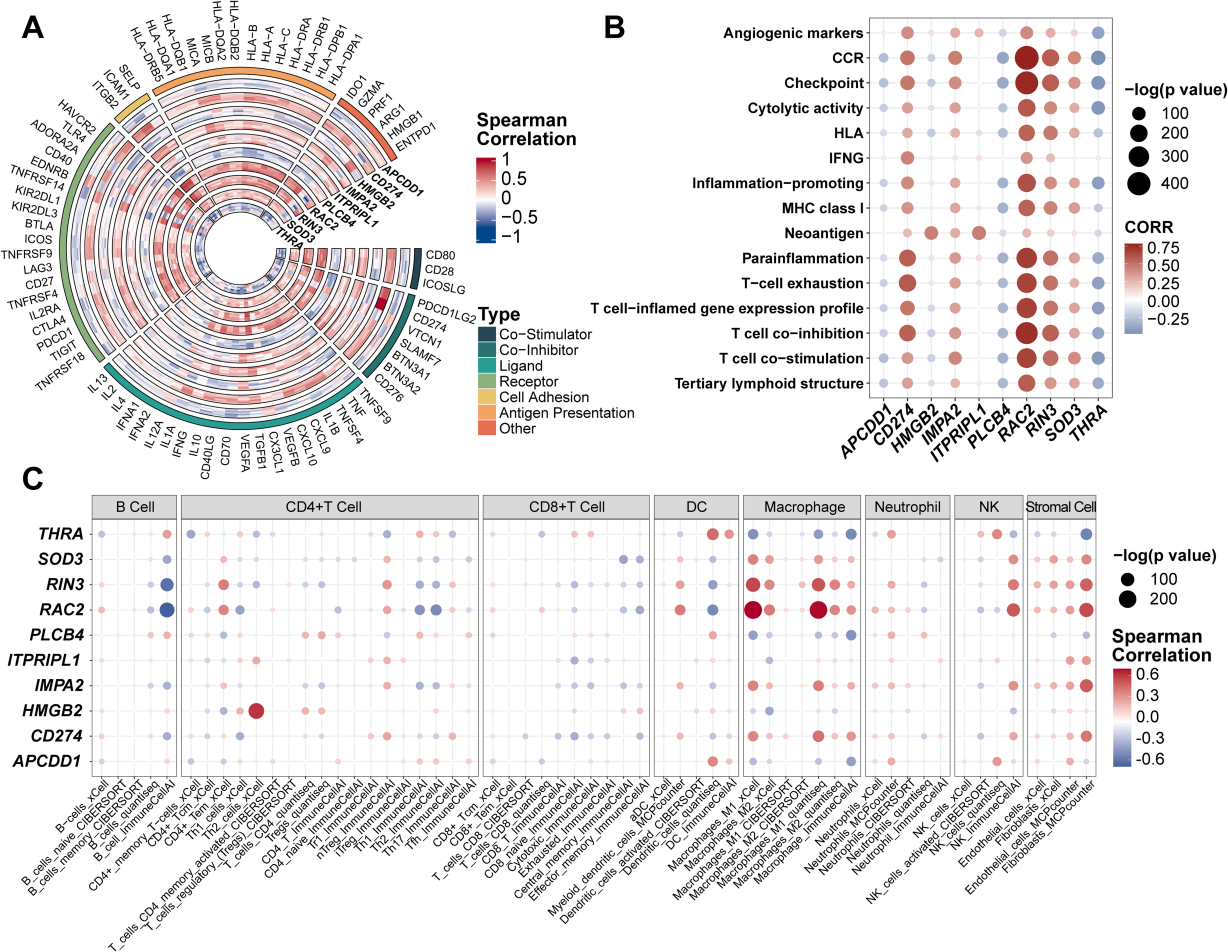
Evaluation of ProImmuML signature in immune infiltration. **(A)** Correlation analyses between the ProImmuML signature and the expression of 74 key immune modulators in 7 GBM cohorts. **(B)** The bubble plot showed the correlation between the ProImmuML signature and 15 gene sets of immune signaling pathways collected from previously published literature. **(C)** Bubble plot showing the correlation between the ProImmuML signature and the infiltration level of different immune cells with five robust deconvolution algorithms.

The tumor microenvironment (TME), comprising both internal and external cellular contexts, plays a crucial role in tumor initiation, progression, and metastasis. Immune correlation analyses revealed that CD274, IMPA2, ITPRIPL1, RAC2, RIN3, and SOD3 were positively associated with immune regulation, whereas PLCB4, THRA, HMGB2, and APCDD1 were negatively correlated (**Figure 4A**). Using 15 predefined immune pathway gene sets, we computed ssGSEA scores across individual patients (**Figure 4B**). CD274, RAC2, and RIN3 showed the strongest positive associations with immune pathway activation, while PLCB4 and THRA were negatively associated. Other genes—including APCDD1, HMGB2, IMPA2, ITPRIPL1, and SOD3—showed no significant correlations, consistent with earlier ProImmuML signature–immune association findings (**Figure 4A**). To further investigate the relationship between the ProImmuML signature and immune infiltration, we applied multiple deconvolution algorithms. The signature correlated most strongly with infiltration of macrophages, natural killer (NK) cells, and stromal cells, with RIN3 and RAC2 exhibiting particularly robust associations. In contrast, correlations with B cells and CD8^+^ T cells were either weak or negative (**Figure 4C**).

### 3.5 ProImmuML Signature Score exhibits excellent predictive ability for immunotherapy response

**Figure 5:**
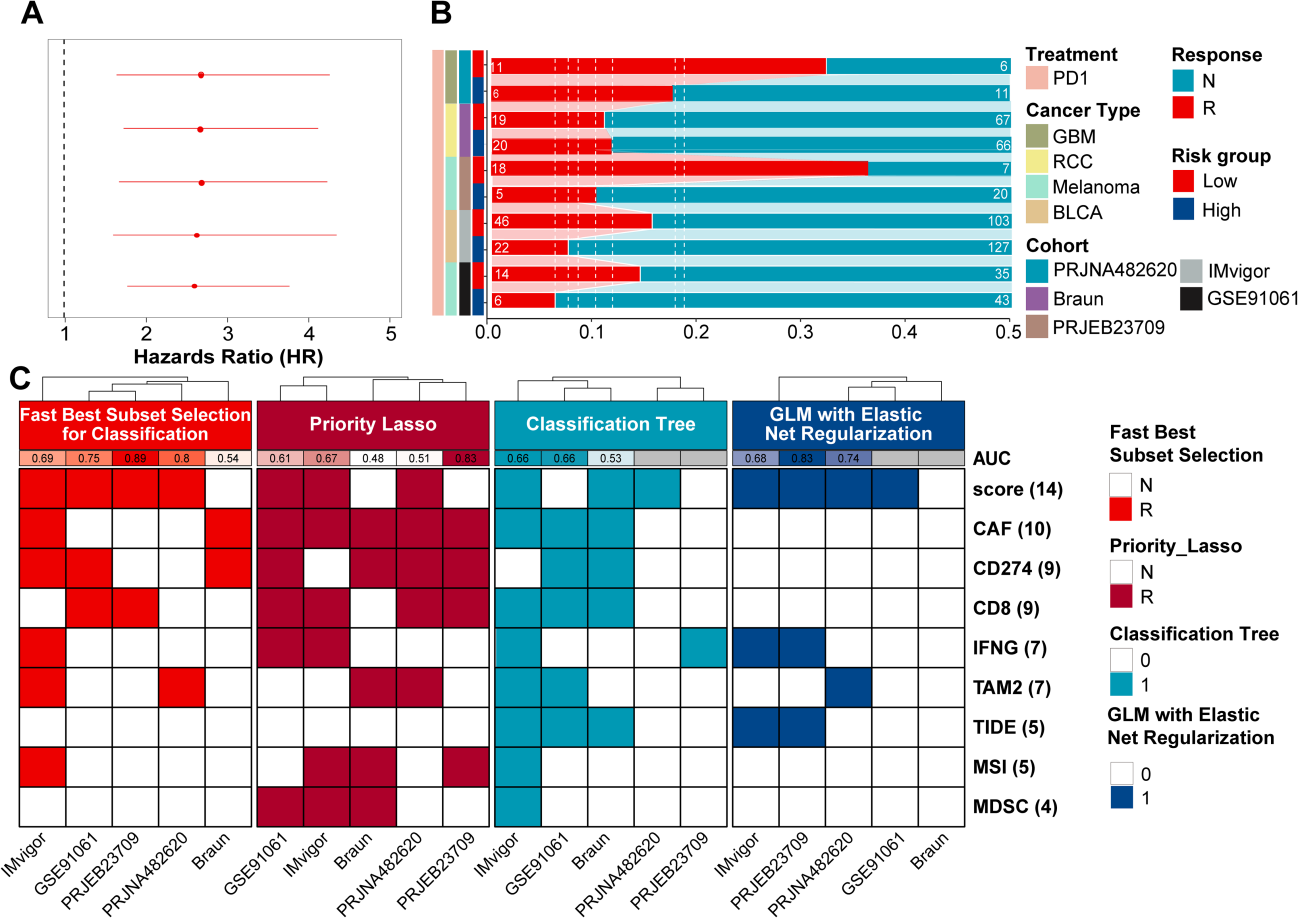
Evaluation of ProImmuML signature in predicting pan-cancer immunotherapy response. **(A)** Univariate cox regression analysis result of immunotherapy response in pan-cancer. **(B)** Stacked bar chart showing the proportion of patients from five cohorts with response to PD-1immunotherapy in high-risk score group and low-risk score group. **(C)** In the machine learning classification task, four feature selection algorithms (“Classification Bayesian Additive Regression Trees Learner”, “Classification Priority Lasso Learner”, “Classification Tree Learner”, “GLM with Elastic Net Regularization Classification Learner”) were used for the five immunotherapy response cohorts. The model was fit using the GLM with Elastic Net Regularization Classification Learner algorithm.

Based on prior analyses, the ProImmuML signature demonstrated potential as a regulator of immunotherapy response (**Figure 5A**). Using this signature, we calculated risk scores across five immunotherapy cohorts and stratified patients into high-risk and low-risk groups. Except for the RCC cohort, the low-risk group consistently exhibited a higher proportion of responders, with particularly marked differences observed in the GBM and melanoma cohorts (**Figure 5B**). To assess predictive performance, we compared the ProImmuML-derived risk score with established predictors from the TIDE algorithm (CAF, CD274, CD8, IFNG, TAM2, TIDE, MSI, MDSC). Feature importance was evaluated using four ML algorithms, and predictive performance was quantified via AUC values. Notably, the GBM cohort exhibited the highest AUC values across models, especially under the Best Subset Selection for Classification (Abess) algorithm (AUC = 0.89). Among all variables, the risk score emerged as the most predictive feature, further supporting its utility in forecasting immunotherapy response (**Figure 5C**, **Supplementary Figure S6**).

To enhance predictive accuracy, we developed a composite model incorporating the five most informative features: risk score, CAF, IFNG, CD274, and CD8. ML analysis across the five immunotherapy cohorts demonstrated that this integrated model outperformed individual predictors. Moreover, predictive accuracy improved as more features were included (**Supplementary Figure S7**).

### 3.6 Overexpression of PLCB4 activated Wnt/Ca2+ signaling pathway

**Figure 6:**
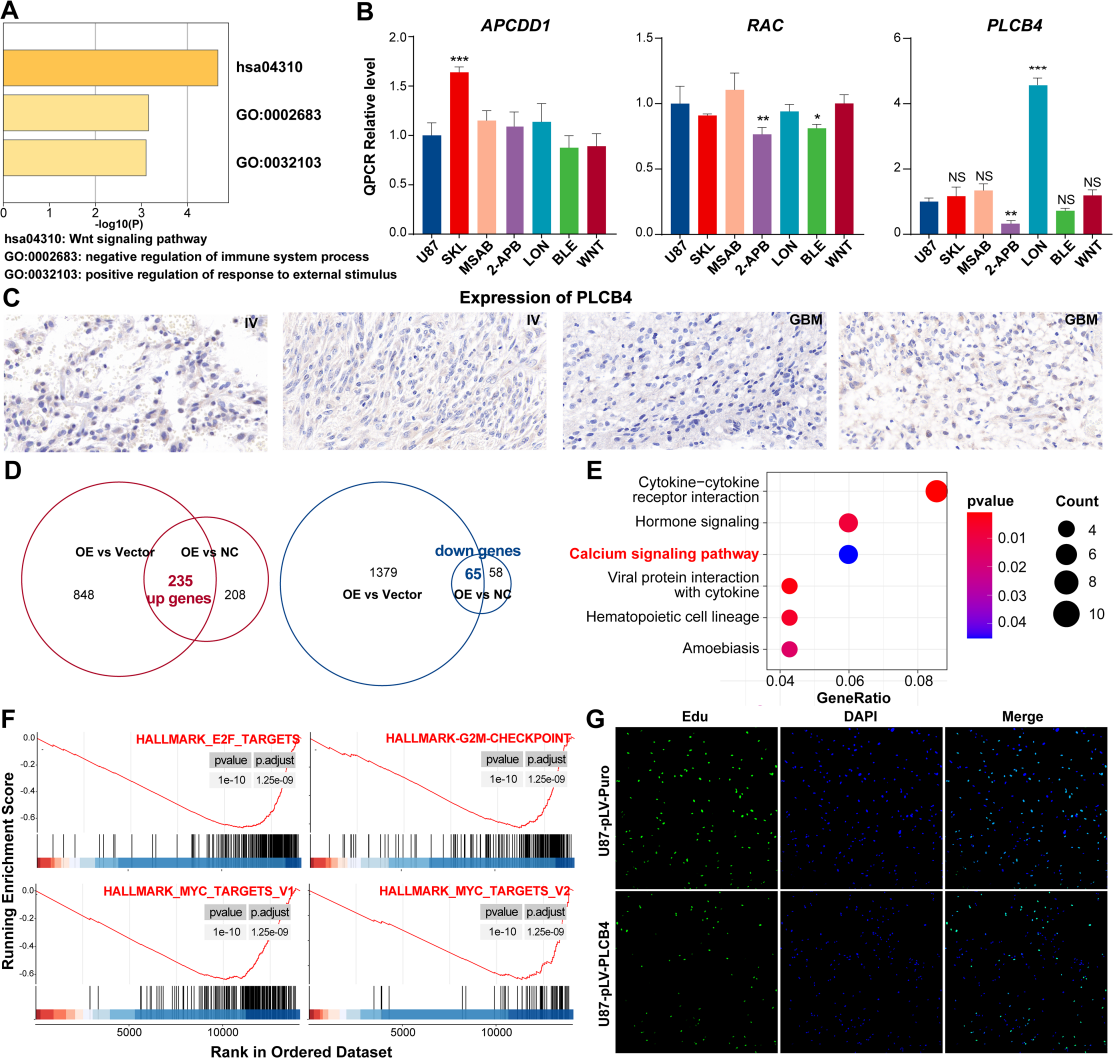
*In vitro* experiments showing that PLCB4 in the ProImmuML signature may be a downstream regulator of the Wnt pathway and inhibit GBM proliferation. **(A)** Metascape analysis of the ProImmuML signature. **(B)** qPCR analysis was performed to analyze the effects of three different Wnt pathway agonists/inhibitors on the expression of APCDD1 (left panel), RAC2 (middle panel), and PLCB4 (right panel). SKL: SKL-2001, Wnt/β-catenin signaling pathway agonist; MSAB: MSAB, Wnt/β-catenin signaling pathway inhibitor; 2-APB: 2-APB, Wnt/Ca^2+^ signaling pathway inhibitor; LON: Lonomycin, Wnt/Ca^2+^ signaling pathway agonist; BLE: Blebbistatin, Planar cell polarity pathway inhibitor; WNT: Wnt5a, Planar cell polarity pathway agonist. **(C)** Representative IHC staining images of PLCB4 from four glioma patients with higher expression of PLCB4 (left panel, n = 2) and lower expression of PLCB4 (right panel, n = 2). **(D)** The intersection of genes from two parallel comparisons identified 235 upregulated genes (left panel) and 65 downregulated genes (right panel). **(E)** KEGG enrichment analysis of the upregulated genes. **(F)** GSEA revealing proliferation-related pathways were significantly inhibited after overexpressing PLCB4. **(G)** EdU assay showing PLCB4 inhibiting the proliferation of glioma cells in U87. Significant difference, *P<0.05, **P<0.01, ***P<0.001.

Metascape-based pathway enrichment analysis of the ProImmuML signature revealed that ten key genes were significantly enriched in the Wnt signaling pathway (**Figure 6A**). Among them, APCDD1, RAC2, and PLCB4 were distributed across three distinct Wnt sub-pathways (**Supplementary Figure S8**). It is important to note that KEGG pathway diagrams aggregate information from diverse biological contexts, which may not directly reflect glioma-specific mechanisms. Although a direct role for PLCB4 in the Wnt pathway within glioma has not previously been reported, our analysis suggests a functional connection involving these three genes. To investigate this, we conducted targeted drug intervention experiments using pathway-specific agonists and inhibitors. Among the tested genes, only PLCB4 showed statistically significant regulation—its expression was suppressed by the Wnt pathway inhibitor 2-ABP and induced by the activator LON (**Figure 6B**). Consequently, PLCB4 was selected as a central target for further investigation. Immunohistochemical analysis of patient tumor samples revealed a significant inverse correlation between PLCB4 expression and glioma malignancy (**Figure 6C**), aligning well with our previous bioinformatic predictions (**Figure 3D**). To explore the molecular mechanisms underlying this association, we established a U87 glioma cell line with lentiviral-mediated PLCB4 overexpression and performed high-throughput RNA sequencing. Comparative transcriptomic analysis of the overexpression (OE), negative control (NC), and empty vector groups identified 235 upregulated and 65 downregulated genes (**Figure 6D**). KEGG pathway enrichment analysis showed that the upregulated genes were significantly clustered in the calcium ion signaling pathway (**Figure 6E**, **Supplementary Table S9**), consistent with the results of our drug perturbation experiments (**Figure 6B**). Gene Set Enrichment Analysis (GSEA) further indicated that PLCB4 overexpression suppressed pathways associated with cell proliferation (**Figure 6F**), a finding corroborated by EdU assays (**Figure 6G**, **Supplementary Figure S9**). Based on prior literature, these antiproliferative effects are likely mediated through modulation of the P53, PI3K, and RTK-RAS signaling pathways.

### 3.7 Screening of five drugs inhibiting GBM proliferation via molecular docking

**Figure 7:**
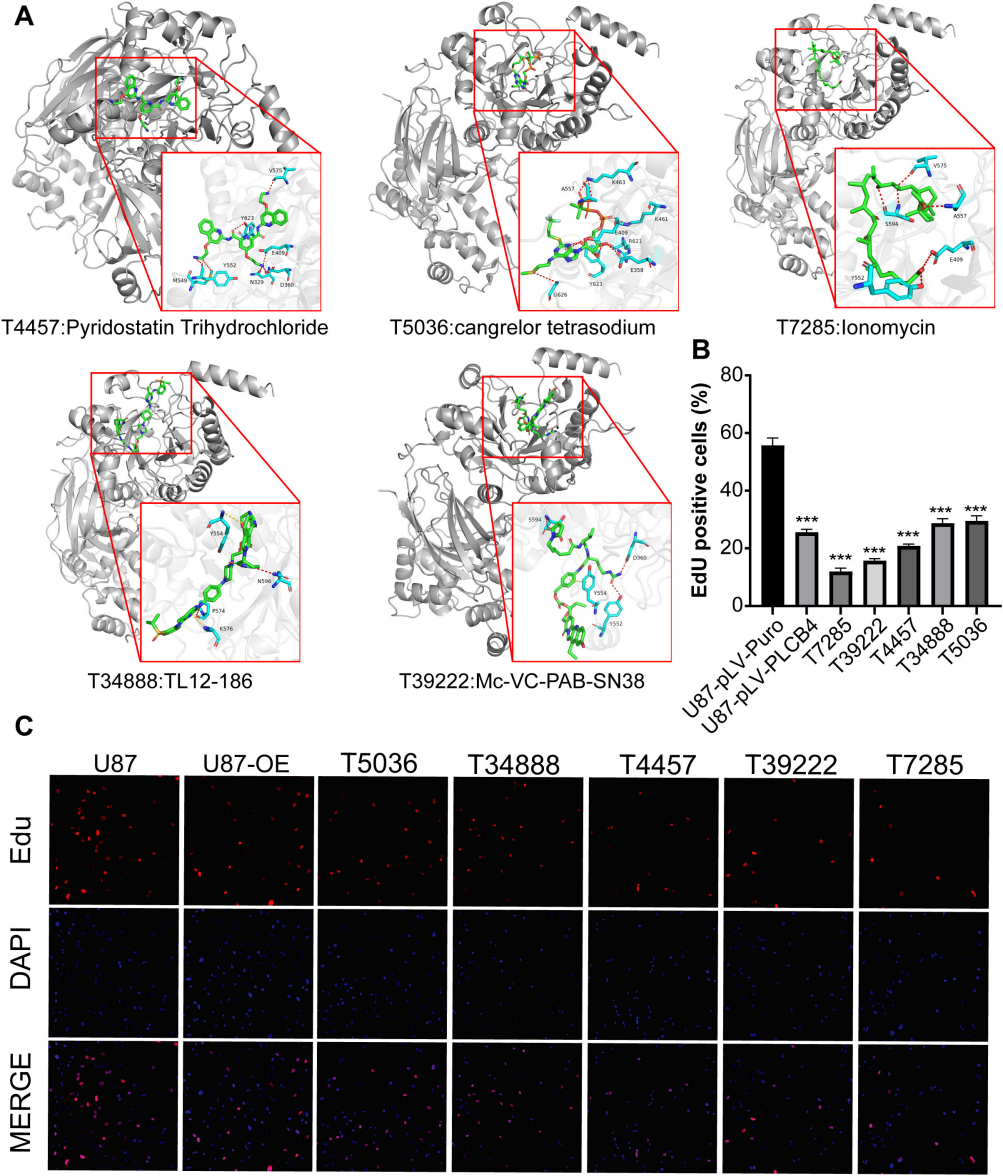
Identification of five PLCB4-Targeting drugs through molecular docking-based virtual screening and *in vitro* experiments. **(A)** Docking effect diagram of five drugs and the Alphafold predicted structure of PLCB4_HUMAN protein, including 2D and 3D schematic diagrams, revealing the binding mode of the compounds with the target. **(B)** EdU staining of the top 5 drugs with the highest GBM growth inhibition. **(C)** Fluorescence image of EdU proliferation experiment, blue fluorescence represents DAPI and red fluorescence represents proliferating U87 cells.

We next constructed a structural model of the PLCB4 protein and conducted molecular docking to screen 5,284 candidate compounds for binding affinity (**Supplementary Figure S10**). The top 84 molecules with the highest predicted affinities were shortlisted (**Supplementary Table S10**). Of these, the 50 highest-ranking compounds were subjected to cytotoxicity screening in GBM cells using the Cell Counting Kit-8 (CCK-8) assay. From this, the top five compounds with the strongest anti-GBM activity were selected for further validation (**Figure 7A**), and their efficacy in suppressing GBM cell proliferation was confirmed via EdU assays (**Figures 7B**, **C**).

## 4 Discussion

A total of 101 genes associated with GBM onset and progression—each exhibiting stable prognostic value and predictive relevance for immunotherapy response—were initially identified across seven GBM cohorts using WGCNA, immunotherapy response meta-analysis, and prognostic meta-analysis. These were further refined to 10 key genes with high predictive performance using four ML algorithms across six immunotherapy cohorts. This final gene set, termed the ProImmuML signature, includes: APCDD1, SOD3, ITPRIPL1, PLCB4, RAC2, THRA, RIN3, HM*G*B2, IMPA2, and CD274. Multidimensional analyses highlighted PLCB4 as a novel therapeutic target. This finding was supported by internal cohort data showing decreased PLCB4 expression with increasing tumor grade. Functional studies further revealed that PLCB4 overexpression activated the Wnt/Ca²^+^ signaling pathway and inhibited GBM cell proliferation. A protein structure model of PLCB4 was also constructed, and virtual screening identified five potential therapeutic compounds targeting this protein.

Literature review supports that several genes within the ProImmuML signature are established or emerging focal points in GBM research. CD274 is upregulated in most GBMs and is a principal target in immune checkpoint blockade therapies involving PD-1/PD-L1 interactions, offering renewed therapeutic promise for GBM patients (37). SOD3 knockdown has been shown to suppress M2-like macrophage polarization and inhibit GBM growth (38). Similarly, HMGB2 knockdown impairs GBM cell viability and invasiveness *in vitro*, reduces tumor volume *in vivo*, and enhances sensitivity to temozolomide, positioning it as a viable target for combinatorial therapy (39). These findings affirm the biological relevance and robustness of the gene selection process, providing novel targets for GBM treatment and broader pan-cancer applications.

Multi-omics analyses revealed that PLCB4 is frequently amplified and mutated at the genomic level across several GBM patient cohorts, yet its expression is consistently downregulated, indicating its potential role as a tumor suppressor. At the transcriptomic level, PLCB4 expression was significantly associated with key clinical variables, and a moderate positive correlation was observed between its mRNA and protein levels. Moreover, PLCB4 expression negatively correlated with immune regulatory processes and immune cell infiltration. Meta-analyses further demonstrated its high predictive accuracy for both immunotherapy response and overall prognosis, reinforcing its status as a stable protective factor in GBM—consistent with the integrated multi-omics findings.

The phospholipase C-beta (PLC-β) family is abundantly expressed in the nervous system, where it responds to neurotransmitter and hormone signaling. It plays a pivotal role in regulating tumor growth, angiogenesis, migration, invasion, and drug resistance by modulating cellular processes such as proliferation, differentiation, and apoptosis. Furthermore, PLC-β has been implicated in the pathogenesis of various neurological disorders, including Alzheimer’s disease, epilepsy, bipolar disorder, and autism spectrum disorder (40). Previous studies have shown that PLCB4-positive Purkinje neurons in mice exhibit marked transcriptional plasticity during sensorimotor learning, offering mechanistic insight into the differential susceptibility of these neurons to neurological disease (41). Frequent mutations in PLCB4 have been identified in GBM, leading to pro-oncogenic signaling and trafficking defects that may confer a survival advantage during tumor progression (36). In uveal melanoma, PLCB4 mediates complex downstream signaling cascades triggered by upstream driver mutations, contributing to tumors with diverse genetic and signaling characteristics (42). Integrated multi-omics analyses in pancreatic ductal adenocarcinoma revealed a significant correlation between PLCB4 mRNA expression and gene copy number variation, with tumors exhibiting both intra- and inter-tumoral heterogeneity in CNV patterns (43). In colorectal cancer, low PLCB4 expression has been associated with poorer patient survival, suggesting its potential as a novel therapeutic target (44). Collectively, these studies underscore the multifaceted role of PLCB4 across cancer types and highlight its promise as a target for cancer immunotherapy.

Aberrant Wnt signaling has been implicated in immune evasion by facilitating crosstalk between tumor cells and the TME, thereby promoting immune dysregulation and resistance to immunotherapies (45). PLCB4 is believed to modulate calcium signaling through the IP3 pathway, which in turn influences Wnt signaling activity. This mechanism may enhance the maintenance of cancer stem cell phenotypes and contribute to the development of an immunosuppressive microenvironment (46, 47). Elevated PLCB4 expression has also been linked to increased PD-L1 expression and the activation of immunosuppressive cell populations via the Wnt pathway, ultimately reducing the efficacy of immunotherapy (45). Targeting PLCB4 as a modulator of Wnt signaling has thus emerged as a promising strategy to reverse immunosuppression in GBM and improve therapeutic outcomes. Based on these insights, we propose a therapeutic strategy that targets PLCB4-mediated calcium signaling through Wnt/Ca²^+^ modulators. This approach holds potential to enhance the effectiveness of immunotherapy, particularly when integrated with conventional treatment regimens, thereby advancing personalized cancer care.

This study offers several strengths that enhance its significance and translational relevance in cancer research. Through a multi-tiered screening strategy and comprehensive multi-dimensional analyses, we identified genes with clear biological significance. We leveraged extensive multi-omics data from both public databases and our proprietary Gusu cohort, alongside multi-center validation studies, to ensure analytical robustness. Furthermore, we conducted preliminary *in vitro* experiments that supported the potential of PLCB4 as a therapeutic target in GBM, providing empirical, theoretical, and experimental substantiation of our conclusions. However, certain limitations remain. Although bioinformatic analyses suggested strong therapeutic potential, only a subset of identified targets underwent experimental validation, limiting definitive functional characterization. Additionally, most analyses were based on bulk transcriptomic data, with only partial validation using single-cell transcriptomics, precluding more granular resolution at the cellular level. The relatively small sample size of our in-house cohort may also constrain the generalizability of our findings to broader patient populations.

## 5 Conclusions

In this study, we developed a novel screening framework termed ProImmuML, which identified ten key genes with high predictive power for immunotherapy response and prognosis in GBM. Among these, *in vitro* experiments revealed PLCB4 as a novel therapeutic target, with expression inversely correlated with tumor grade. Functional assays indicated that PLCB4 inhibits GBM cell proliferation via activation of the Wnt/Ca²^+^ signaling pathway. Molecular docking analyses further identified five candidate compounds with high affinity for PLCB4. These findings underscore the therapeutic promise of PLCB4 and demonstrate the value of integrative methodologies in accelerating GBM treatment development. Future validation in preclinical and clinical settings is warranted.

## Data Availability

The original contributions presented in the study are publicly available. The study accession number in the European Nucleotide Archive (ENA) is ERP177400, and the sample identifiers are as follows: ERR15373468, ERR15373467, ERR15373256, ERR15373245, ERR15373244, ERR15373242, ERR15372959, ERR15372957, and ERR15372954.

## Supplementary material

Supplementary Figure 1 Clustering analysis of p53, PI3K, and RTK signaling pathways.**(A)** Clinical characteristics of seven GBM cohorts validated by three signaling pathways.**(B)** Gene dendrogram showing the WGCNA identified 12 gene modules.**(C)** Correlation between each gene module and the occurrence and development of GBM in three signaling pathways.

Supplementary Figure 2 Metascape of 101 Genes of intersection of three layers screening.

Supplementary Figure 3 Prognostic meta-analysis integrating LGG/GBM RNA-seq and microarray datasets.

Supplementary Figure 4 Transcriptomics, proteomics and post-translational modification data from the CPTAC database.**(A)** Differential expression analysis based on the CPTAC cohort showing the mRNA, protein and phosphorylation expression of the ProImmuML signature in seven cancer driver alterations compared to unaltered patients.**(B)** Correlation analysis between mRNA and protein expression of ProImmuML signature based on CPTAC cohort.

Supplementary Figure 5 Differential expression of the ProImmuML signature between 34 cancer tissues and adjacent normal tissues.

Supplementary Figure 6 Nested resampling used for hyperparameter tuning and calculating the average AUC value.

Supplementary Figure 7 The AUC of 31 combinations across five cohorts showed in heatmap. Combinations highlighted in red represent the inclusion of risk score.

Supplementary Figure 8 Composition and mechanism of Wnt signaling pathway.

Supplementary Figure 9 The quantitative graph for the EdU staining.

Supplementary Figure 10 Flowchart of drug screening.
