# Supplementary material for: Meta-analysis of multi-center transcriptomic profiles and machine learning reveal phospholipase Cβ4 as a Wnt/Ca²+ signaling mediator in glioblastoma immunotherapy
Source: Front Immunol. 2025 Aug 7;16:1610683. doi: 10.3389/fimmu.2025.1610683 (PMC12368592; doi:10.3389/fimmu.2025.1610683)
Supplement: Supplementary Figure 1 — Clustering analysis of p53, PI3K, and RTK signaling pathways.(A) Clinical characteristics of seven GBM cohorts validated by three signaling pathways.(B) Gene dendrogram showing the WGCNA identified 12 gene modules.(C) Correlation between each gene module and the occurrence and development of GBM in three signaling pathways. [file Image1.pdf]

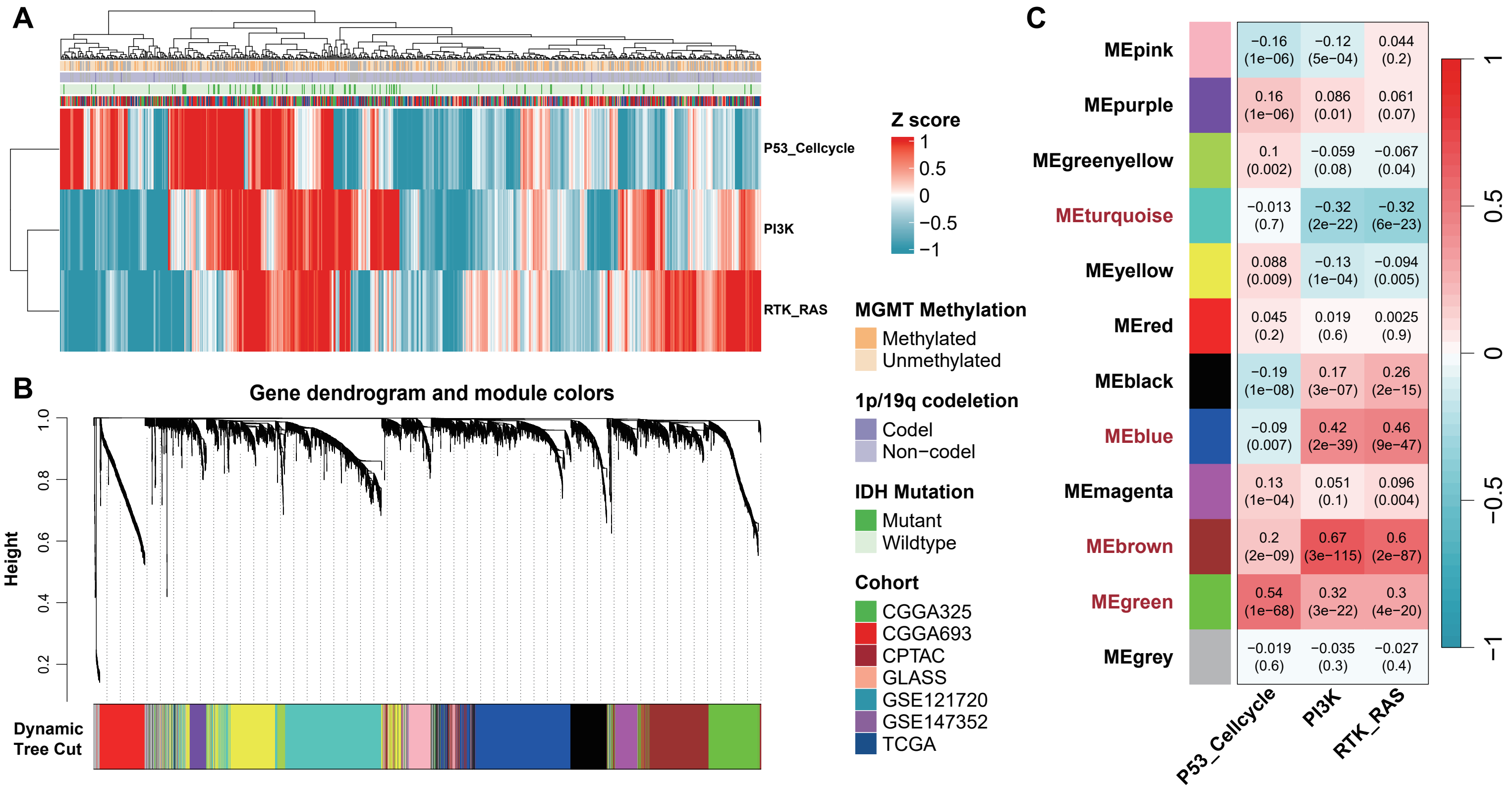
A
P53_Cellcycle
PI3K
RTK_RAS
Z score
1
0.5
0
−0.5
−1
MGMT Methylation
Methylated
Unmethylated
1p/19q codeletion
Codel
Non−codel
IDH Mutation
Mutant
Wildtype
Cohort
CGGA325
CGGA693
CPTAC
GLASS
GSE121720
GSE147352
TCGA
B
Gene dendrogram and module colors
Height
1.0
0.8
0.6
0.4
0.2
Dynamic Tree Cut
C
MEpink −0.16 (1e−06) −0.12 (5e−04) 0.044 (0.2)
MEpurple 0.16 (1e−06) 0.086 (0.01) 0.061 (0.07)
MEgreenyellow 0.1 (0.002) −0.059 (0.08) −0.067 (0.04)
MEturquoise −0.013 (0.7) −0.32 (2e−22) −0.32 (6e−23)
MEyellow 0.088 (0.009) −0.13 (1e−04) −0.094 (0.005)
MEred 0.045 (0.2) 0.019 (0.6) 0.0025 (0.9)
MEblack −0.19 (1e−08) 0.17 (3e−07) 0.26 (2e−15)
MEblue −0.09 (0.007) 0.42 (2e−39) 0.46 (9e−47)
MEmagenta 0.13 (1e−04) 0.051 (0.1) 0.096 (0.004)
MEbrown 0.2 (2e−09) 0.67 (3e−115) 0.6 (2e−87)
MEgreen 0.54 (1e−68) 0.32 (3e−22) 0.3 (4e−20)
MEgrey −0.019 (0.6) −0.035 (0.3) −0.027 (0.4)
P53_Cellcycle
PI3K
RTK_RAS
1
0.5
0
−0.5
−1

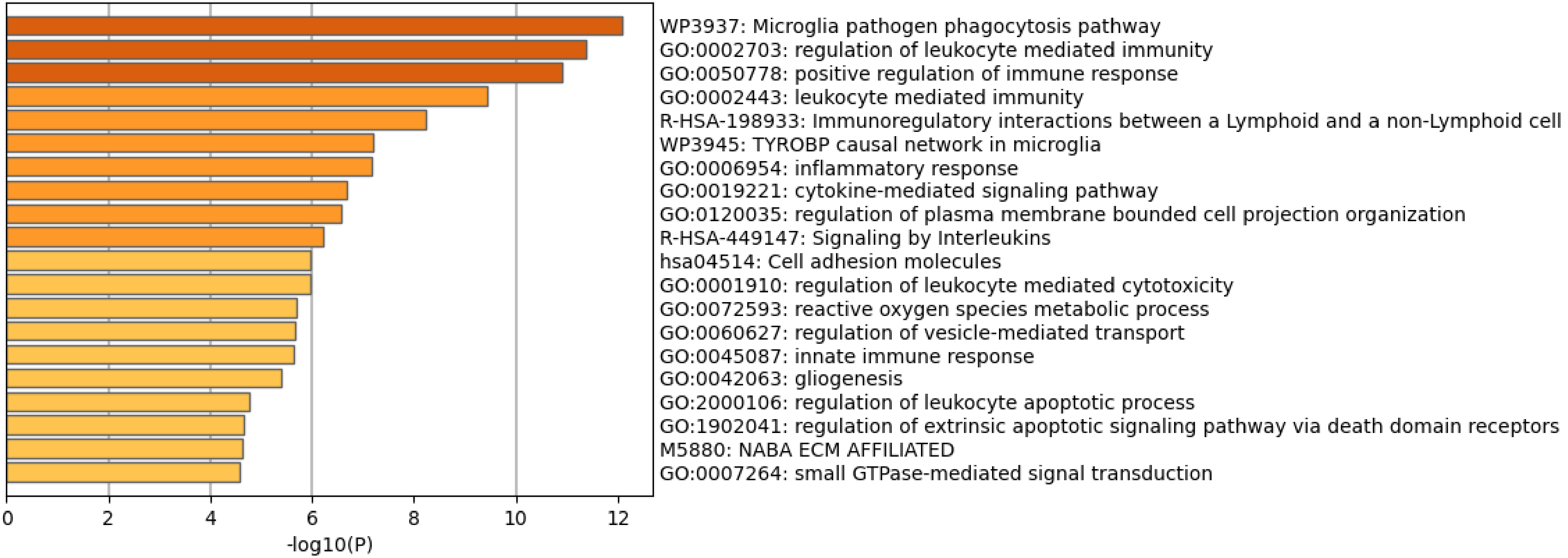
WP3937: Microglia pathogen phagocytosis pathway
GO:0002703: regulation of leukocyte mediated immunity
GO:0050778: positive regulation of immune response
GO:0002443: leukocyte mediated immunity
R-HSA-198933: Immunoregulatory interactions between a Lymphoid and a non-Lymphoid cell
WP3945: TYROBP causal network in microglia
GO:0006954: inflammatory response
GO:0019221: cytokine-mediated signaling pathway
GO:0120035: regulation of plasma membrane bounded cell projection organization
R-HSA-449147: Signaling by Interleukins
hsa04514: Cell adhesion molecules
GO:0001910: regulation of leukocyte mediated cytotoxicity
GO:0072593: reactive oxygen species metabolic process
GO:0060627: regulation of vesicle-mediated transport
GO:0045087: innate immune response
GO:0042063: gliogenesis
GO:2000106: regulation of leukocyte apoptotic process
GO:1902041: regulation of extrinsic apoptotic signaling pathway via death domain receptors
M5880: NABA ECM AFFILIATED
GO:0007264: small GTPase-mediated signal transduction
0
2
4
6
8
10
12
-log10(P)

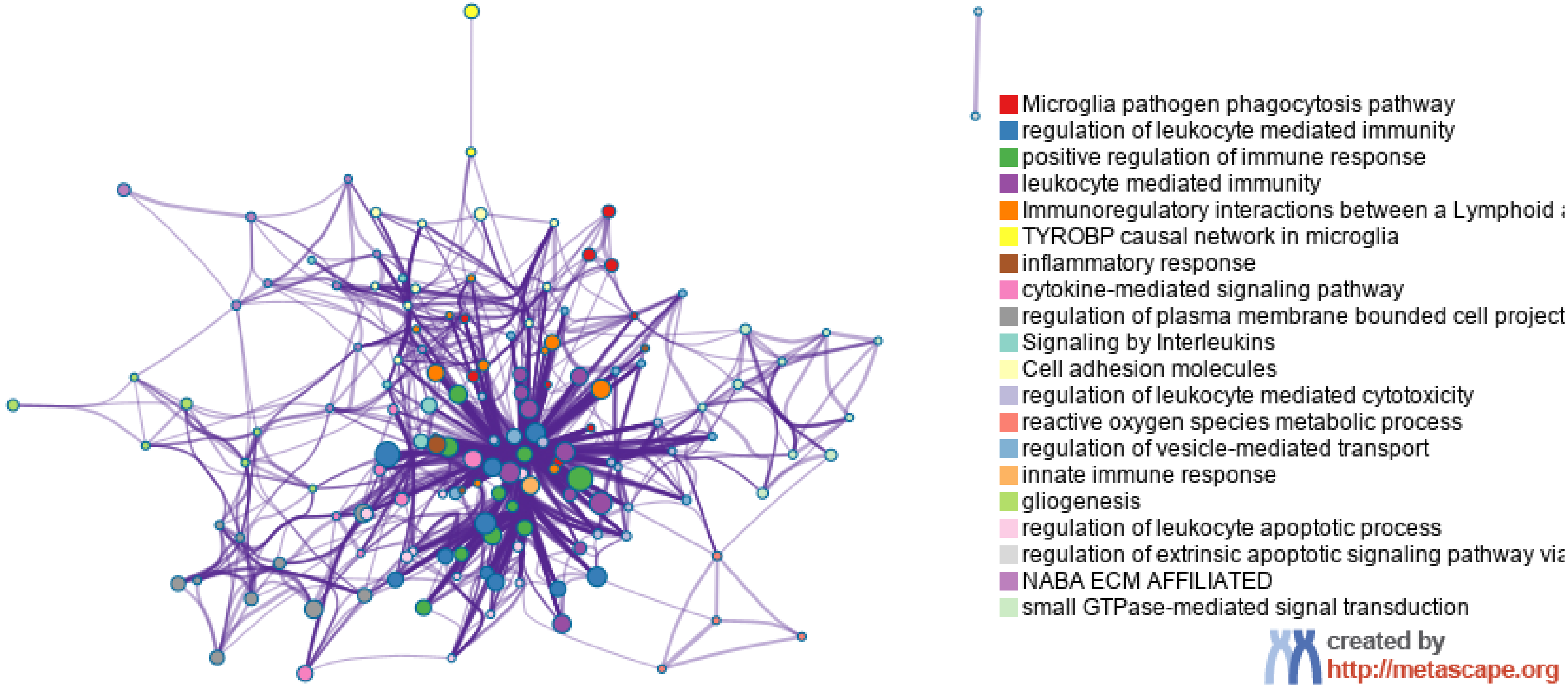
Microglia pathogen phagocytosis pathway
regulation of leukocyte mediated immunity
positive regulation of immune response
leukocyte mediated immunity
Immunoregulatory interactions between a Lymphoid
TYROBP causal network in microglia
inflammatory response
cytokine-mediated signaling pathway
regulation of plasma membrane bounded cell project
Signaling by Interleukins
Cell adhesion molecules
regulation of leukocyte mediated cytotoxicity
reactive oxygen species metabolic process
regulation of vesicle-mediated transport
innate immune response
gliogenesis
regulation of leukocyte apoptotic process
regulation of extrinsic apoptotic signaling pathway via
NABA ECM AFFILIATED
small GTPase-mediated signal transduction
created by
http://metascape.org

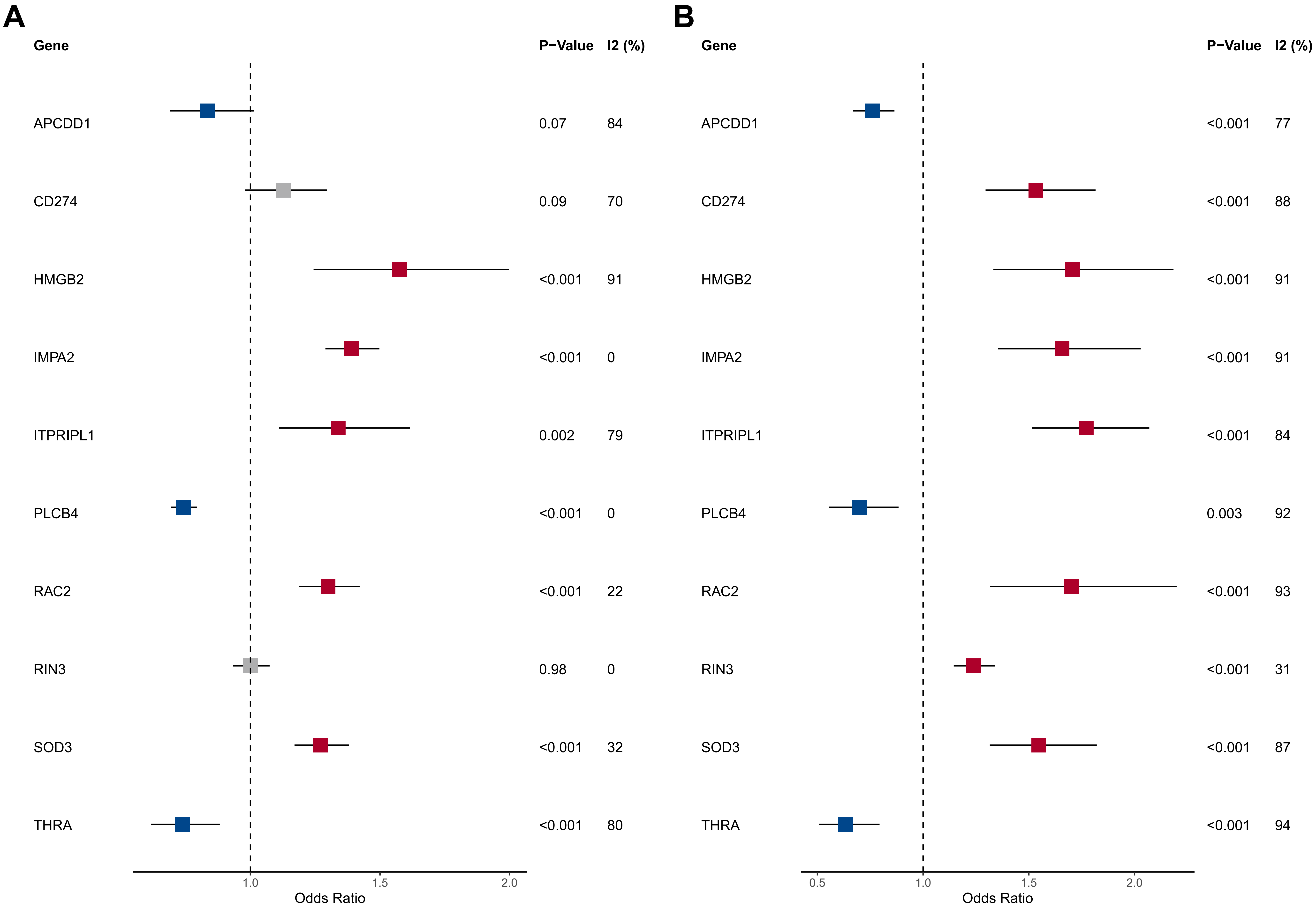
A
Gene
P−Value
I2 (%)
APCDD1
0.07
84
CD274
0.09
70
HMGB2
<0.001
91
IMPA2
<0.001
0
ITPRIPL1
0.002
79
PLCB4
<0.001
0
RAC2
<0.001
22
RIN3
0.98
0
SOD3
<0.001
32
THRA
<0.001
80
1.0
1.5
2.0
Odds Ratio
B
Gene
P−Value
I2 (%)
APCDD1
<0.001
77
CD274
<0.001
88
HMGB2
<0.001
91
IMPA2
<0.001
91
ITPRIPL1
<0.001
84
PLCB4
0.003
92
RAC2
<0.001
93
RIN3
<0.001
31
SOD3
<0.001
87
THRA
<0.001
94
0.5
1.0
1.5
2.0
Odds Ratio

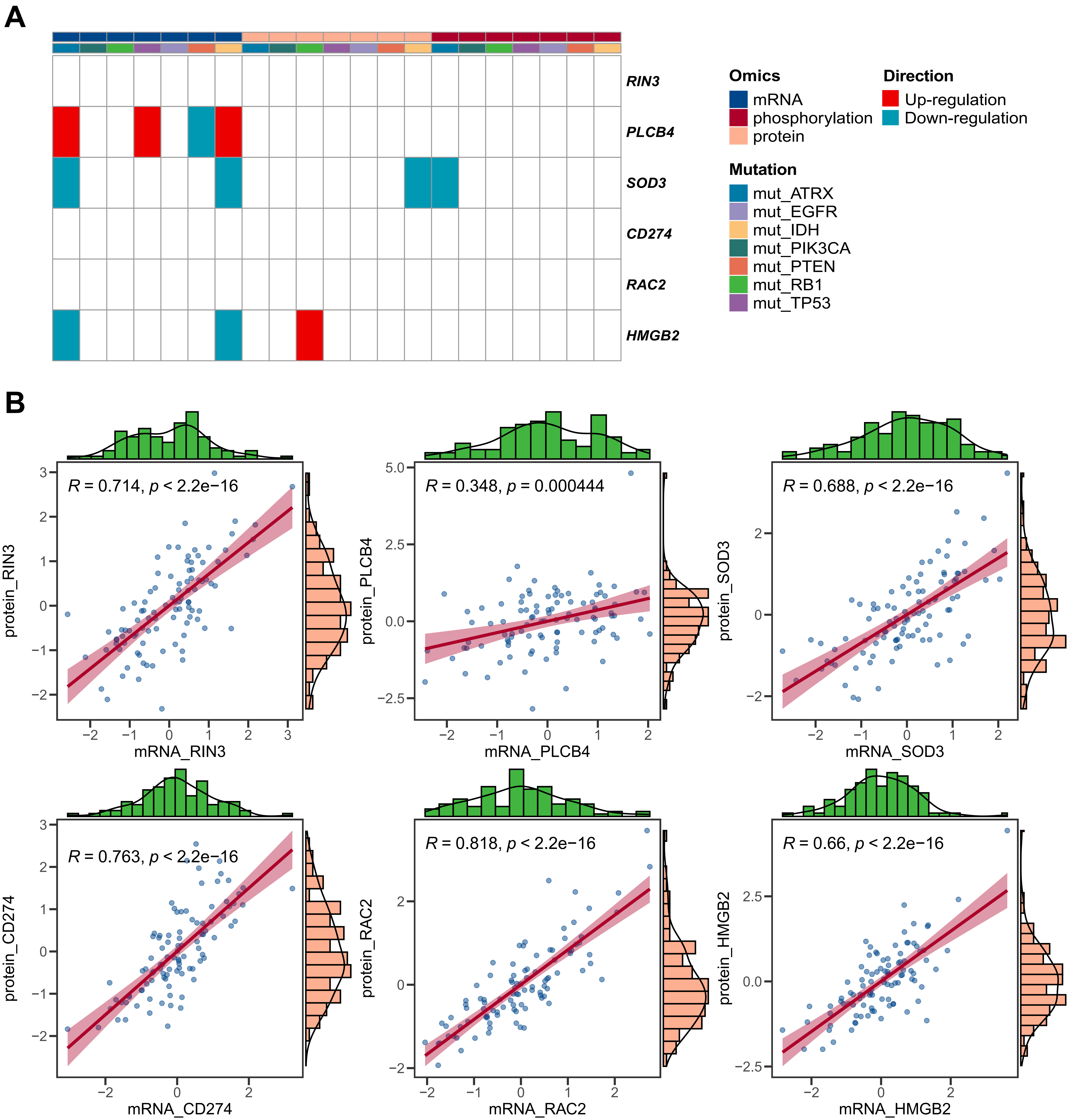

A
RIN3
PLCB4
SOD3
CD274
RAC2
HMGB2
Omics
mRNA
phosphorylation
protein
Direction
Up-regulation
Down-regulation
Mutation
mut_ATRX
mut_EGFR
mut_IDH
mut_PIK3CA
mut_PTEN
mut_RB1
mut_TP53
B
R = 0.714, p < 2.2e−16
protein_RIN3
mRNA_RIN3
R = 0.348, p = 0.000444
protein_PLCB4
mRNA_PLCB4
R = 0.688, p < 2.2e−16
protein_SOD3
mRNA_SOD3
R = 0.763, p < 2.2e−16
protein_CD274
mRNA_CD274
R = 0.818, p < 2.2e−16
protein_RAC2
mRNA_RAC2
R = 0.66, p < 2.2e−16
protein_HMGB2
mRNA_HMGB2

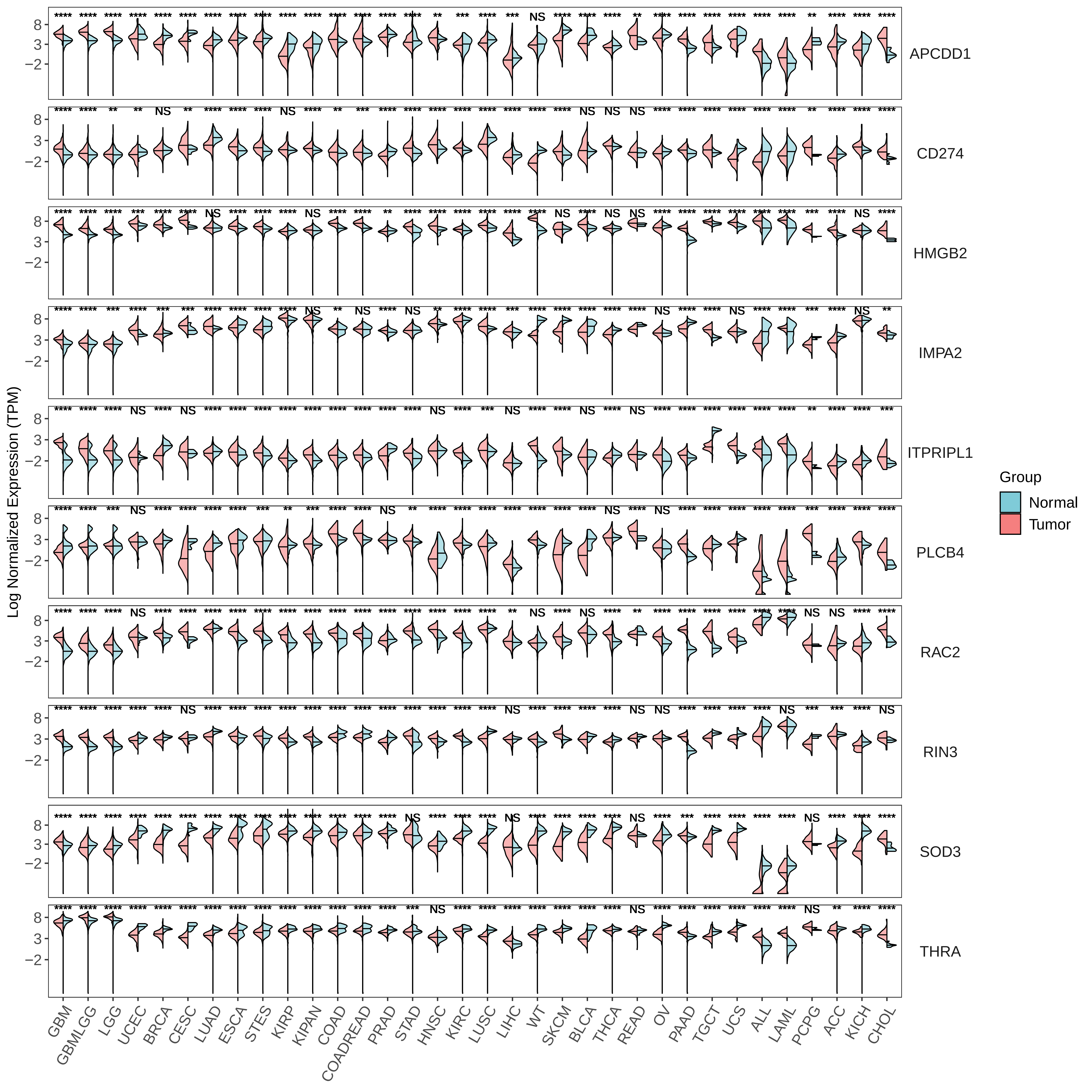

Log Normalized Expression (TPM)
APCDD1
CD274
HMGB2
IMPA2
ITPRIPL1
PLCB4
RAC2
RIN3
SOD3
THRA
Group
Normal
Tumor
GBM
GBMLGG
LGG
UCEC
BRCA
CESC
LUAD
ESCA
STES
KIRP
KIPAN
COAD
COADREAD
PRAD
STAD
HNSC
KIRC
LUSC
LIHC
WT
SKCM
BLCA
THCA
READ
OV
PAAD
TGCT
UCS
ALL
LAML
PCPG
ACC
KICH
CHOL

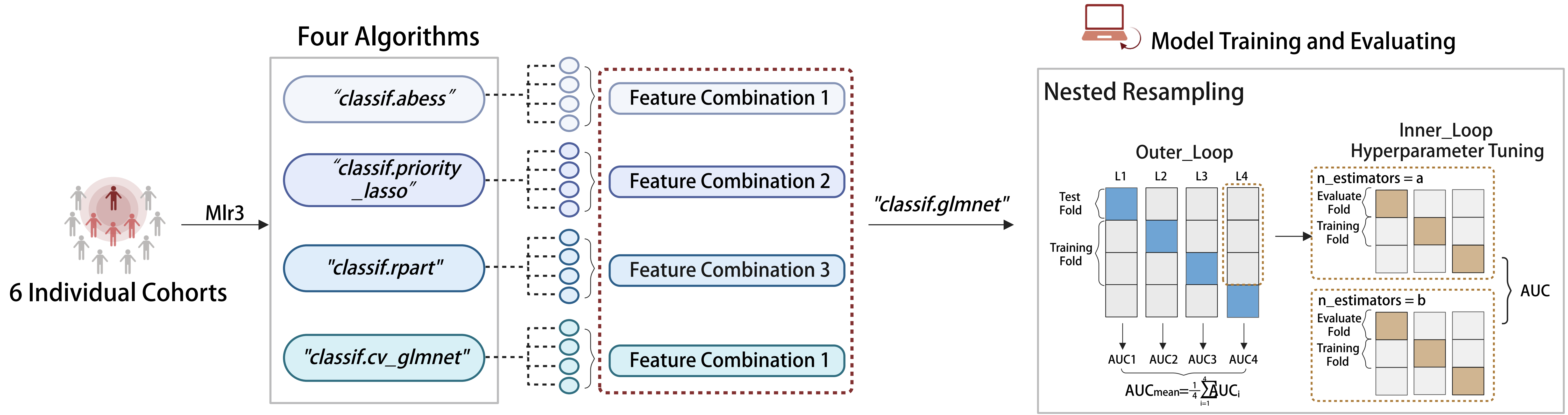

6 Individual Cohorts
Mlr3
Four Algorithms
"classif.abess"
"classif.priority_lasso"
"classif.rpart"
"classif.cv_glmnet"
Feature Combination 1
Feature Combination 2
Feature Combination 3
Feature Combination 1
"classif.glmnet"
Model Training and Evaluating
Nested Resampling
Outer_Loop
L1
L2
L3
L4
Test Fold
Training Fold
AUC1
AUC2
AUC3
AUC4
AUCmean=1/4 ∑ AUCi
Inner_Loop
Hyperparameter Tuning
n_estimators = a
Evaluate Fold
Training Fold
n_estimators = b
Evaluate Fold
Training Fold
AUC

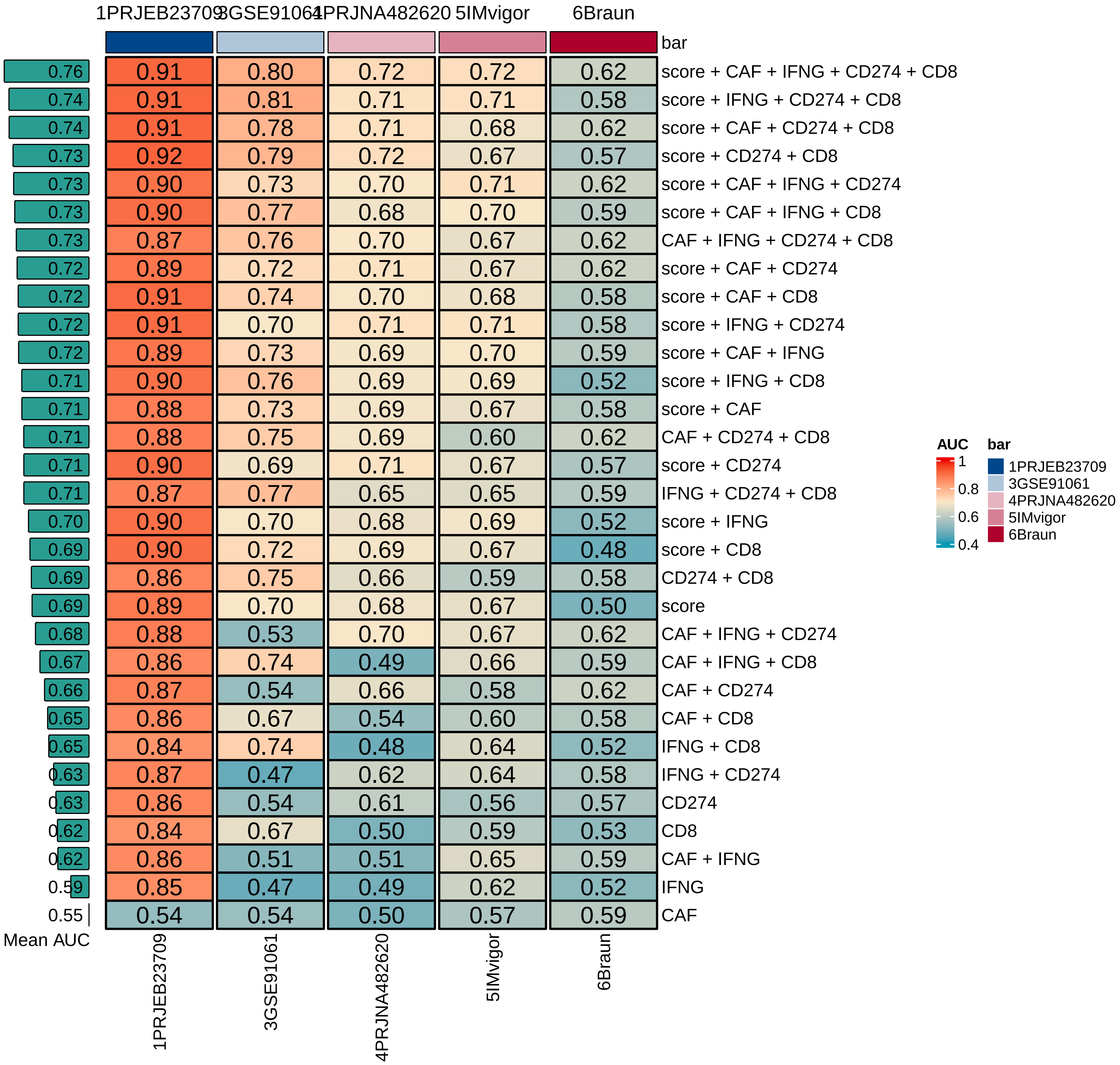
1PRJEB23709
3GSE91061
4PRJNA482620
5IMvigor
6Braun
bar
0.76 0.91 0.80 0.72 0.72 0.62 score + CAF + IFNG + CD274 + CD8
0.74 0.91 0.81 0.71 0.71 0.58 score + IFNG + CD274 + CD8
0.74 0.91 0.78 0.71 0.68 0.62 score + CAF + CD274 + CD8
0.73 0.92 0.79 0.72 0.67 0.57 score + CD274 + CD8
0.73 0.90 0.73 0.70 0.71 0.62 score + CAF + IFNG + CD274
0.73 0.90 0.77 0.68 0.70 0.59 score + CAF + IFNG + CD8
0.73 0.87 0.76 0.70 0.67 0.62 CAF + IFNG + CD274 + CD8
0.72 0.89 0.72 0.71 0.67 0.62 score + CAF + CD274
0.72 0.91 0.74 0.70 0.68 0.58 score + CAF + CD8
0.72 0.91 0.70 0.71 0.71 0.58 score + IFNG + CD274
0.72 0.89 0.73 0.69 0.70 0.59 score + CAF + IFNG
0.71 0.90 0.76 0.69 0.69 0.52 score + IFNG + CD8
0.71 0.88 0.73 0.69 0.67 0.58 score + CAF
0.71 0.88 0.75 0.69 0.60 0.62 CAF + CD274 + CD8
0.71 0.90 0.69 0.71 0.67 0.57 score + CD274
0.71 0.87 0.77 0.65 0.65 0.59 IFNG + CD274 + CD8
0.70 0.90 0.70 0.68 0.69 0.52 score + IFNG
0.69 0.90 0.72 0.69 0.67 0.48 score + CD8
0.69 0.86 0.75 0.66 0.59 0.58 CD274 + CD8
0.69 0.89 0.70 0.68 0.67 0.50 score
0.68 0.88 0.53 0.70 0.67 0.62 CAF + IFNG + CD274
0.67 0.86 0.74 0.49 0.66 0.59 CAF + IFNG + CD8
0.66 0.87 0.54 0.66 0.58 0.62 CAF + CD274
0.65 0.86 0.67 0.54 0.60 0.58 CAF + CD8
0.65 0.84 0.74 0.48 0.64 0.52 IFNG + CD8
0.63 0.87 0.47 0.62 0.64 0.58 IFNG + CD274
0.63 0.86 0.54 0.61 0.56 0.57 CD274
0.62 0.84 0.67 0.50 0.59 0.53 CD8
0.62 0.86 0.51 0.51 0.65 0.59 CAF + IFNG
0.59 0.85 0.47 0.49 0.62 0.52 IFNG
0.55 0.54 0.54 0.50 0.57 0.59 CAF
Mean AUC
1PRJEB23709
3GSE91061
4PRJNA482620
5IMvigor
6Braun
AUC
1
0.8
0.6
0.4
bar
1PRJEB23709
3GSE91061
4PRJNA482620
5IMvigor
6Braun

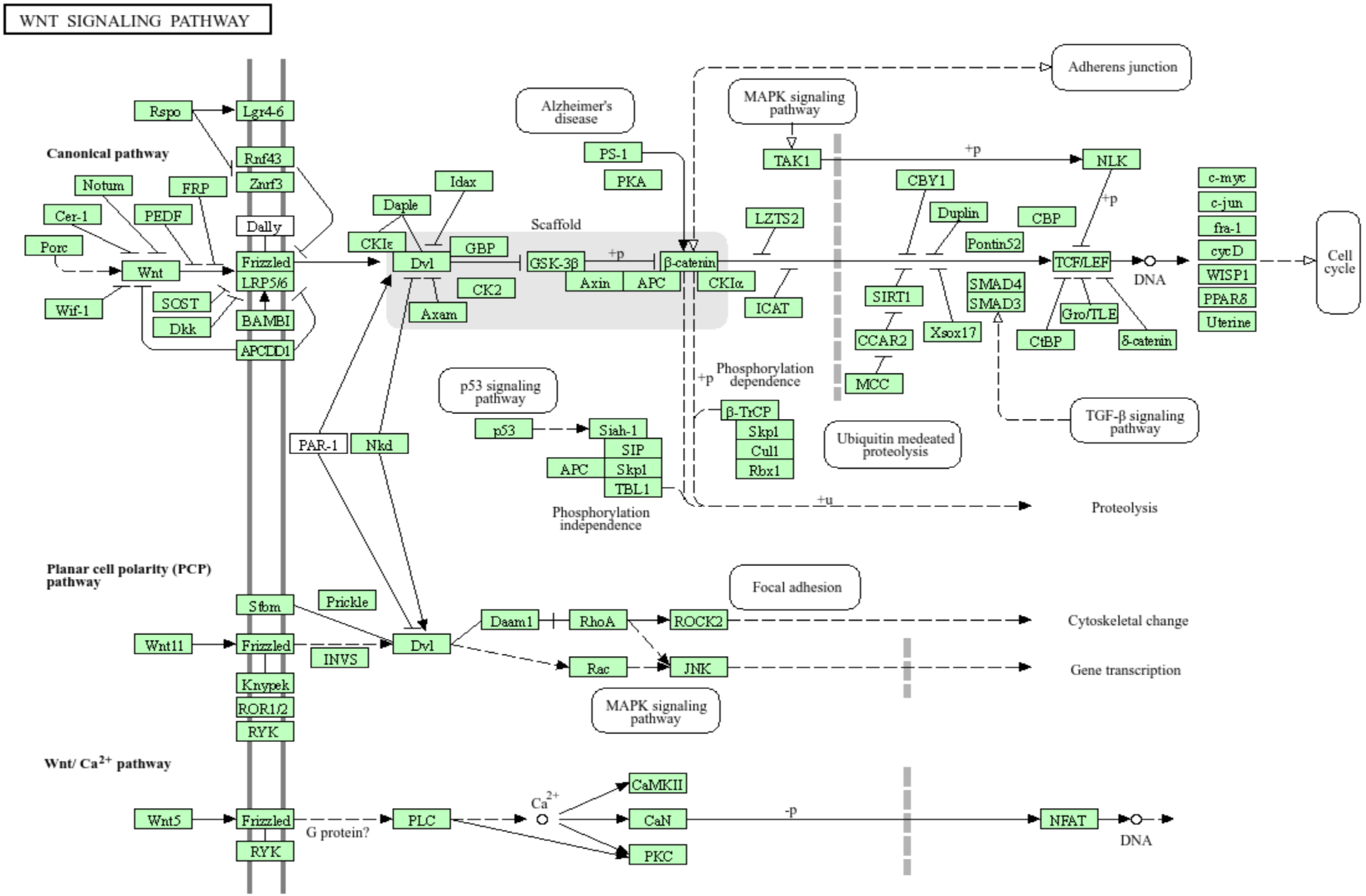

04310 8/9/23
(c) Kanehisa Laboratories

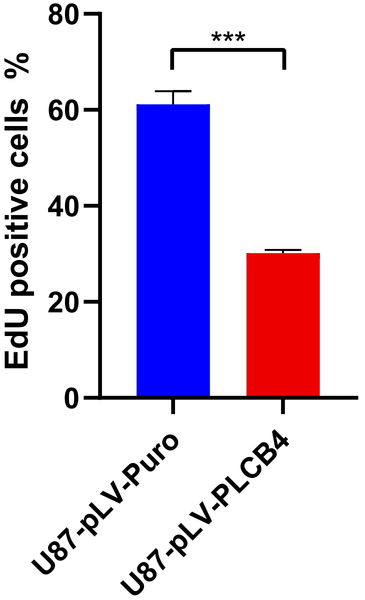

EdU positive cells %
80
60
40
20
0
***
U87-pLV-Puro
U87-pLV-PLCB4

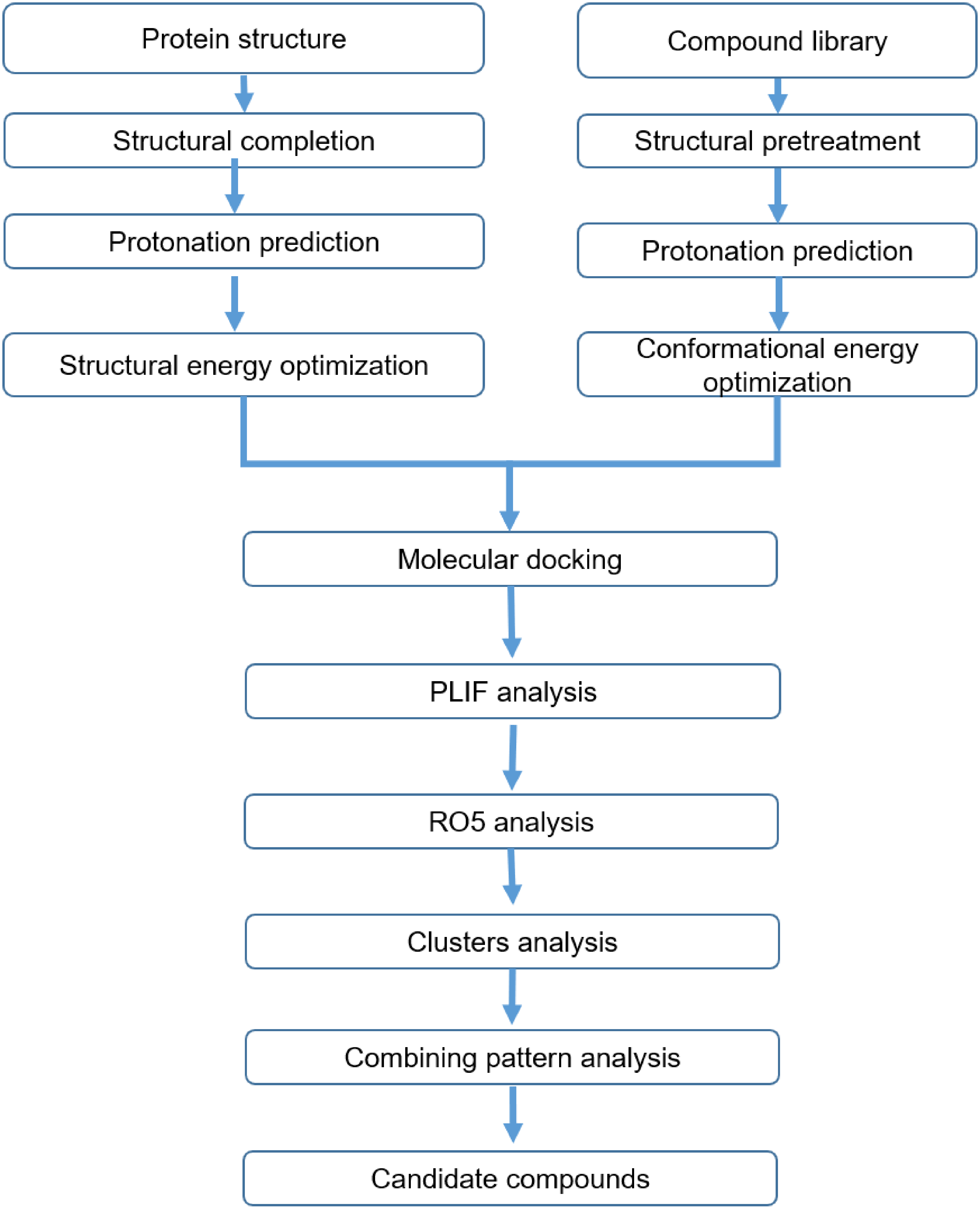
Protein structure
Structural completion
Protonation prediction
Structural energy optimization
Compound library
Structural pretreatment
Protonation prediction
Conformational energy optimization
Molecular docking
PLIF analysis
RO5 analysis
Clusters analysis
Combining pattern analysis
Candidate compounds
