# Supplementary figures and images for: Meta-analysis of multi-center transcriptomic profiles and machine learning reveal phospholipase Cβ4 as a Wnt/Ca²+ signaling mediator in glioblastoma immunotherapy
Source: Front Immunol. 2025 Aug 7;16:1610683. doi: 10.3389/fimmu.2025.1610683 (PMC12368592; doi:10.3389/fimmu.2025.1610683)

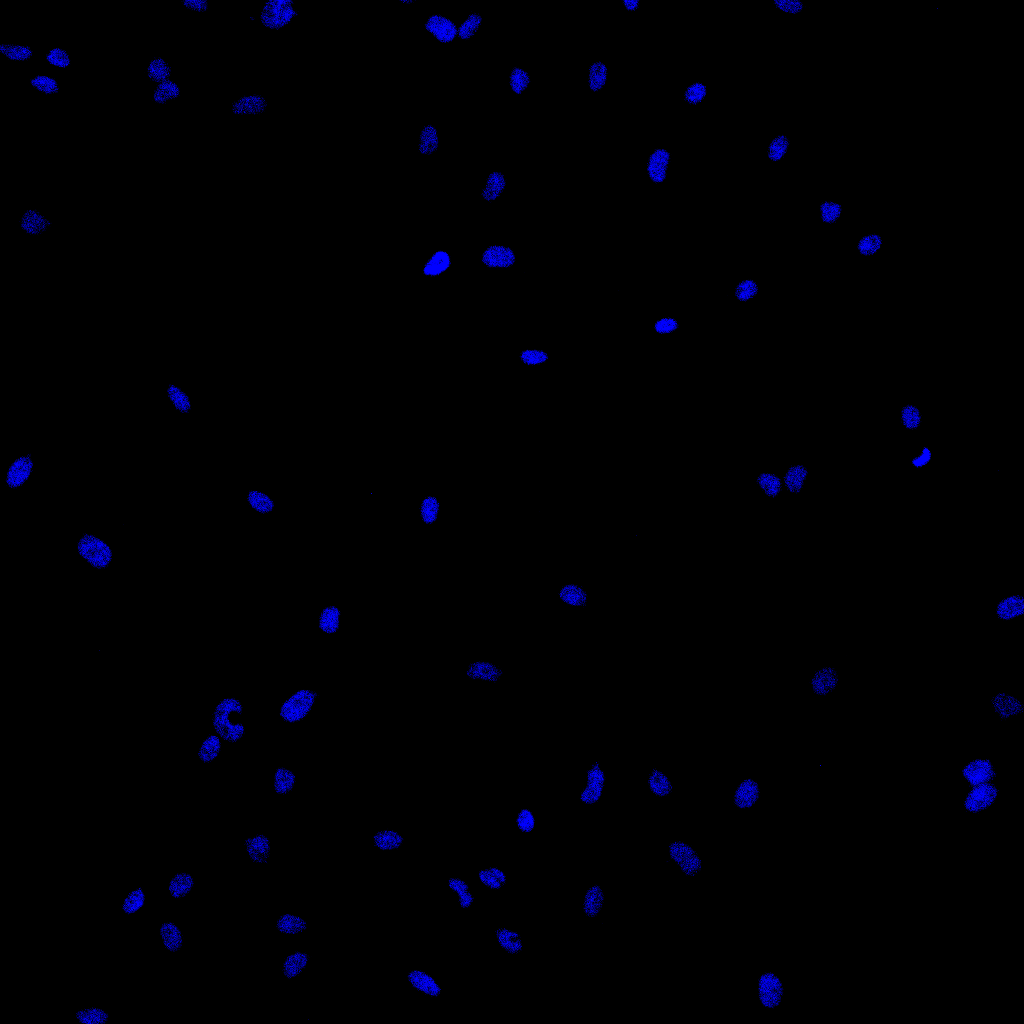

Supplement: Supplementary file 2 [file Presentation1.zip › EDU after drug screening/T34888/1 DAPI.tif]

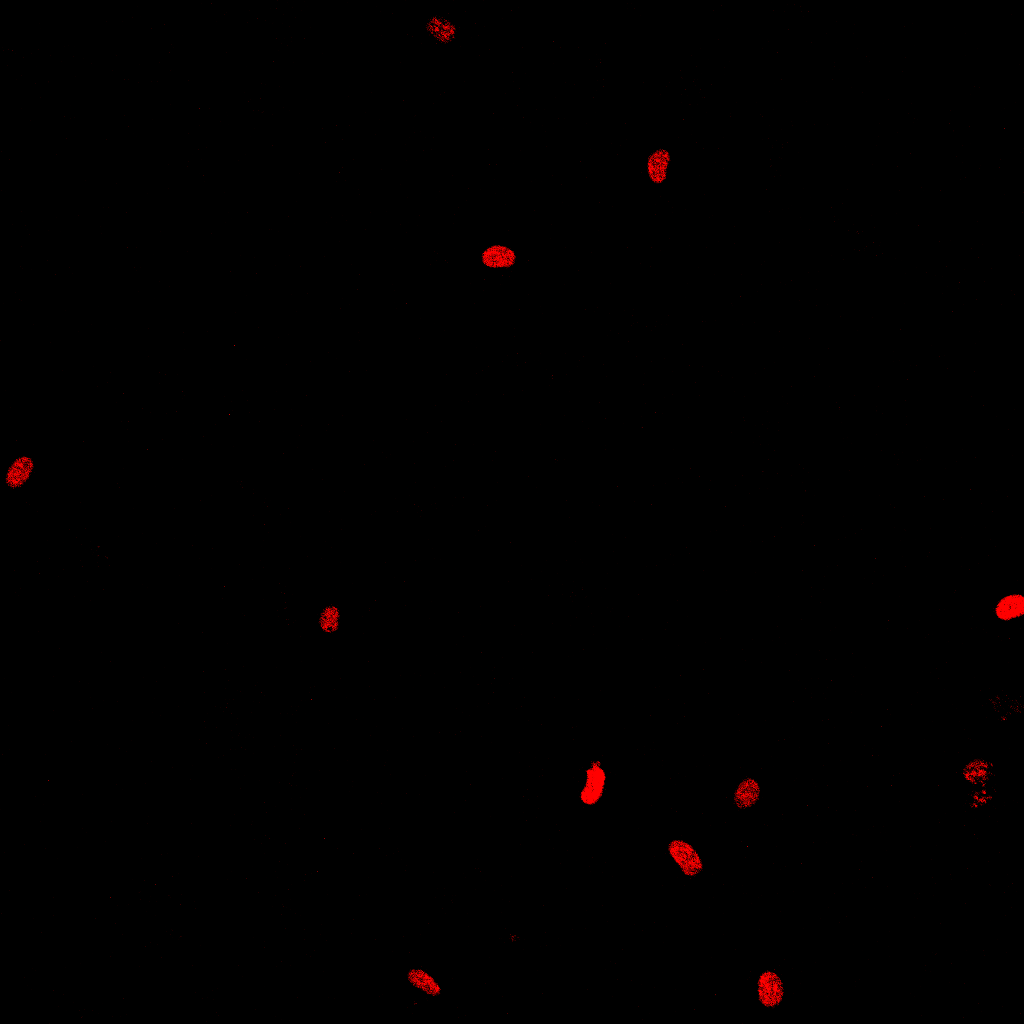

Supplement: Supplementary file 2 [file Presentation1.zip › EDU after drug screening/T34888/1 Edu.tif]

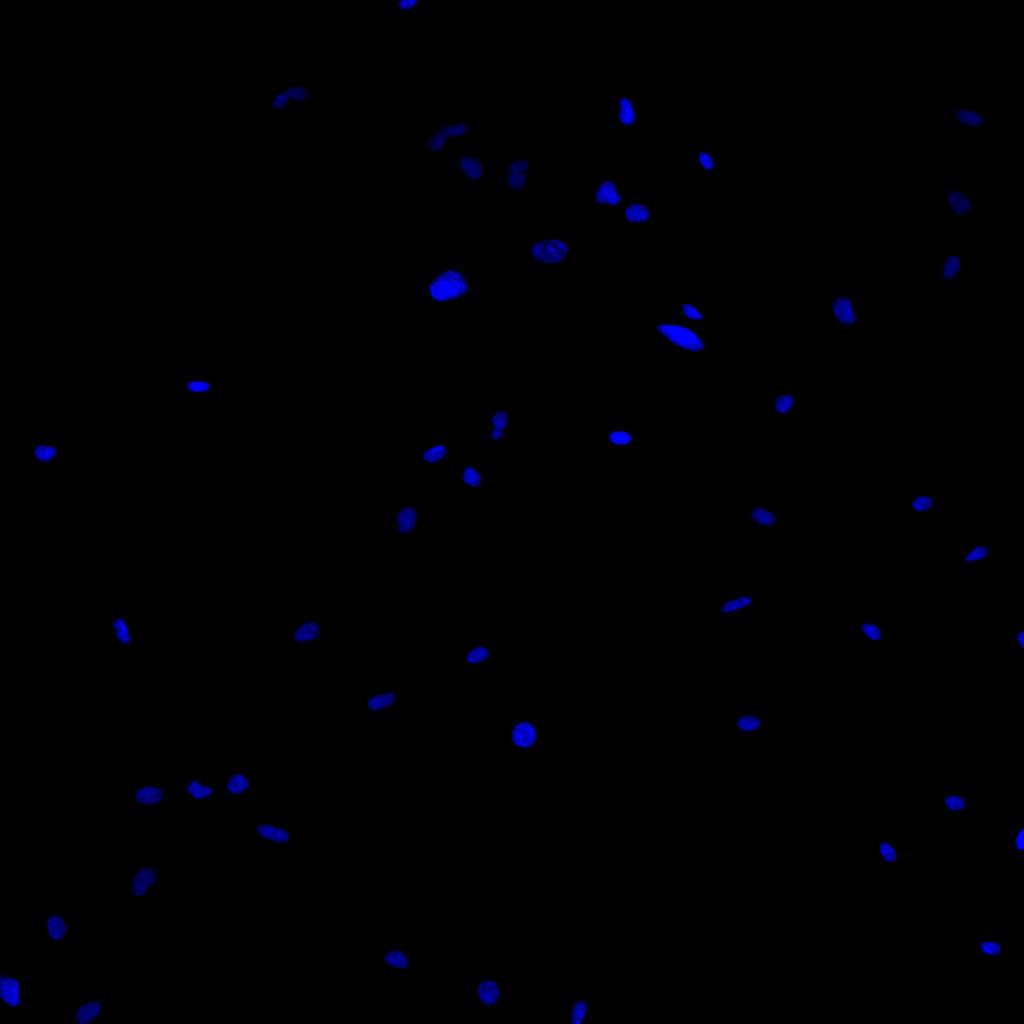

Supplement: Supplementary file 2 [file Presentation1.zip › EDU after drug screening/T34888/2 DAPI.tif]

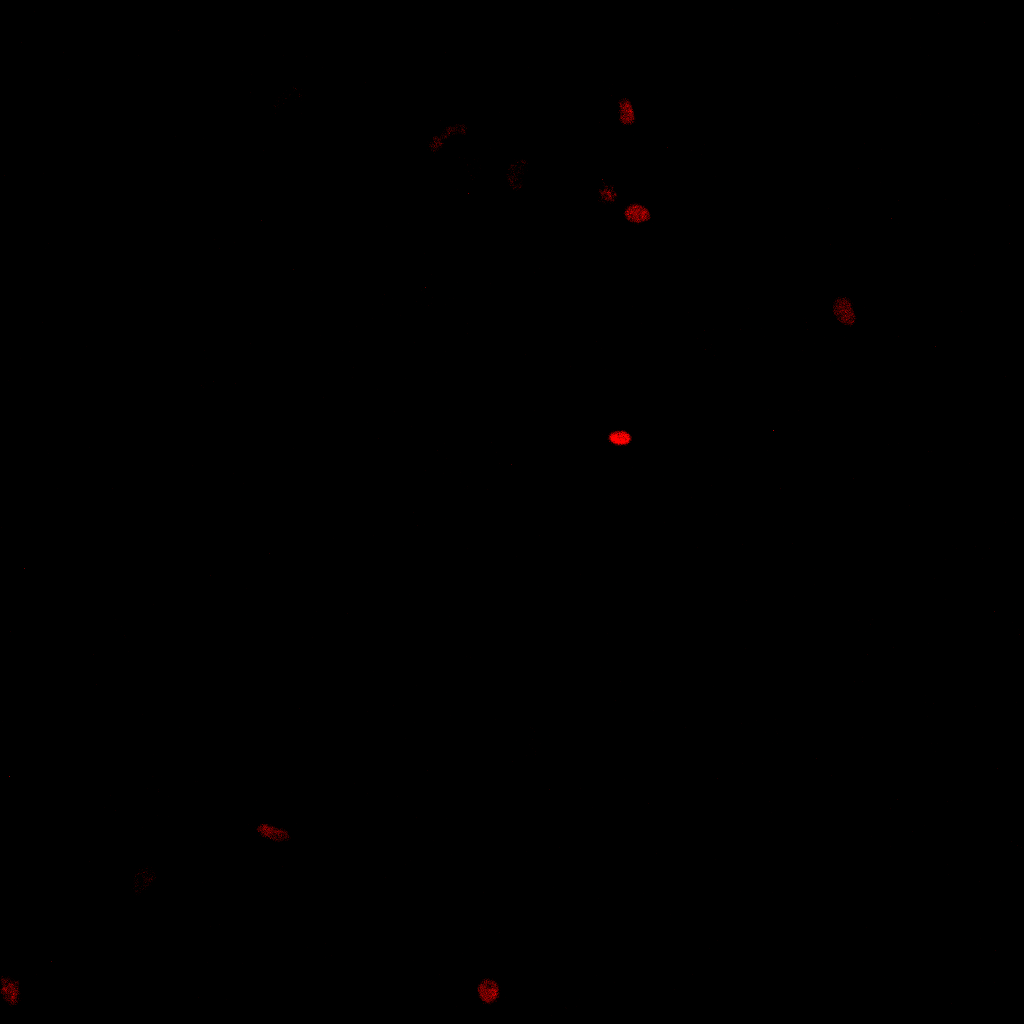

Supplement: Supplementary file 2 [file Presentation1.zip › EDU after drug screening/T34888/2 Edu.tif]

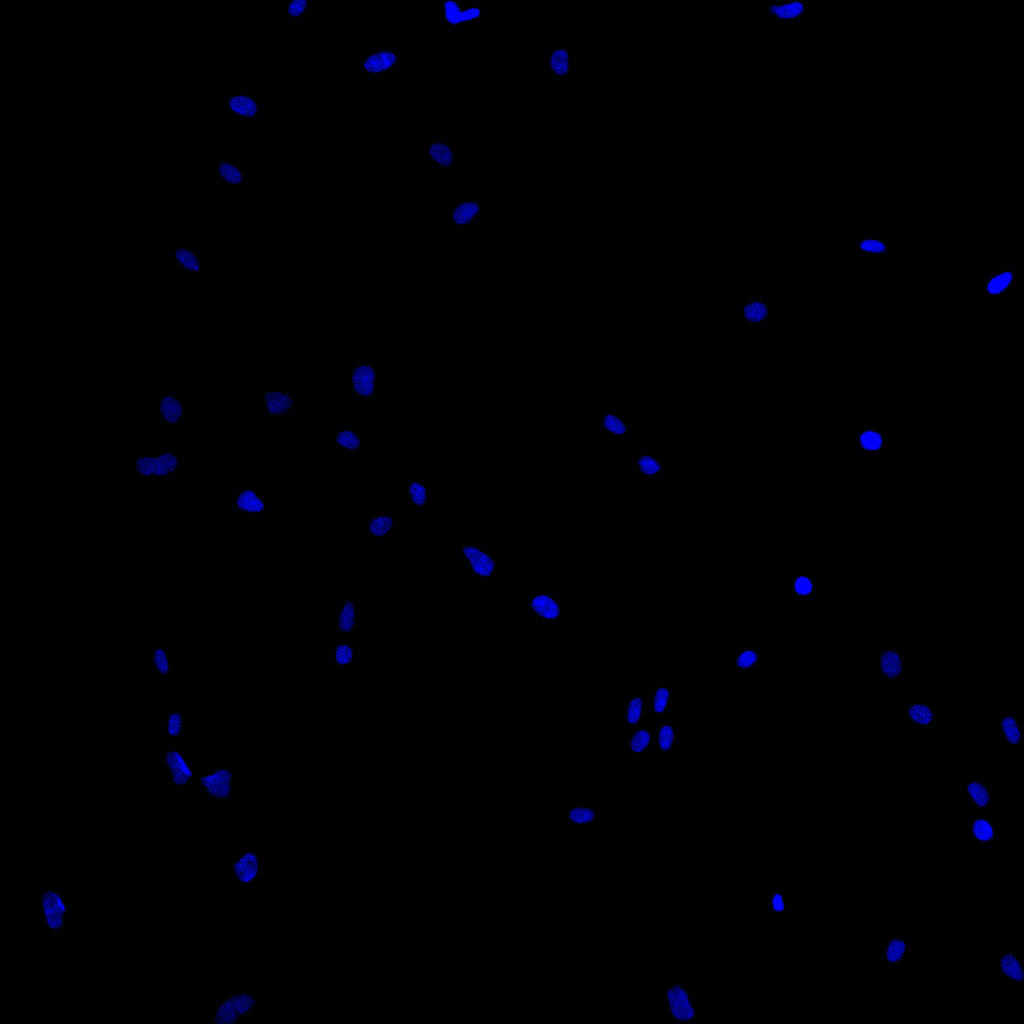

Supplement: Supplementary file 2 [file Presentation1.zip › EDU after drug screening/T34888/3 DAPI.tif]

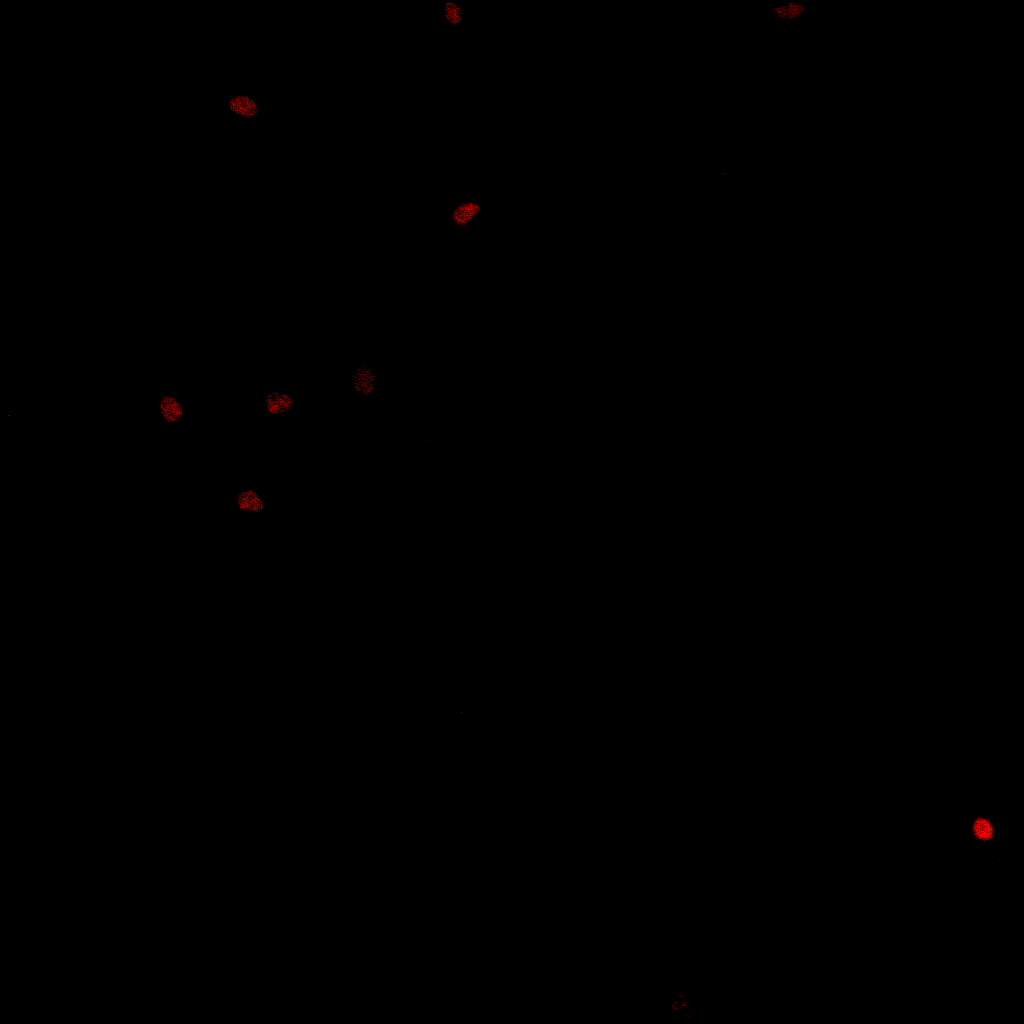

Supplement: Supplementary file 2 [file Presentation1.zip › EDU after drug screening/T34888/3 Edu.tif]

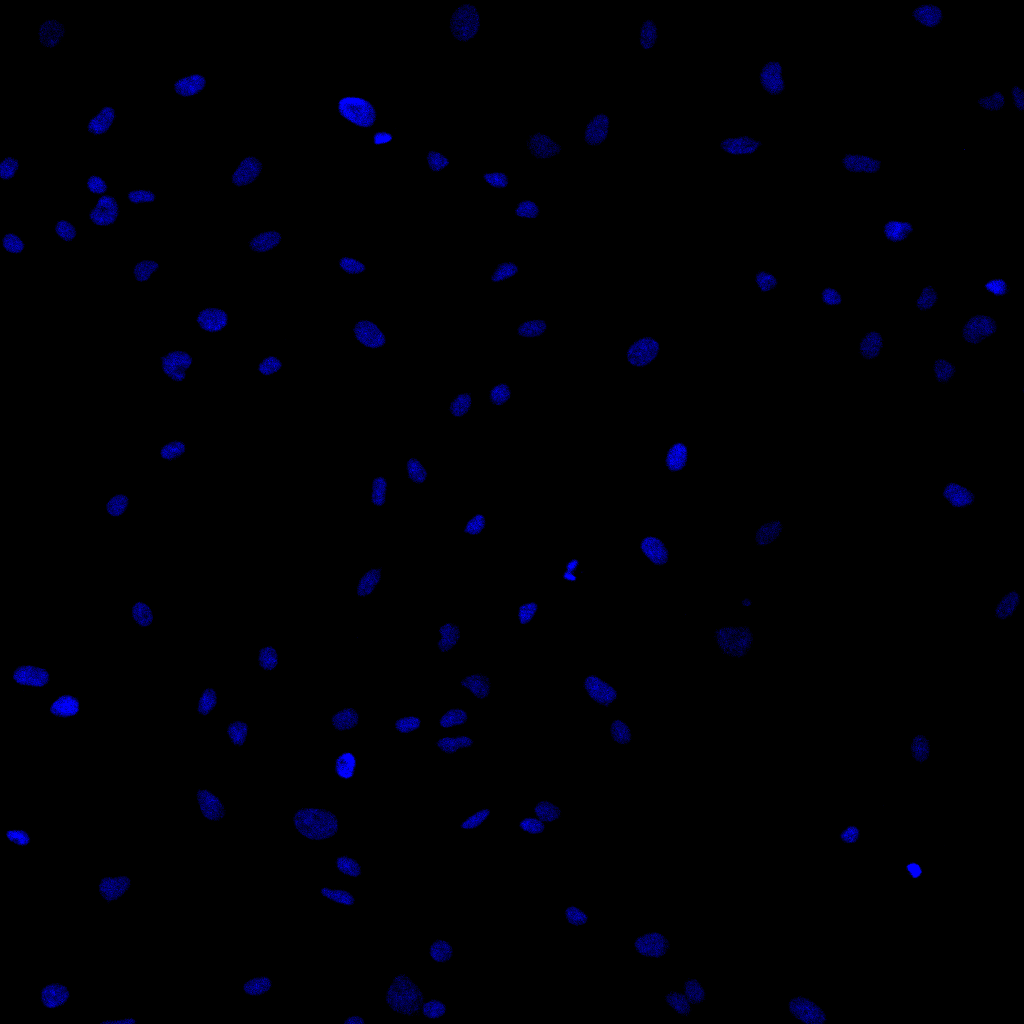

Supplement: Supplementary file 2 [file Presentation1.zip › EDU after drug screening/T39222/1 DAPI.tif]

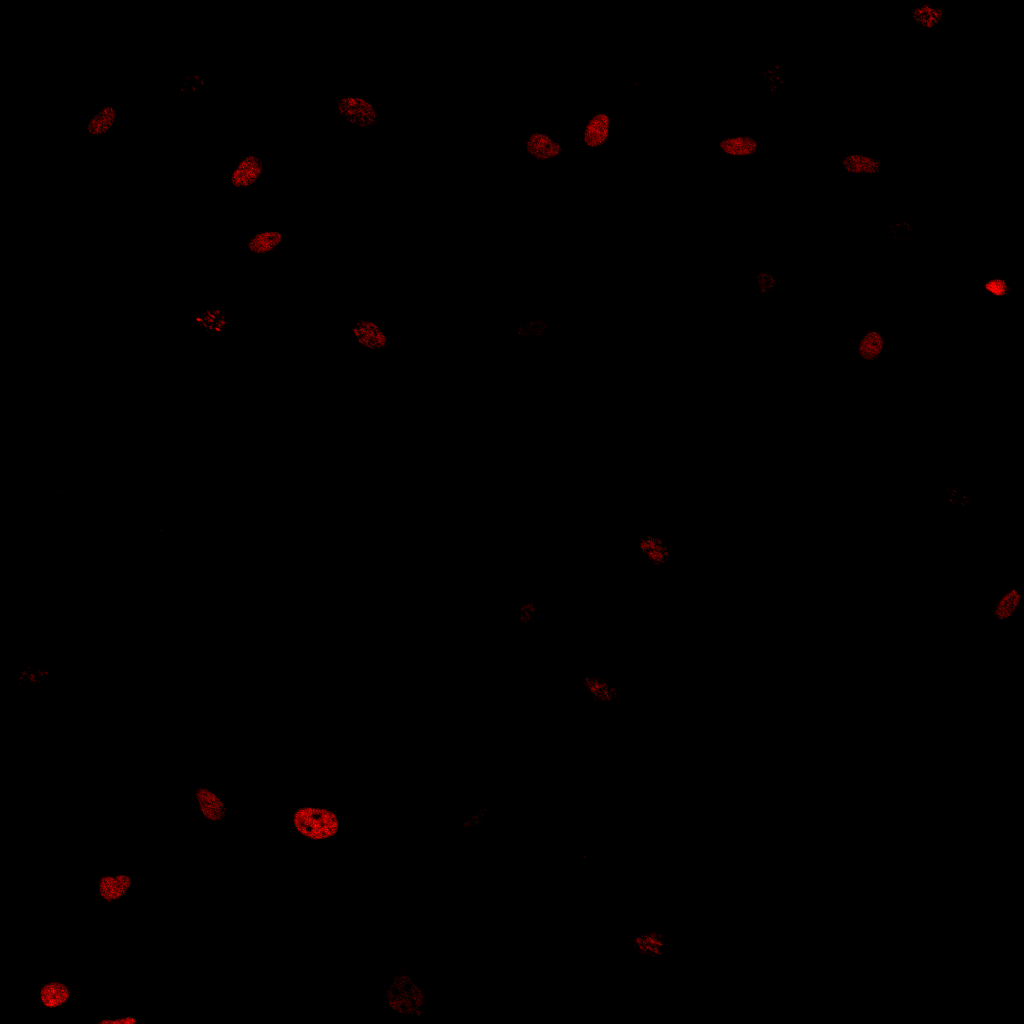

Supplement: Supplementary file 2 [file Presentation1.zip › EDU after drug screening/T39222/1 Edu.tif]

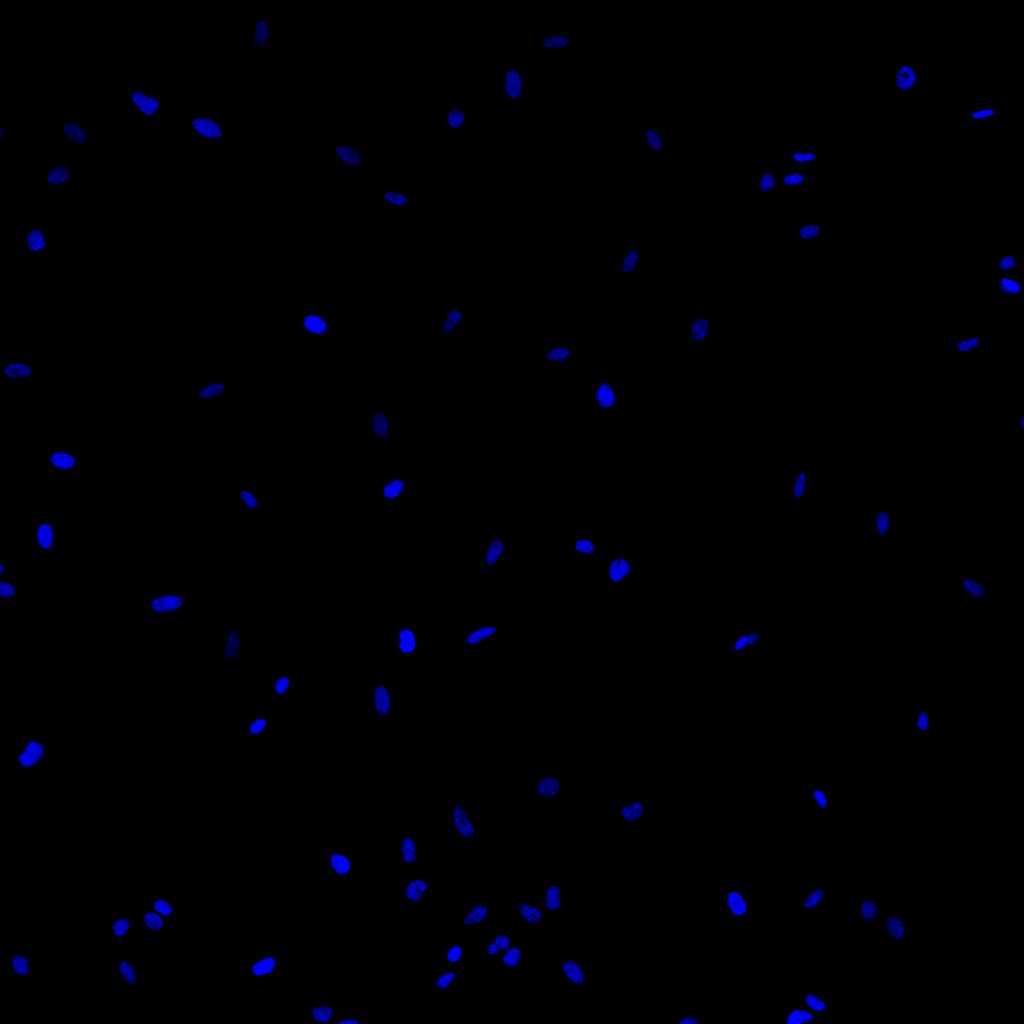

Supplement: Supplementary file 2 [file Presentation1.zip › EDU after drug screening/T39222/2 DAPI.tif]

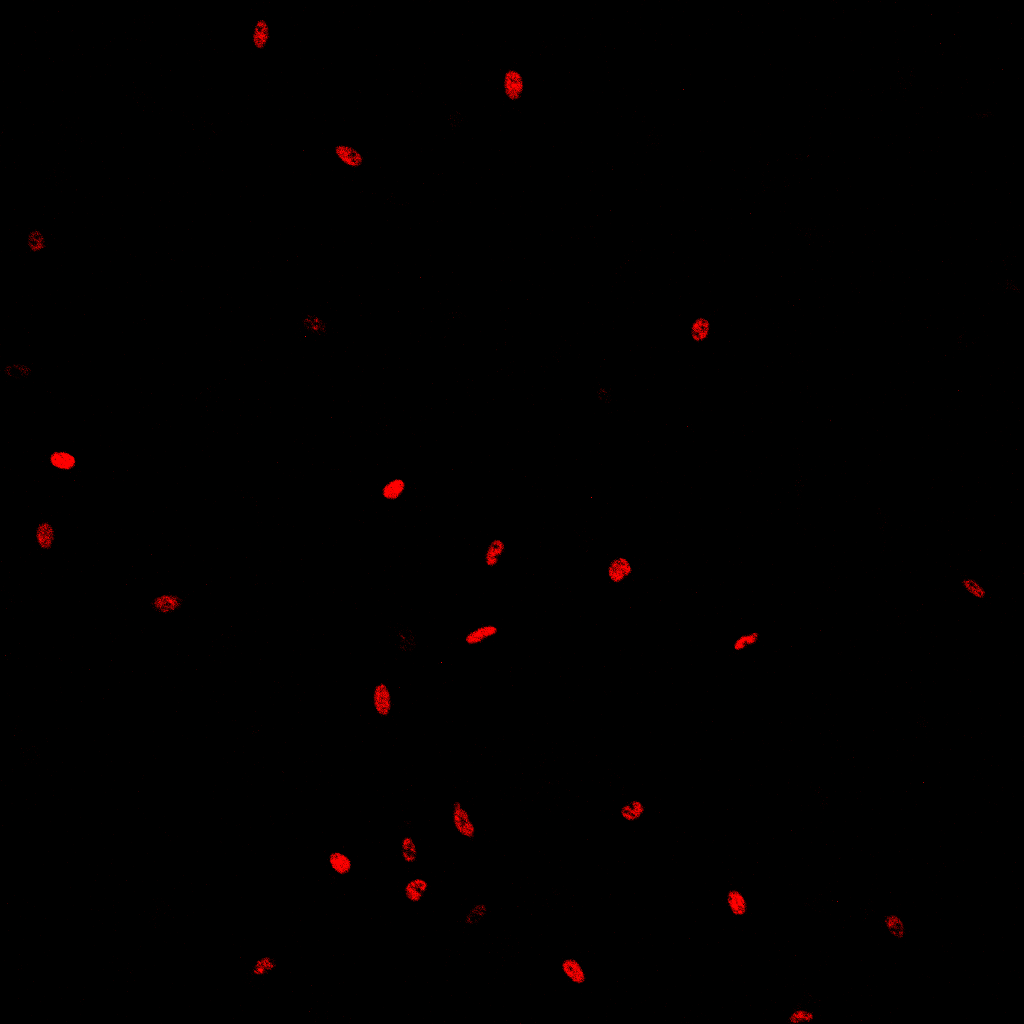

Supplement: Supplementary file 2 [file Presentation1.zip › EDU after drug screening/T39222/2 Edu.tif]

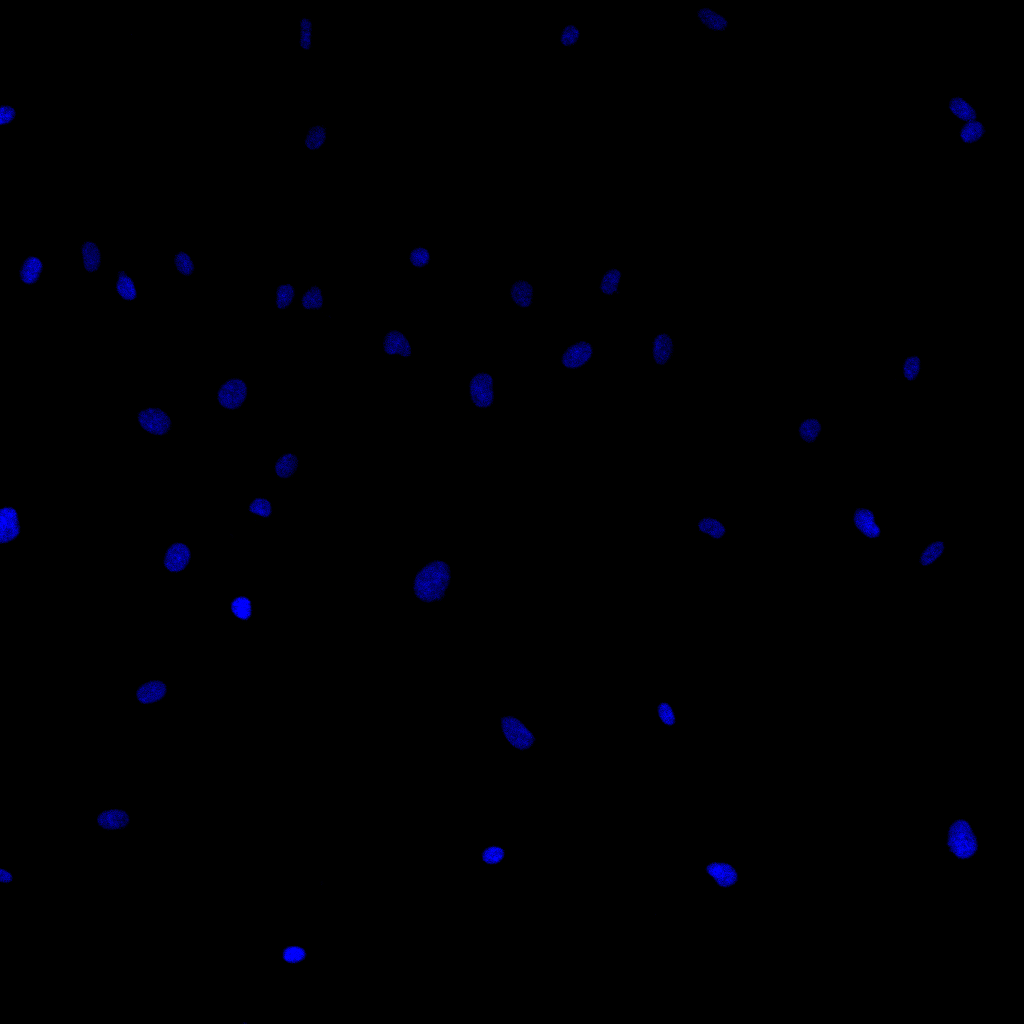

Supplement: Supplementary file 2 [file Presentation1.zip › EDU after drug screening/T39222/3 DAPI.tif]

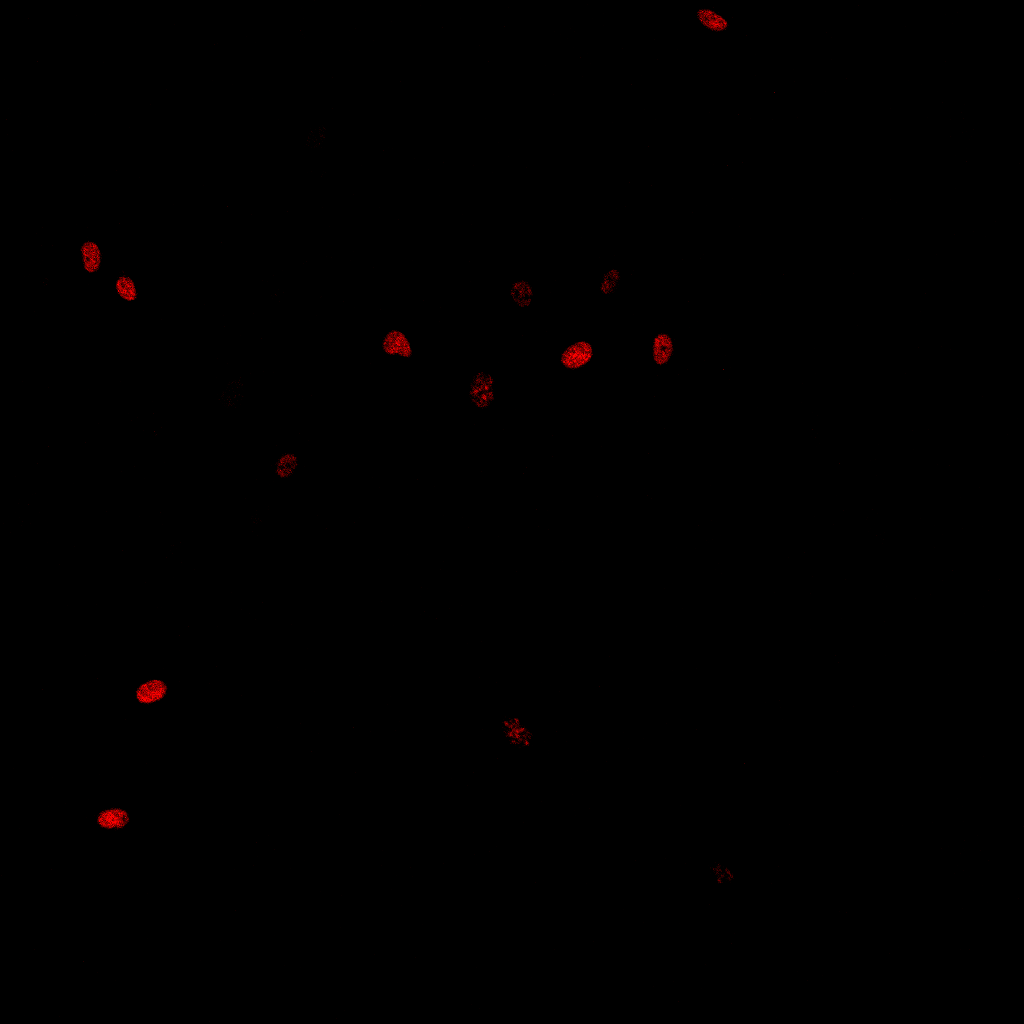

Supplement: Supplementary file 2 [file Presentation1.zip › EDU after drug screening/T39222/3 Edu.tif]

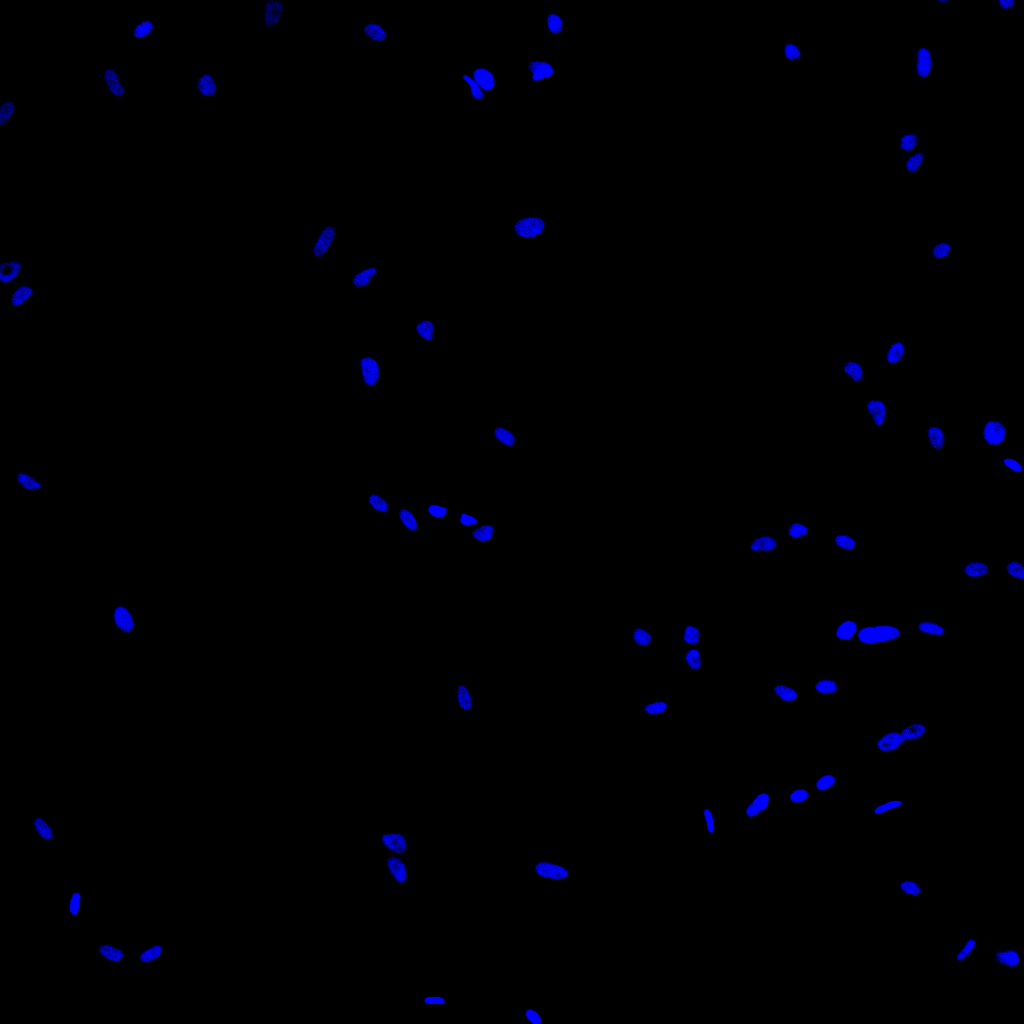

Supplement: Supplementary file 2 [file Presentation1.zip › EDU after drug screening/T4457/1 DAPI.tif]

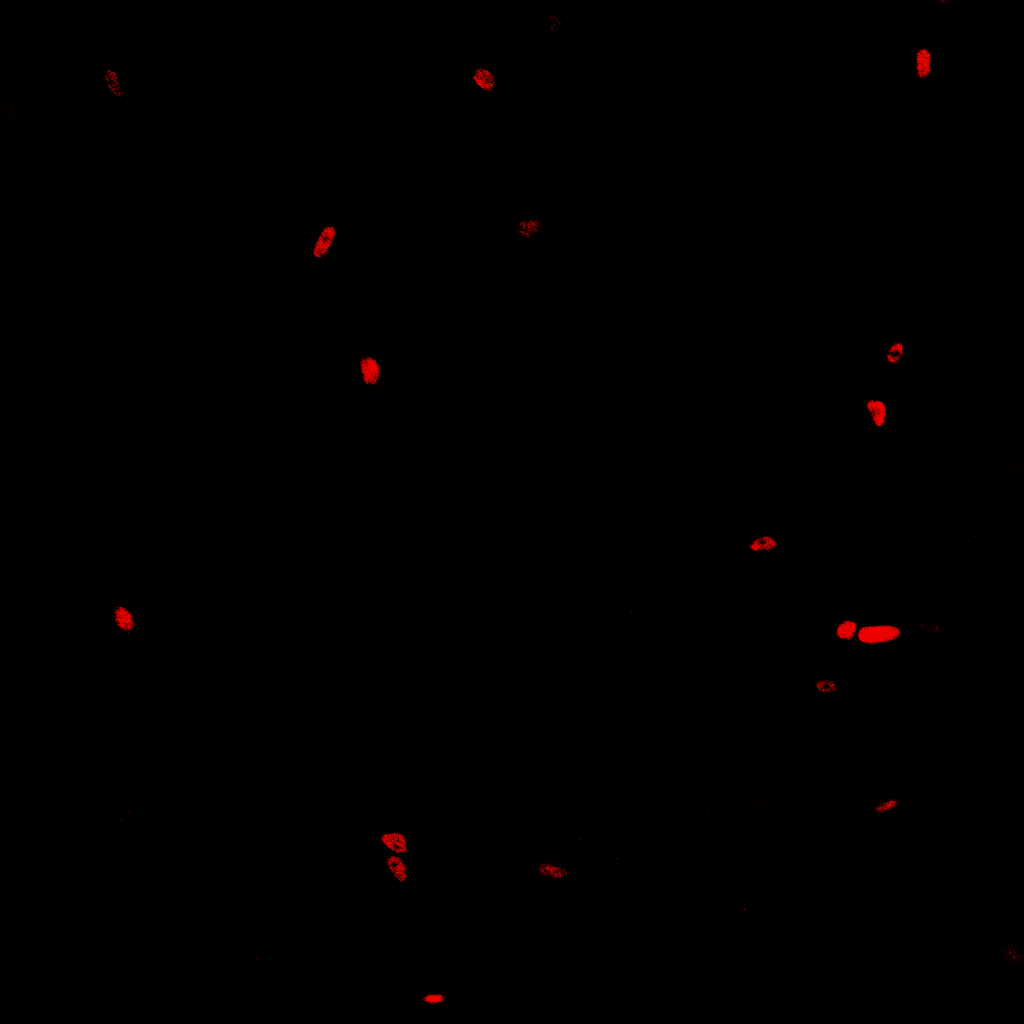

Supplement: Supplementary file 2 [file Presentation1.zip › EDU after drug screening/T4457/1 Edu.tif]

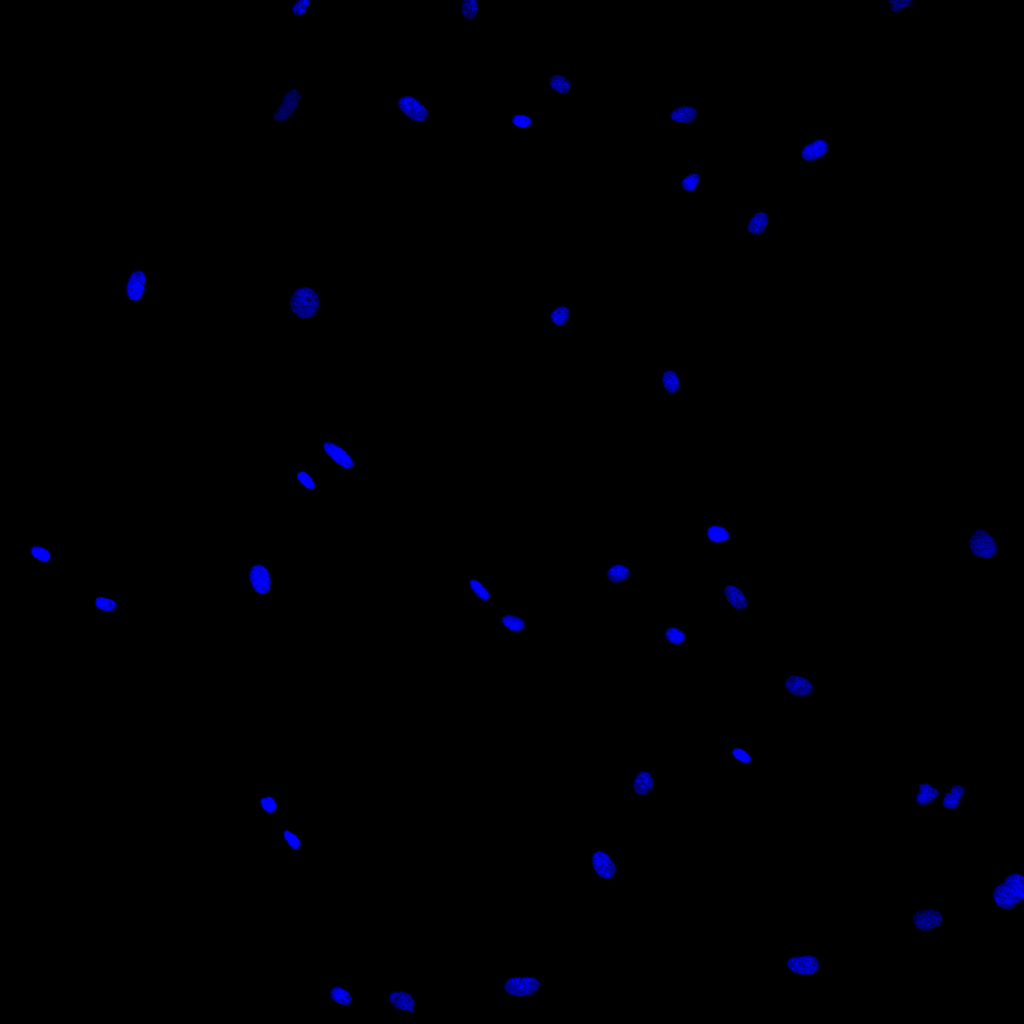

Supplement: Supplementary file 2 [file Presentation1.zip › EDU after drug screening/T4457/2 DAPI.tif]

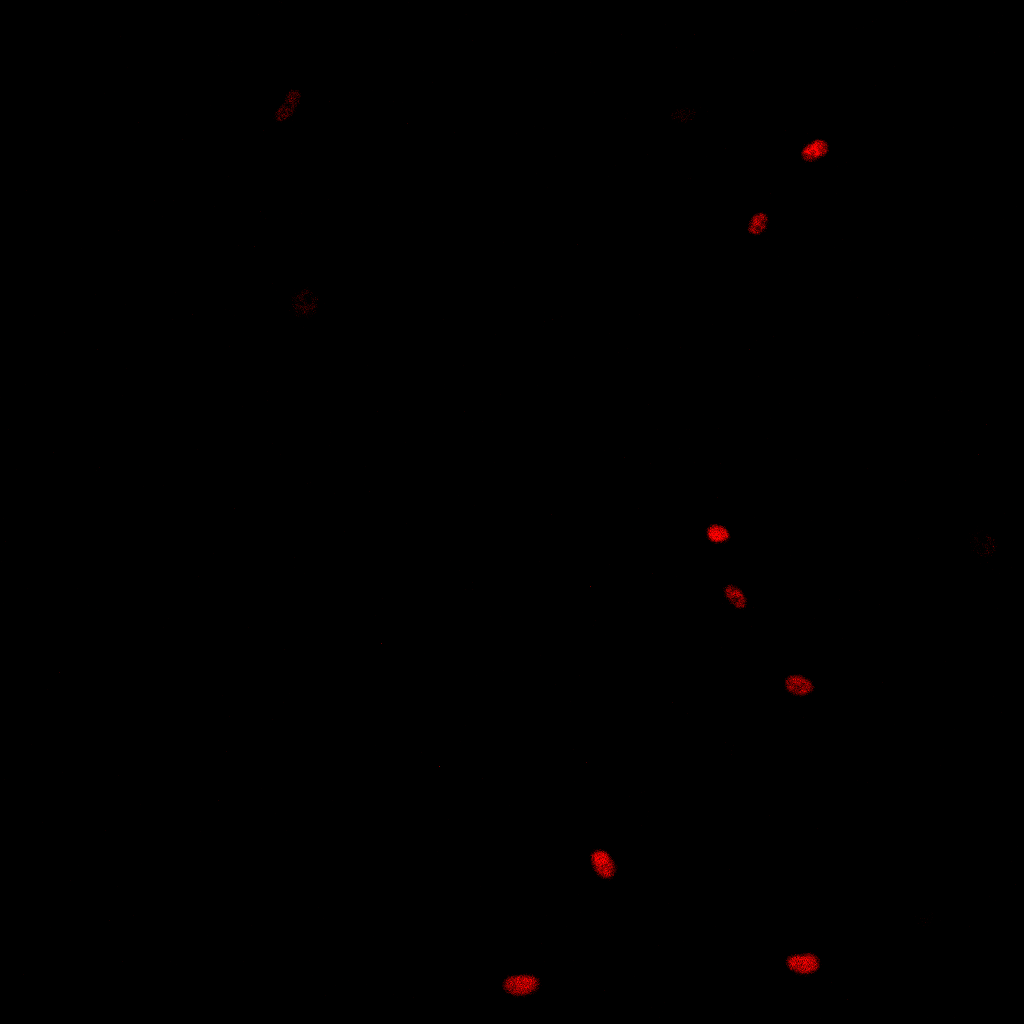

Supplement: Supplementary file 2 [file Presentation1.zip › EDU after drug screening/T4457/2 Edu.tif]

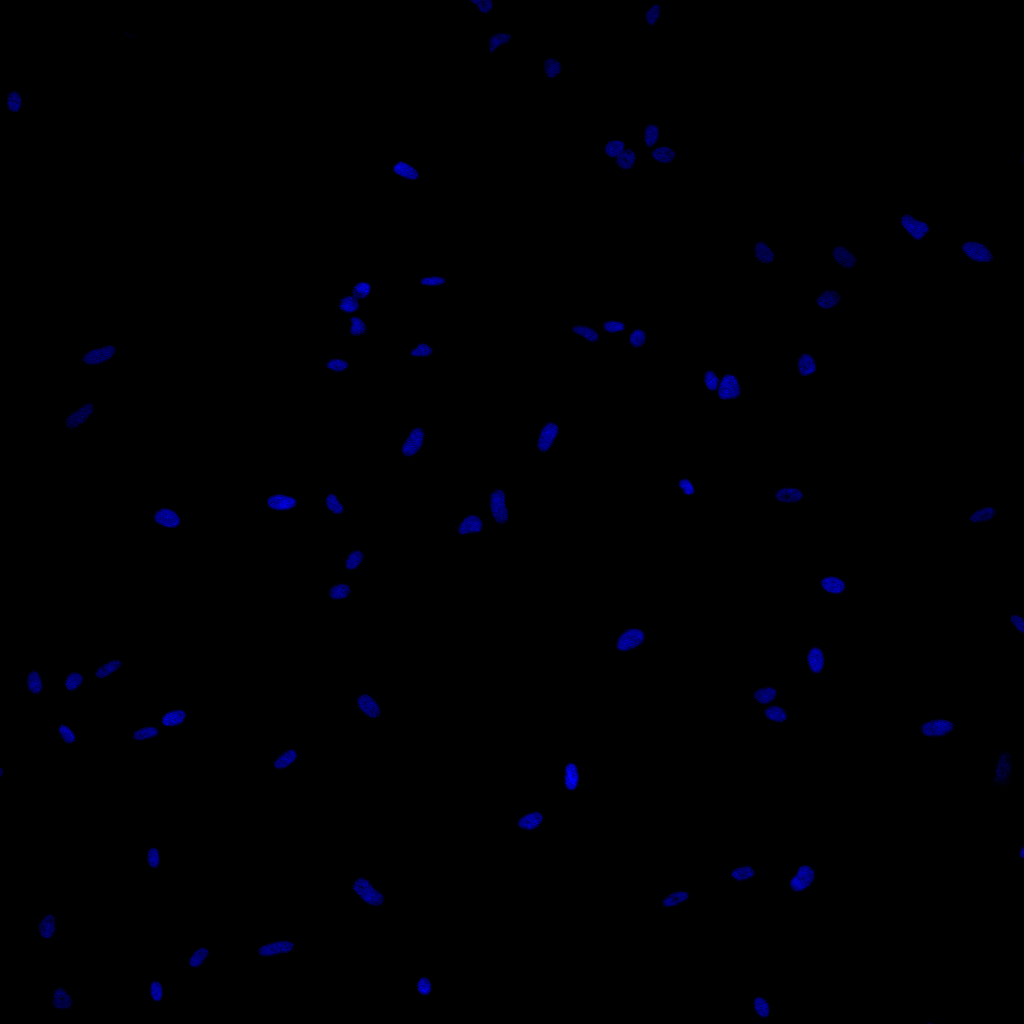

Supplement: Supplementary file 2 [file Presentation1.zip › EDU after drug screening/T4457/3 DAPI.tif]

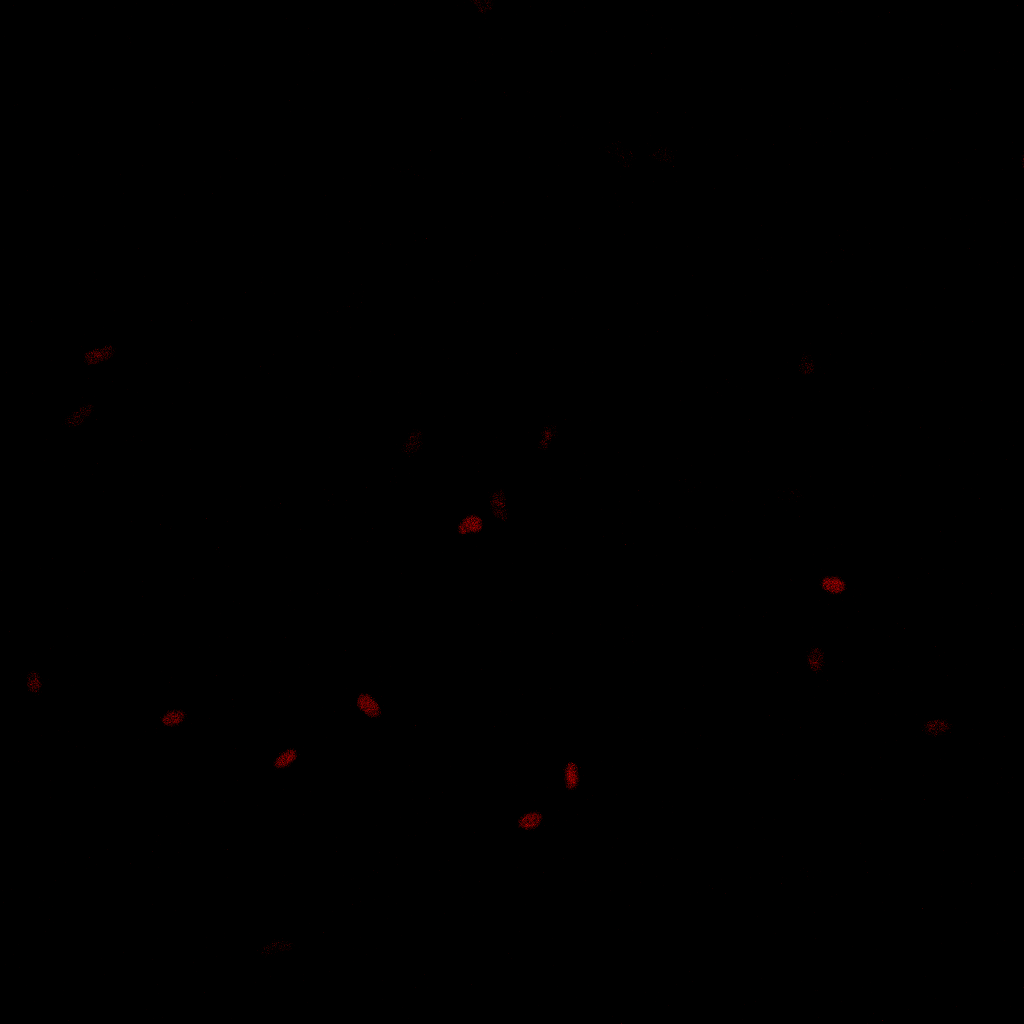

Supplement: Supplementary file 2 [file Presentation1.zip › EDU after drug screening/T4457/3 Edu.tif]

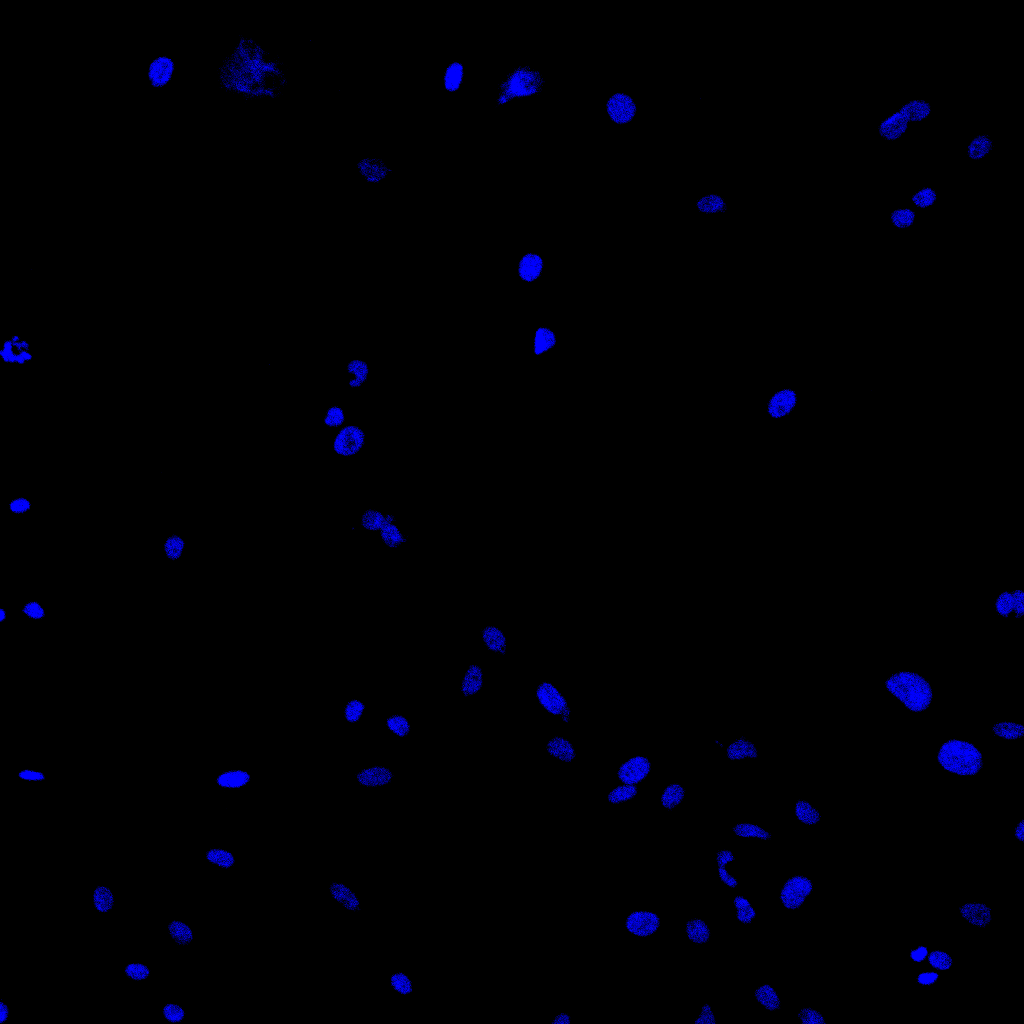

Supplement: Supplementary file 2 [file Presentation1.zip › EDU after drug screening/T5036/1 DAPI.tif]

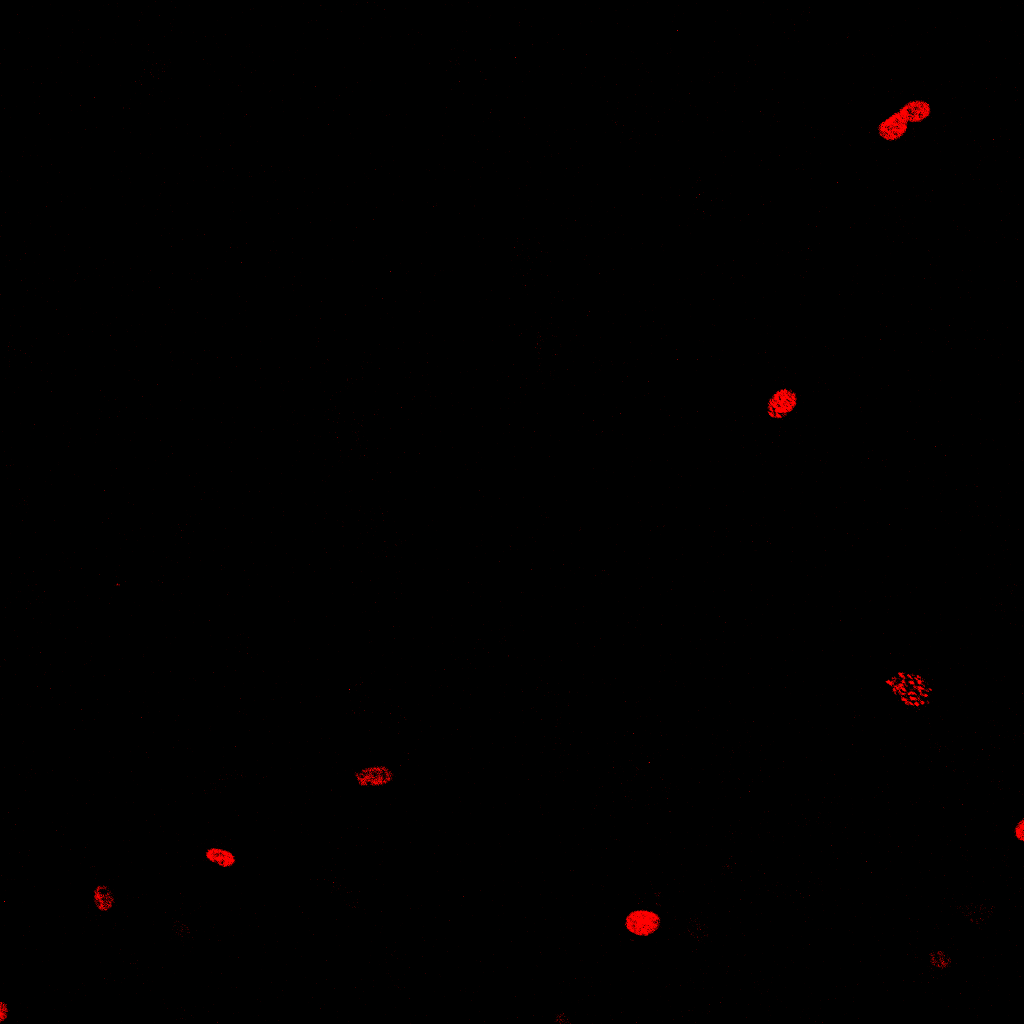

Supplement: Supplementary file 2 [file Presentation1.zip › EDU after drug screening/T5036/1 Edu.tif]

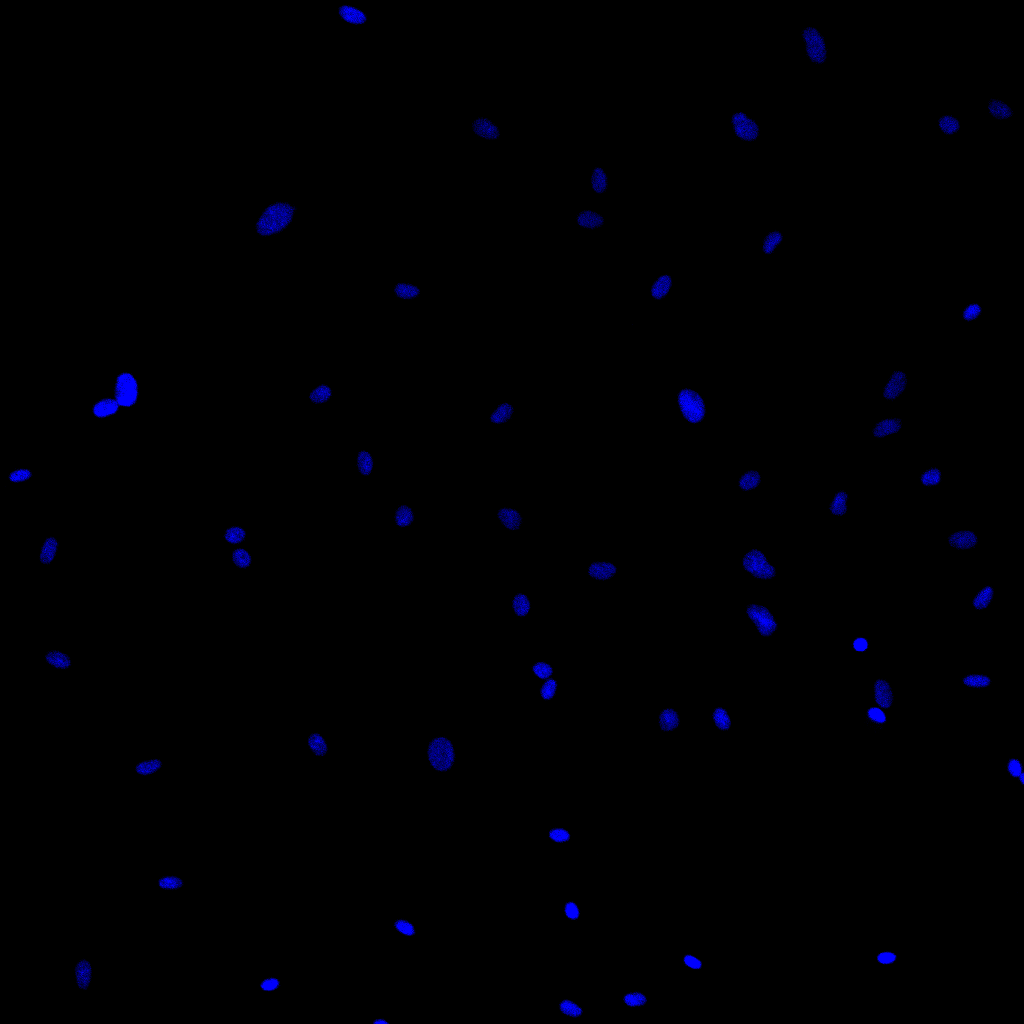

Supplement: Supplementary file 2 [file Presentation1.zip › EDU after drug screening/T5036/2 DAPI.tif]

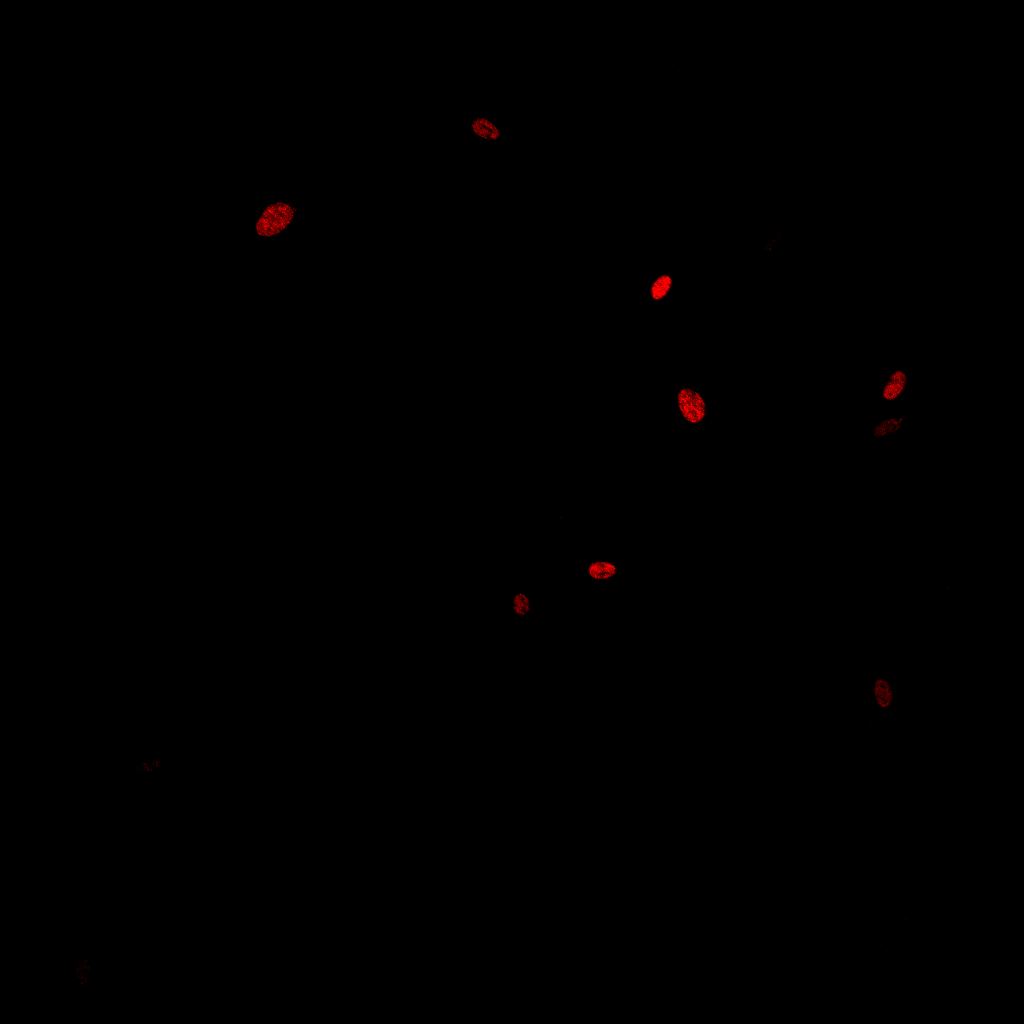

Supplement: Supplementary file 2 [file Presentation1.zip › EDU after drug screening/T5036/2 Edu.tif]

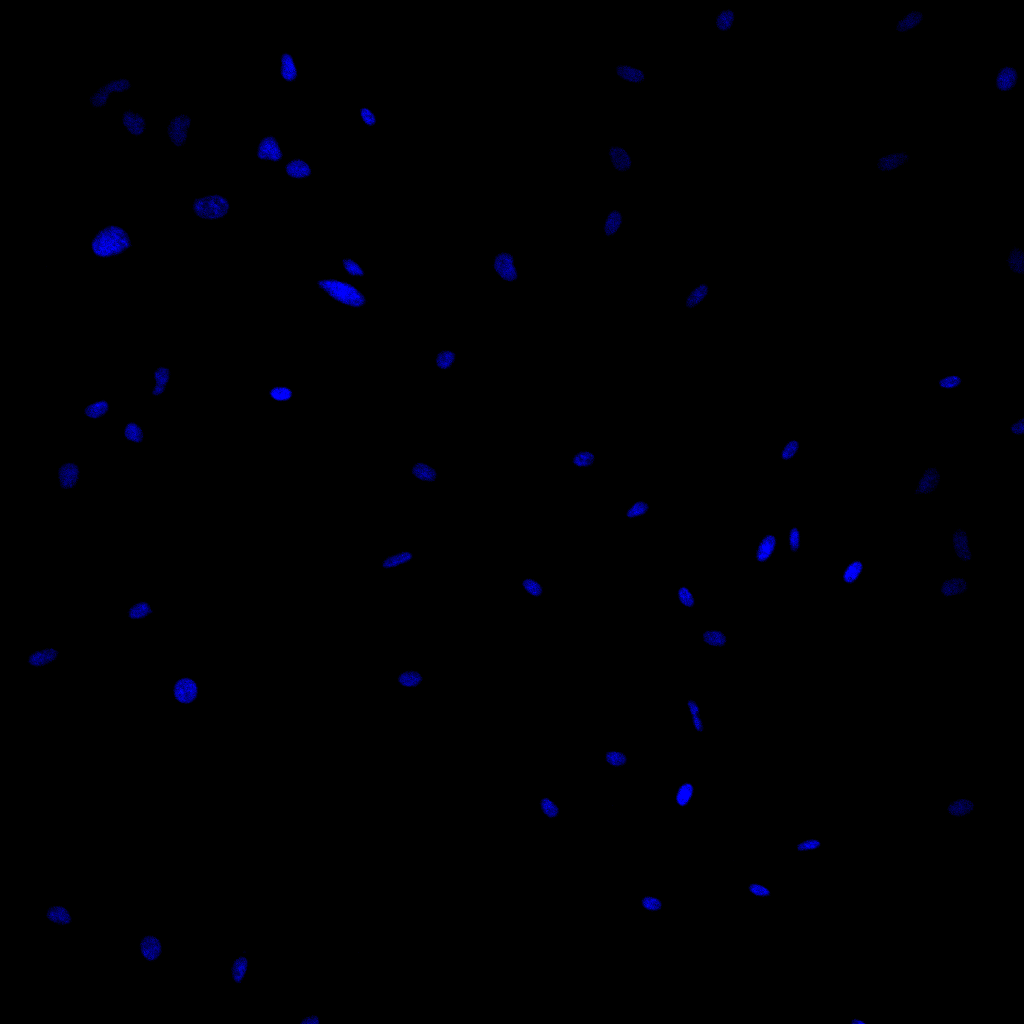

Supplement: Supplementary file 2 [file Presentation1.zip › EDU after drug screening/T5036/3 DAPI.tif]

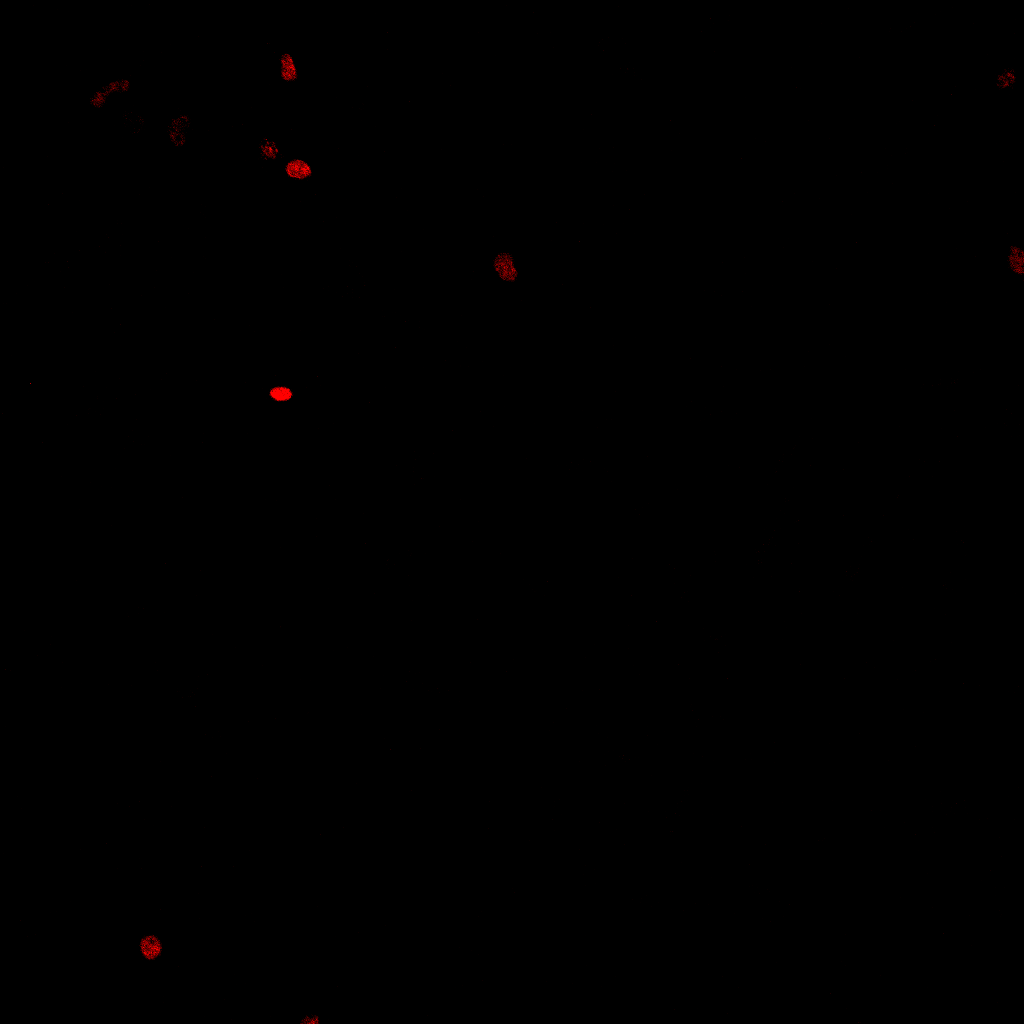

Supplement: Supplementary file 2 [file Presentation1.zip › EDU after drug screening/T5036/3 Edu.tif]

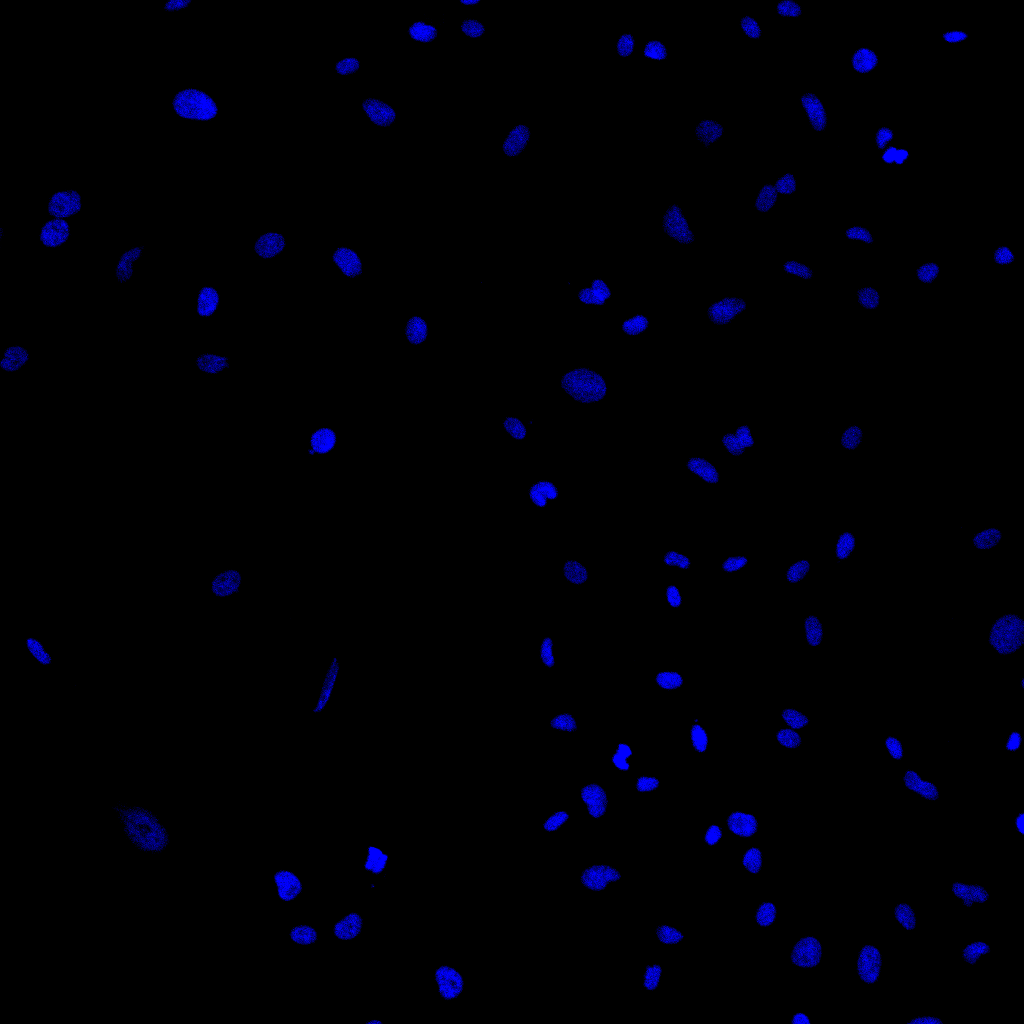

Supplement: Supplementary file 2 [file Presentation1.zip › EDU after drug screening/T7285/1 DAPI.tif]

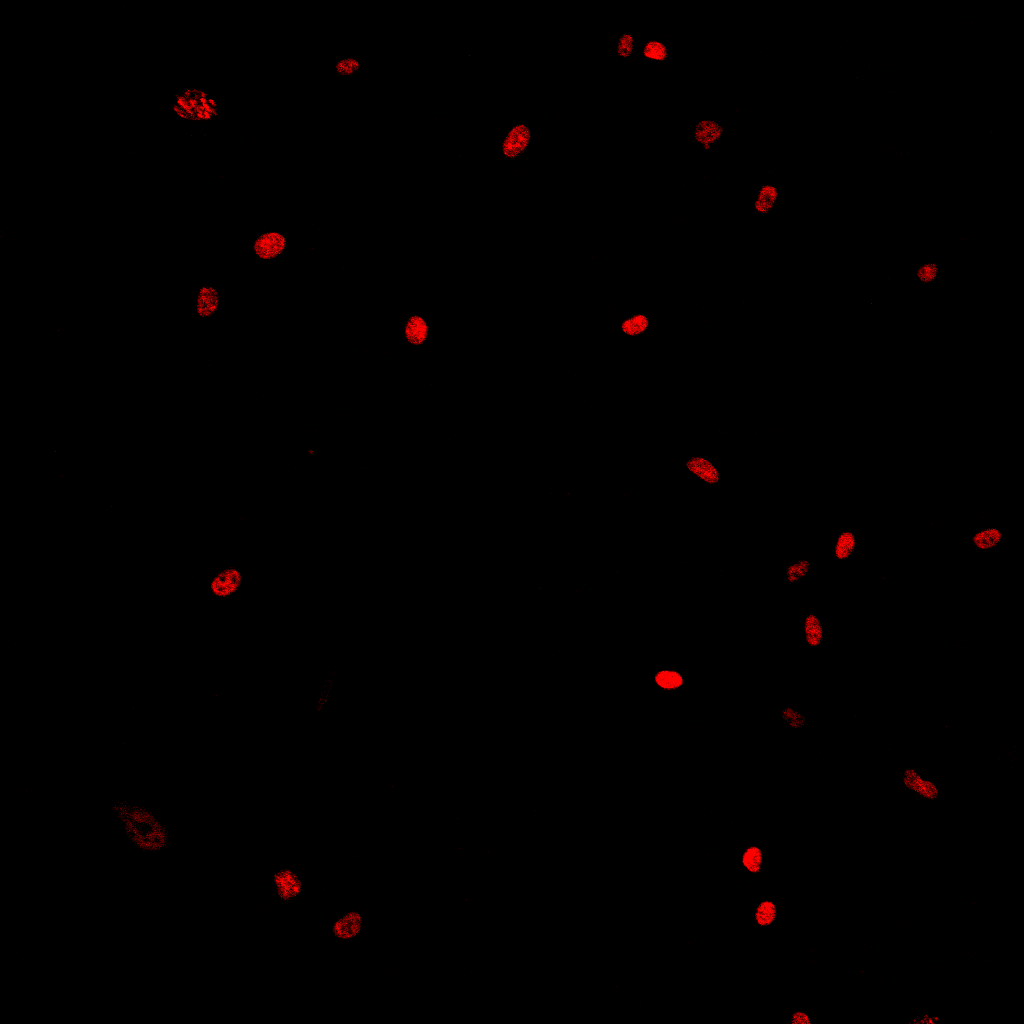

Supplement: Supplementary file 2 [file Presentation1.zip › EDU after drug screening/T7285/1 Edu.tif]

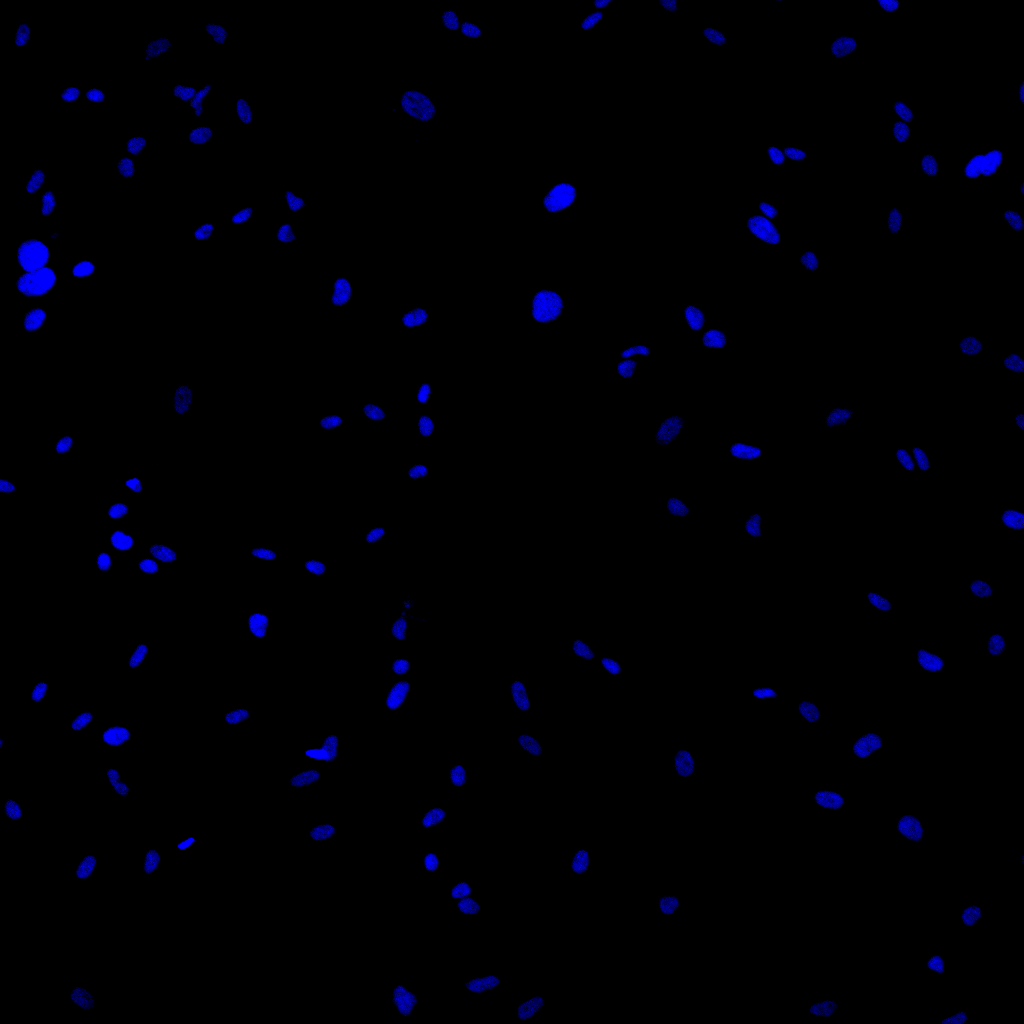

Supplement: Supplementary file 2 [file Presentation1.zip › EDU after drug screening/T7285/2 DAPI.tif]

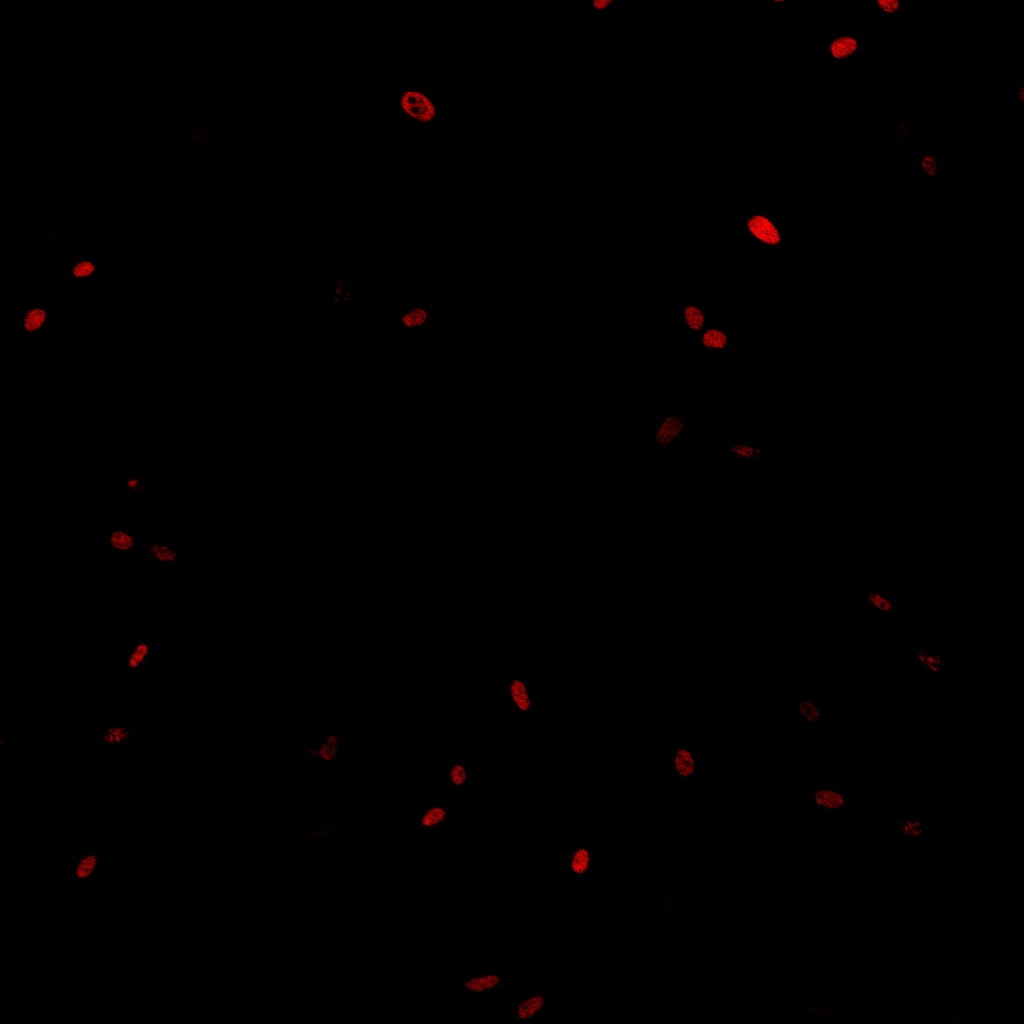

Supplement: Supplementary file 2 [file Presentation1.zip › EDU after drug screening/T7285/2 Edu.tif]

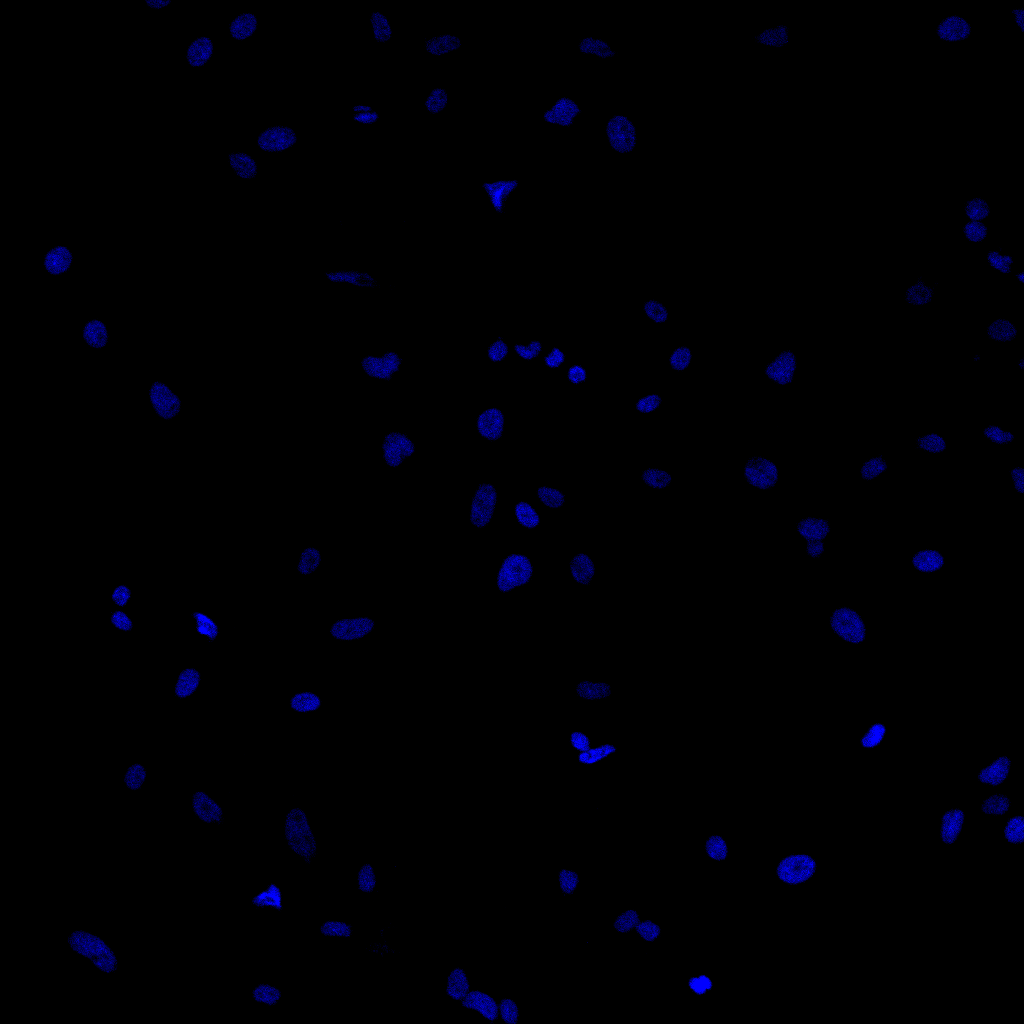

Supplement: Supplementary file 2 [file Presentation1.zip › EDU after drug screening/T7285/3 DAPI.tif]

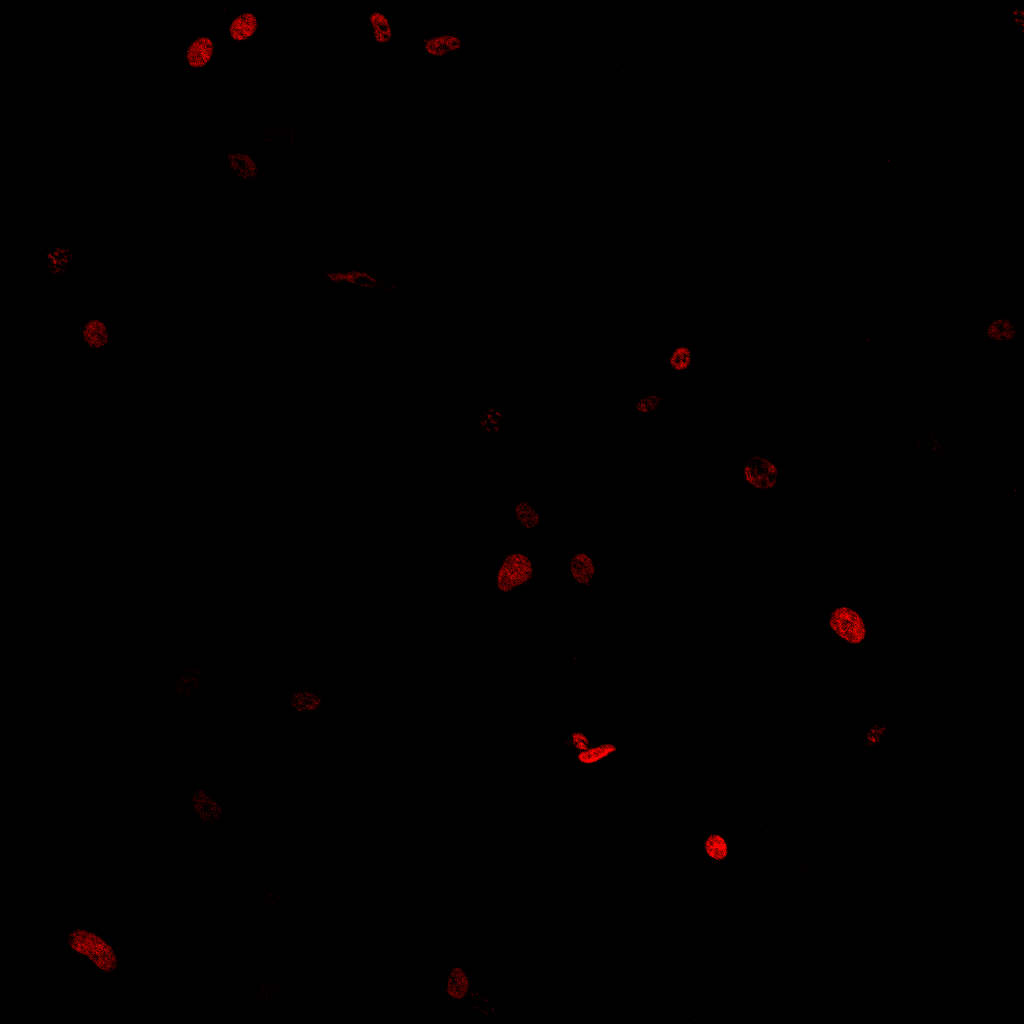

Supplement: Supplementary file 2 [file Presentation1.zip › EDU after drug screening/T7285/3 Edu.tif]

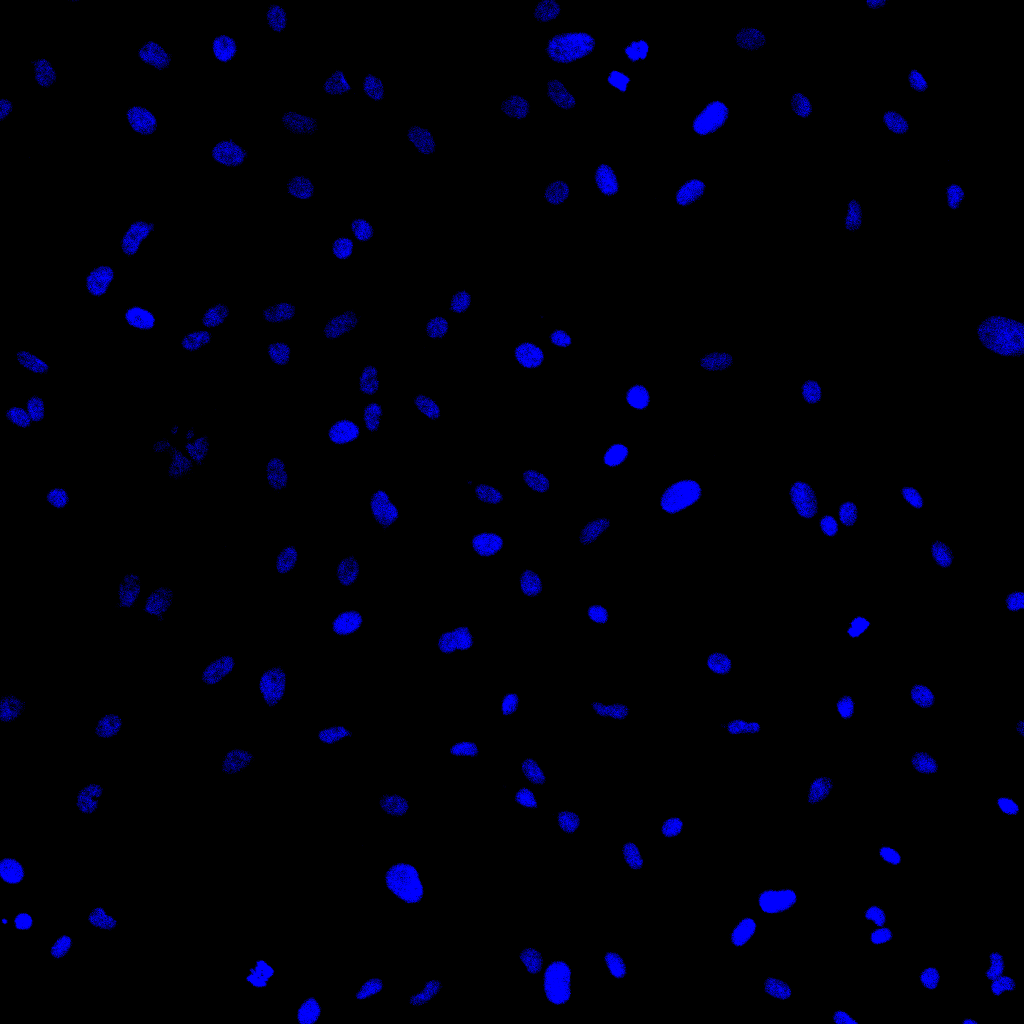

Supplement: Supplementary file 2 [file Presentation1.zip › EDU after drug screening/U87-OE/1 DAPI.tif]

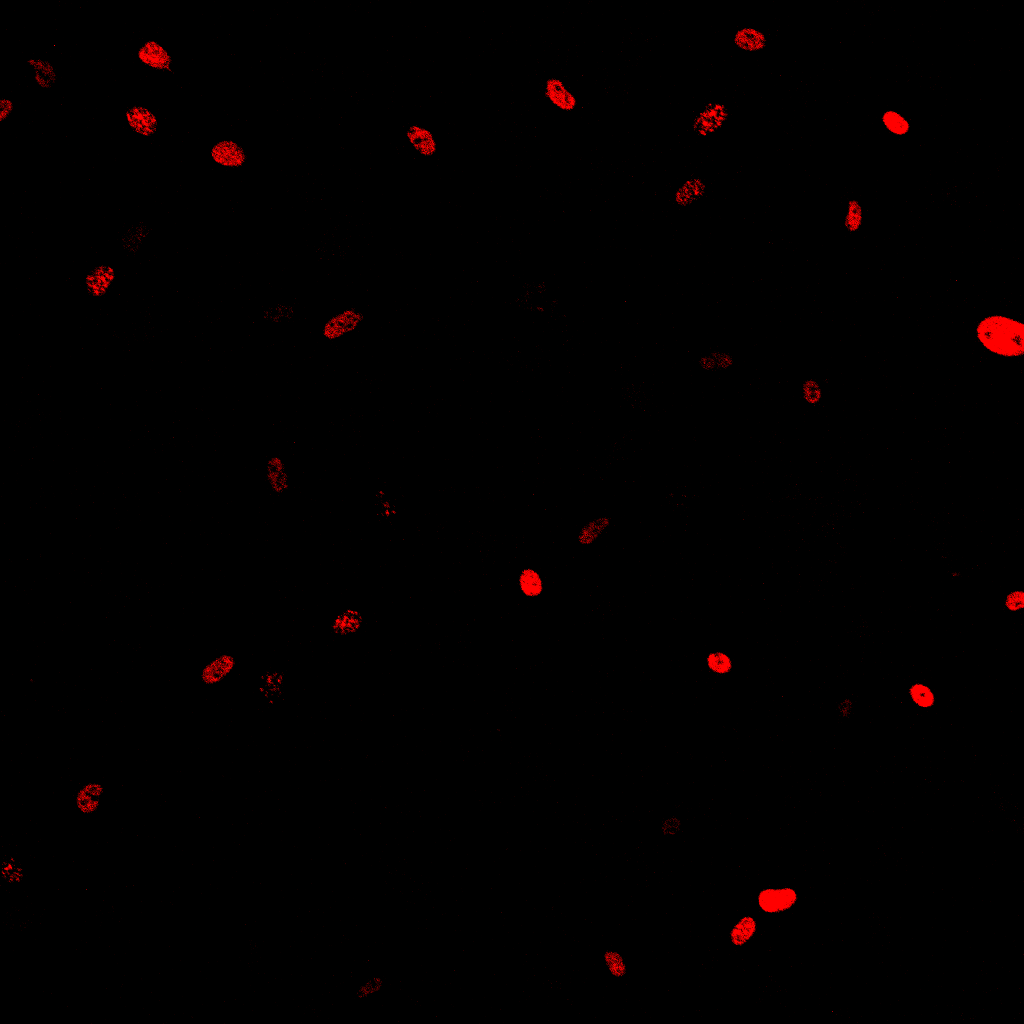

Supplement: Supplementary file 2 [file Presentation1.zip › EDU after drug screening/U87-OE/1 Edu.tif]

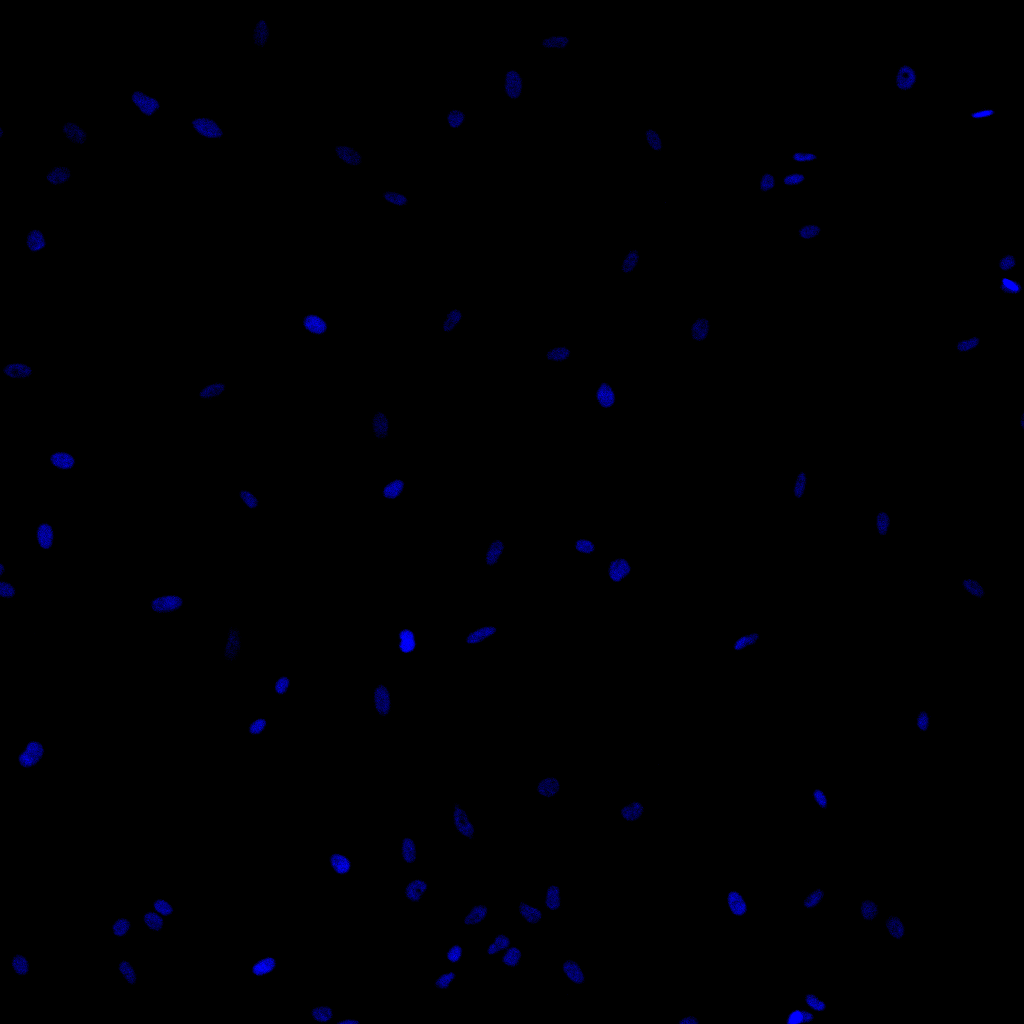

Supplement: Supplementary file 2 [file Presentation1.zip › EDU after drug screening/U87-OE/2 DAPI.tif]

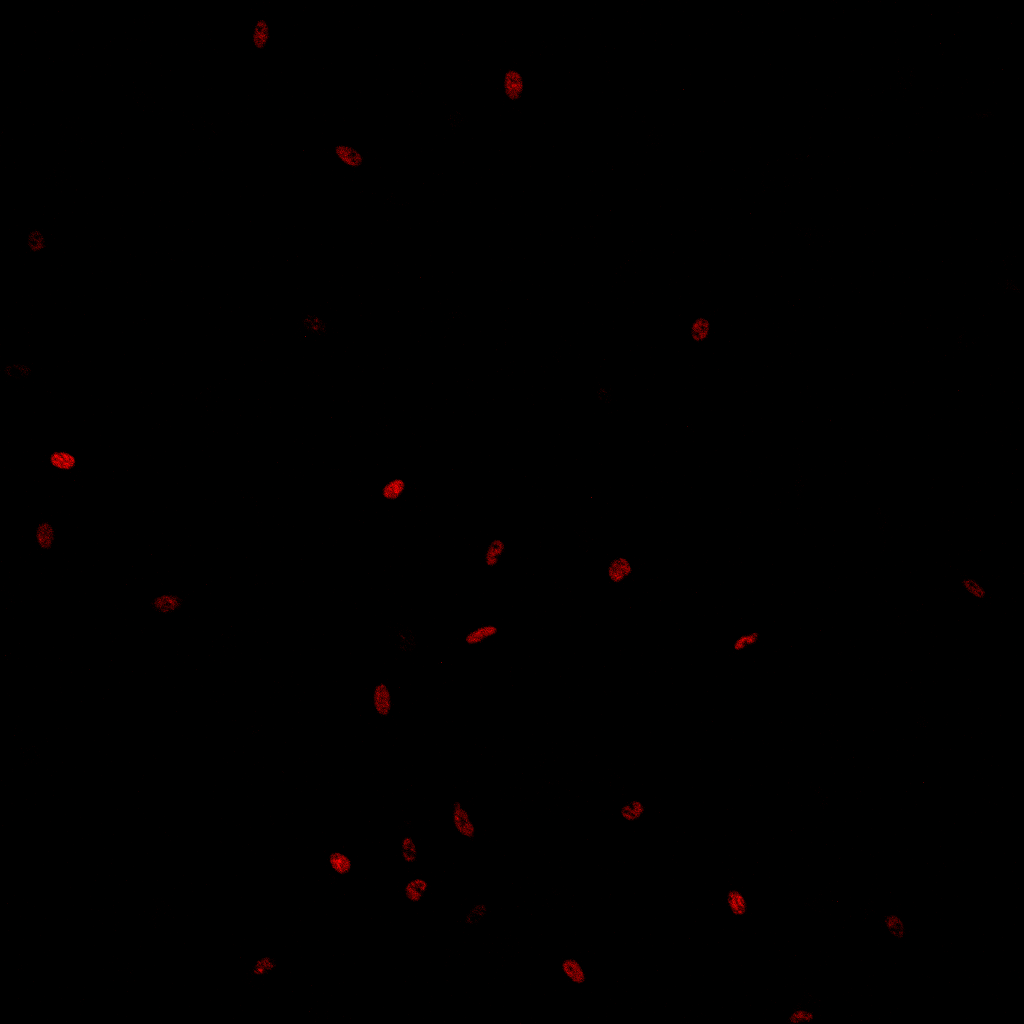

Supplement: Supplementary file 2 [file Presentation1.zip › EDU after drug screening/U87-OE/2 Edu.tif]

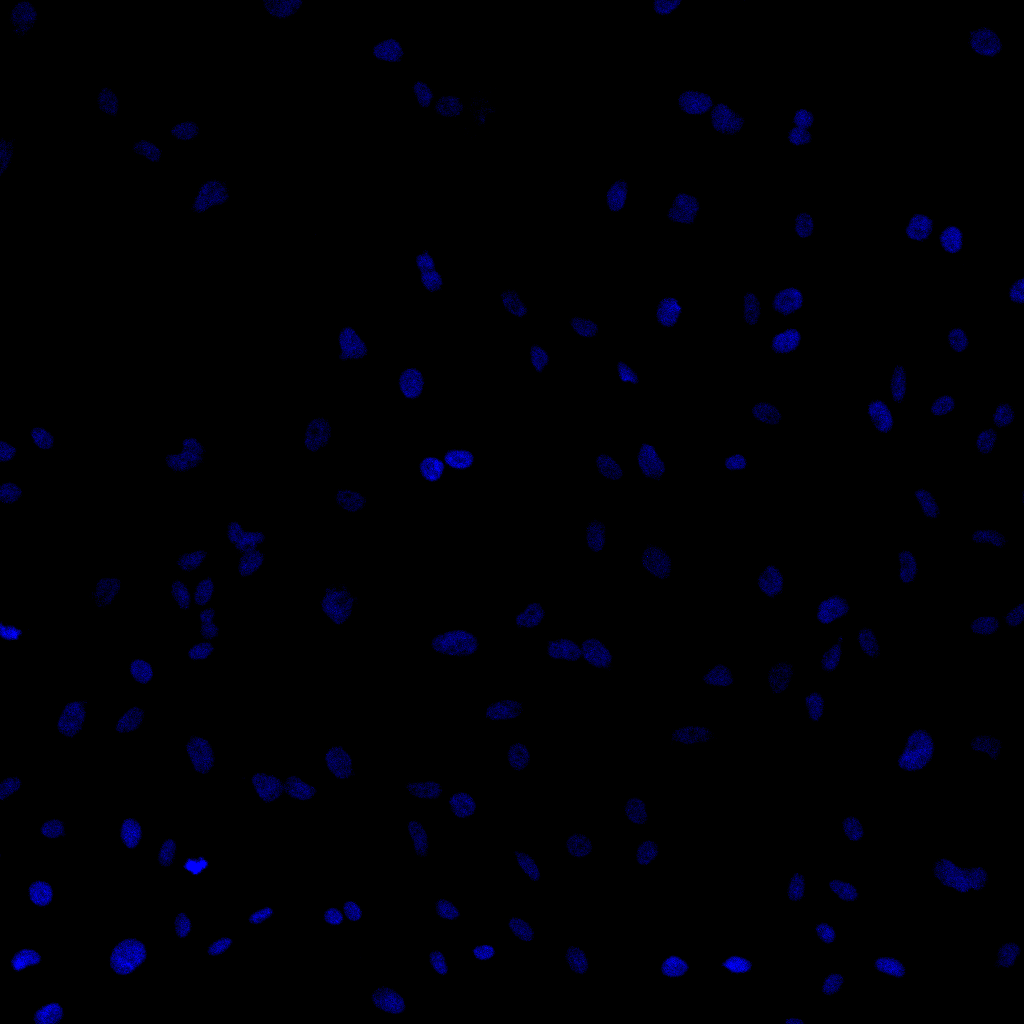

Supplement: Supplementary file 2 [file Presentation1.zip › EDU after drug screening/U87-sham/2 DAPI.tif]

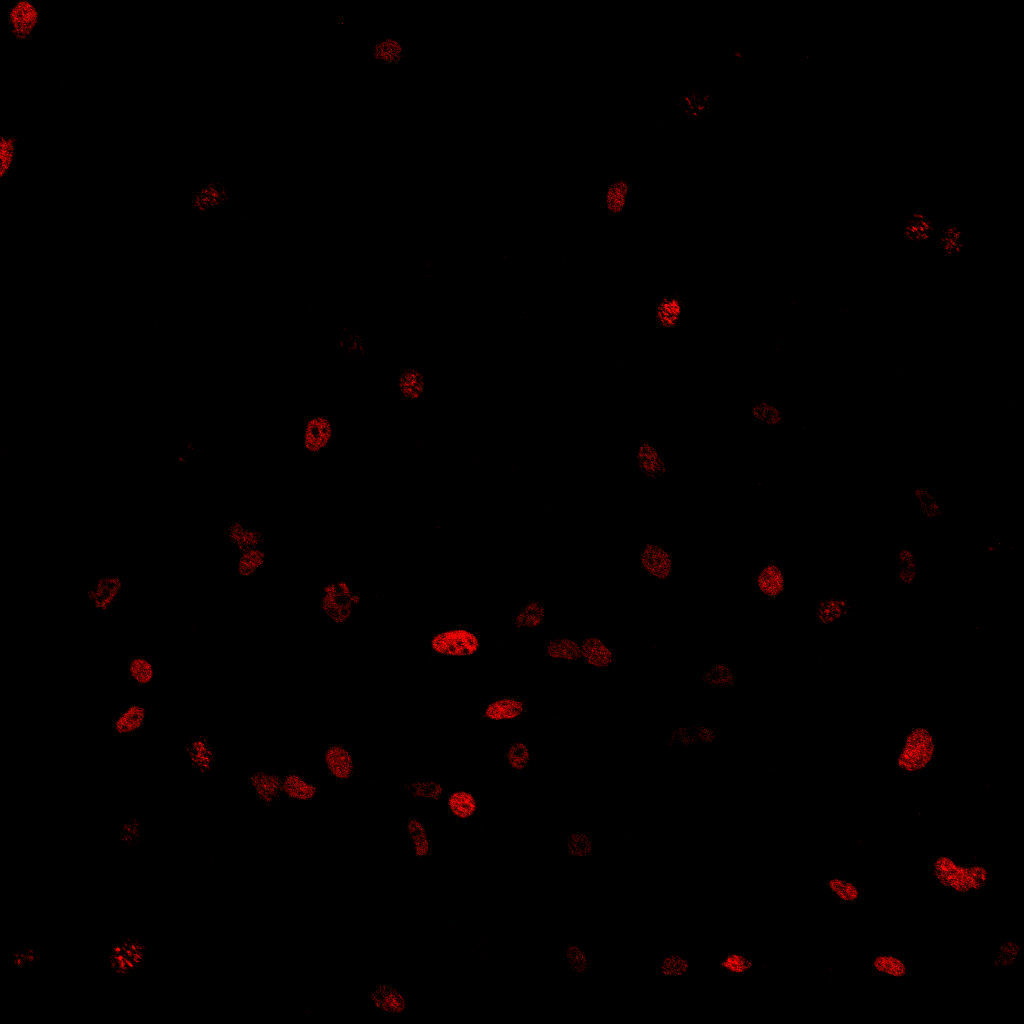

Supplement: Supplementary file 2 [file Presentation1.zip › EDU after drug screening/U87-sham/2 Edu.tif]

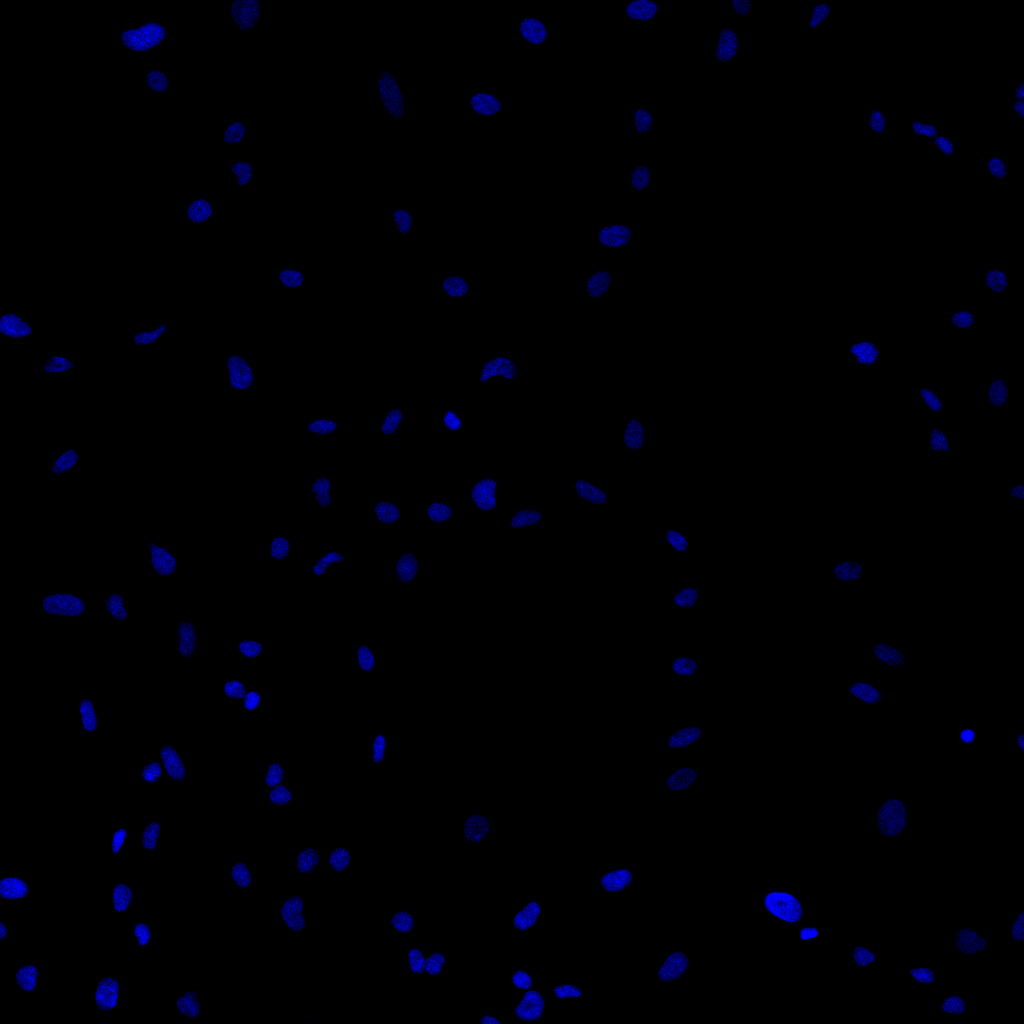

Supplement: Supplementary file 2 [file Presentation1.zip › EDU after drug screening/U87-sham/3 DAPI.tif]

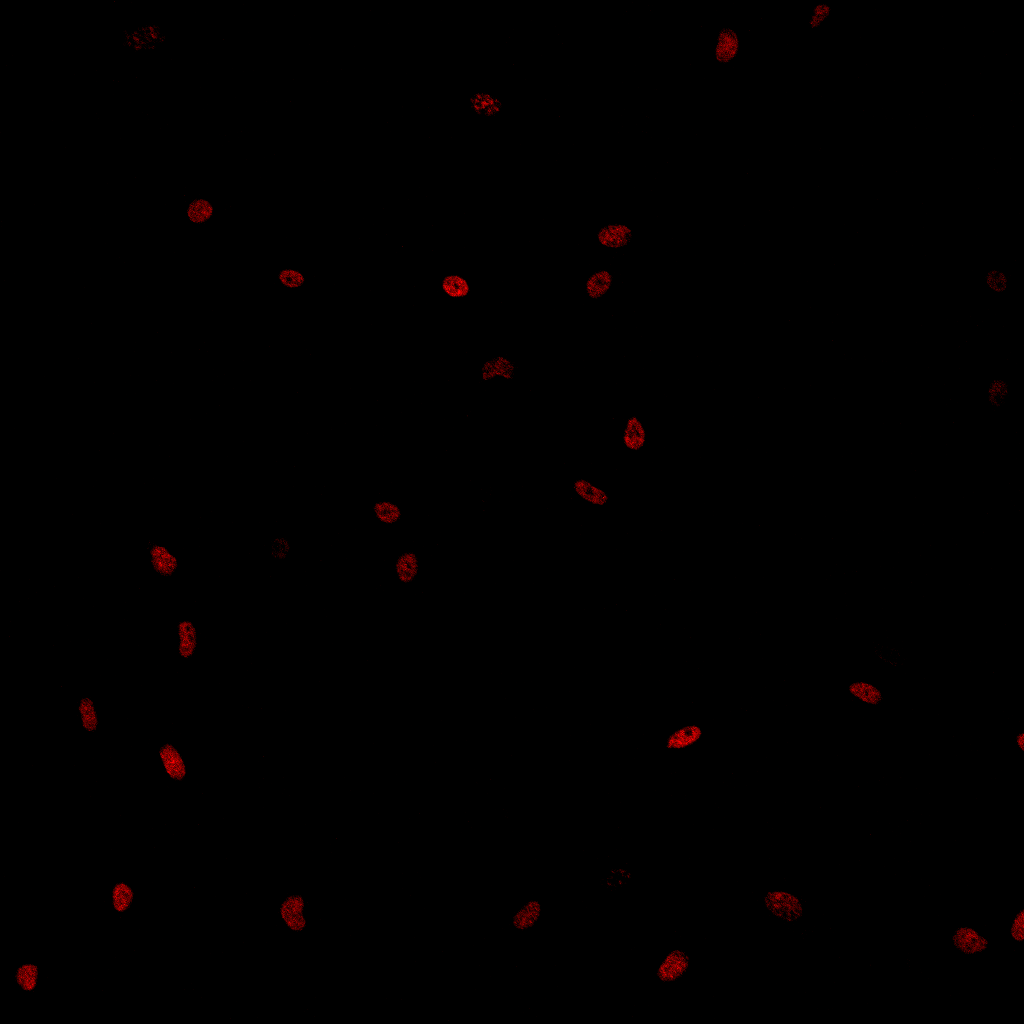

Supplement: Supplementary file 2 [file Presentation1.zip › EDU after drug screening/U87-sham/3 Edu.tif]

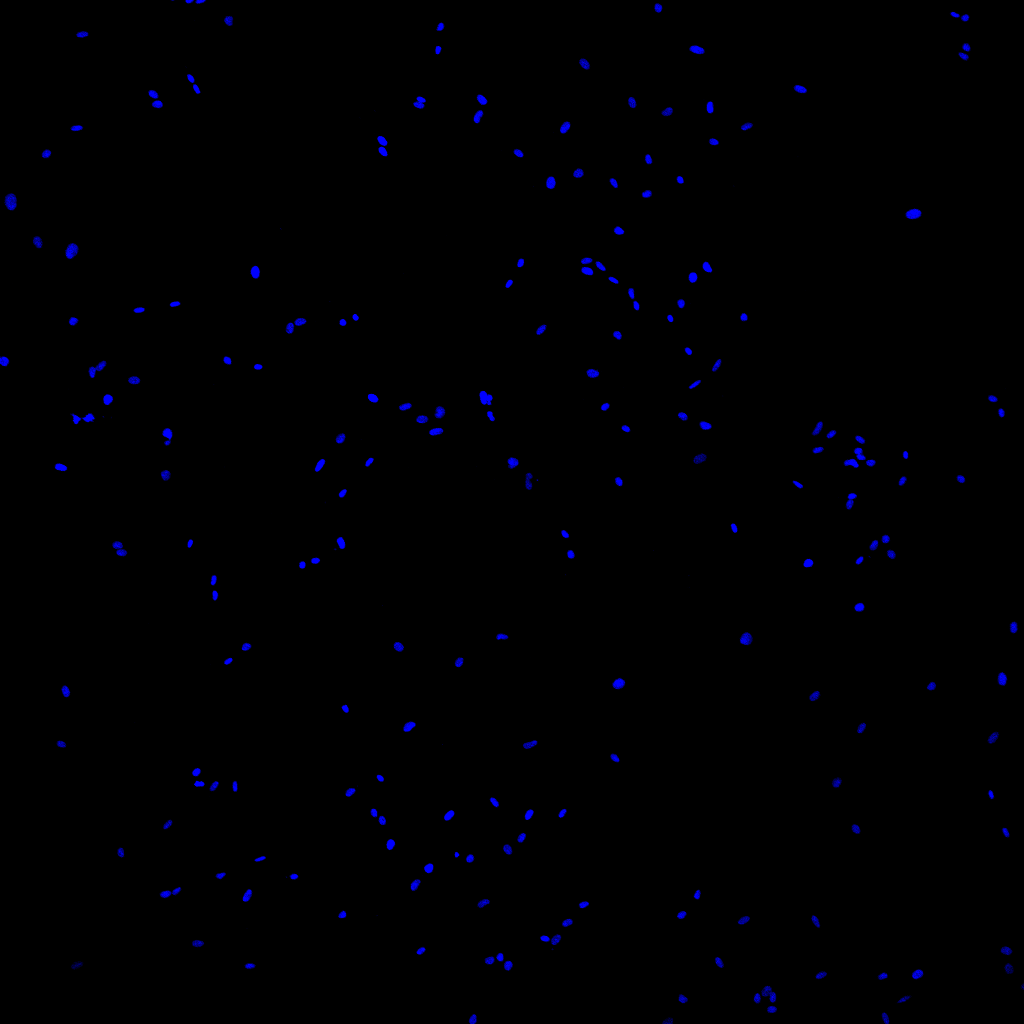

Supplement: Supplementary file 2 [file Presentation1.zip › EDU/U87-PLCB4/1 dapi.tif]

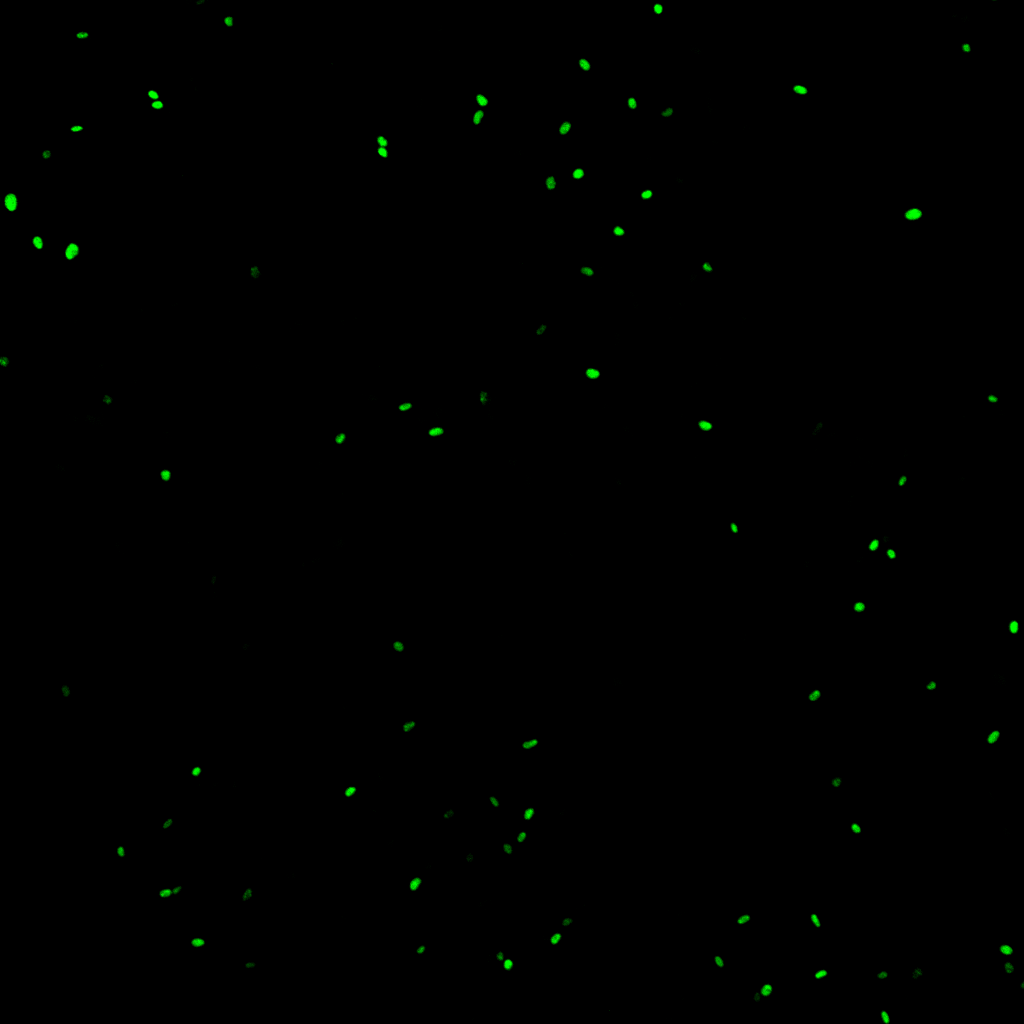

Supplement: Supplementary file 2 [file Presentation1.zip › EDU/U87-PLCB4/1 edu.tif]

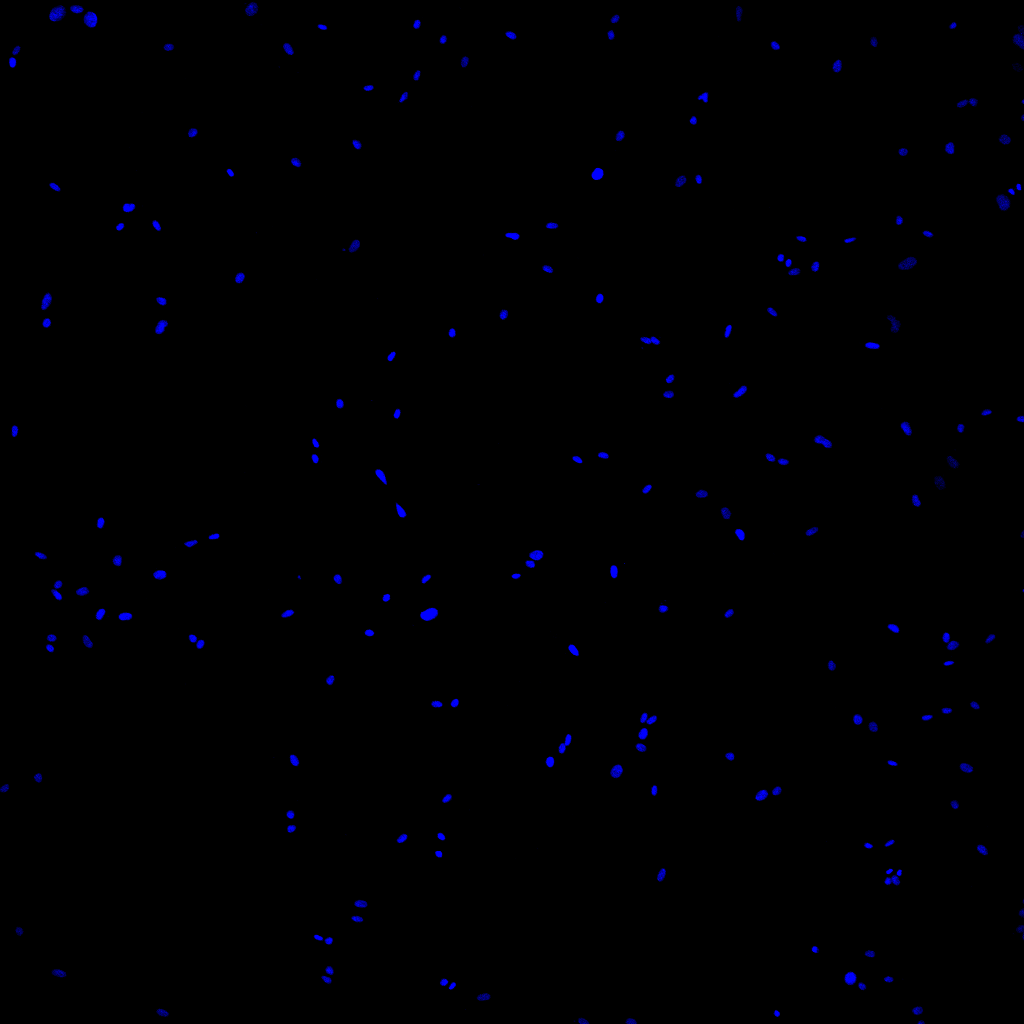

Supplement: Supplementary file 2 [file Presentation1.zip › EDU/U87-PLCB4/2 dapi.tif]

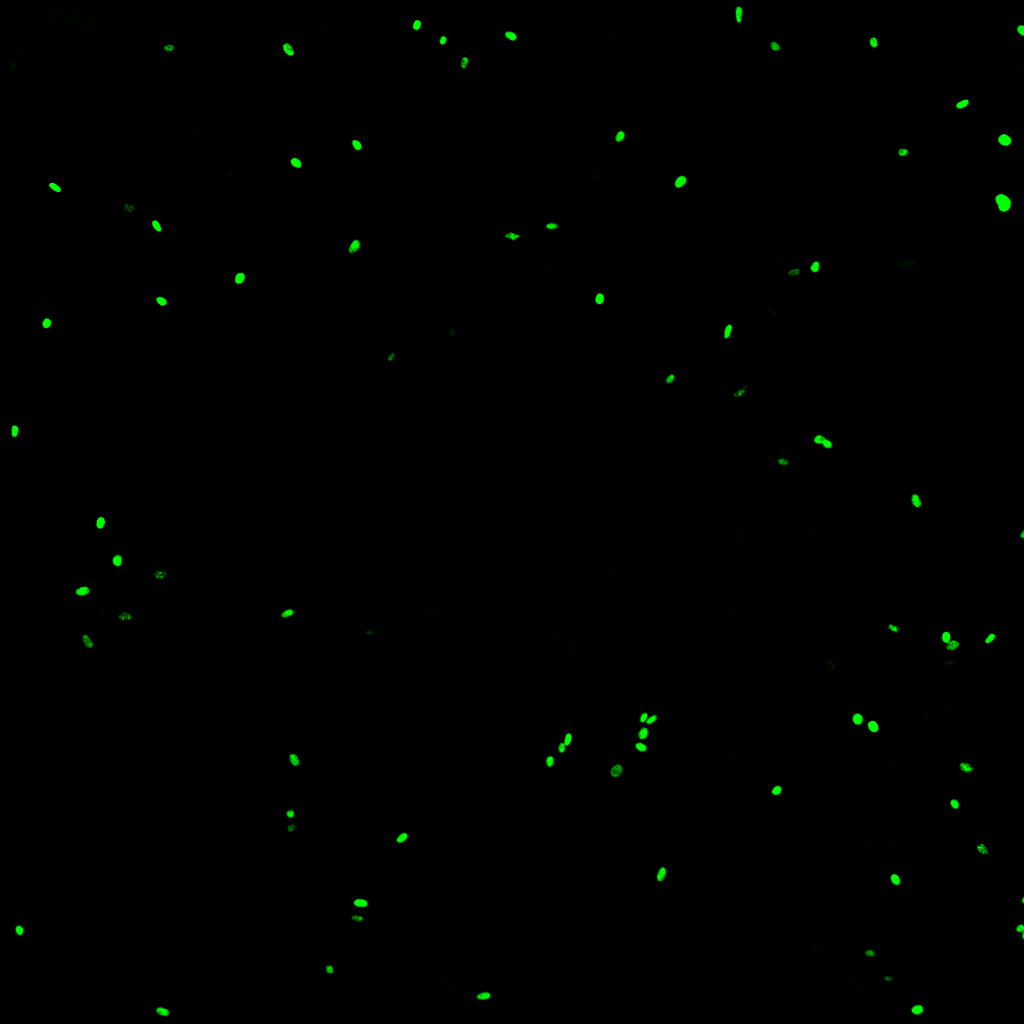

Supplement: Supplementary file 2 [file Presentation1.zip › EDU/U87-PLCB4/2 edu.tif]

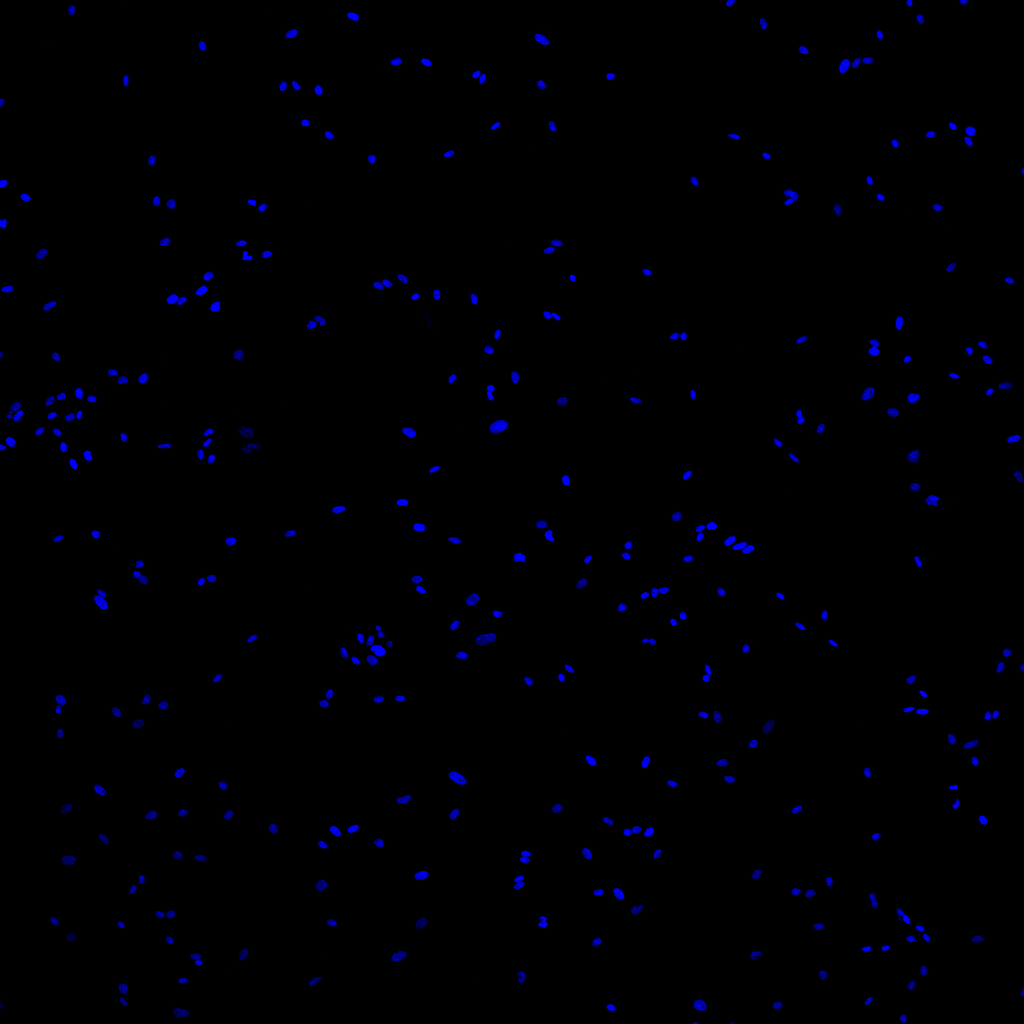

Supplement: Supplementary file 2 [file Presentation1.zip › EDU/U87-PLCB4/3 dapi.tif]

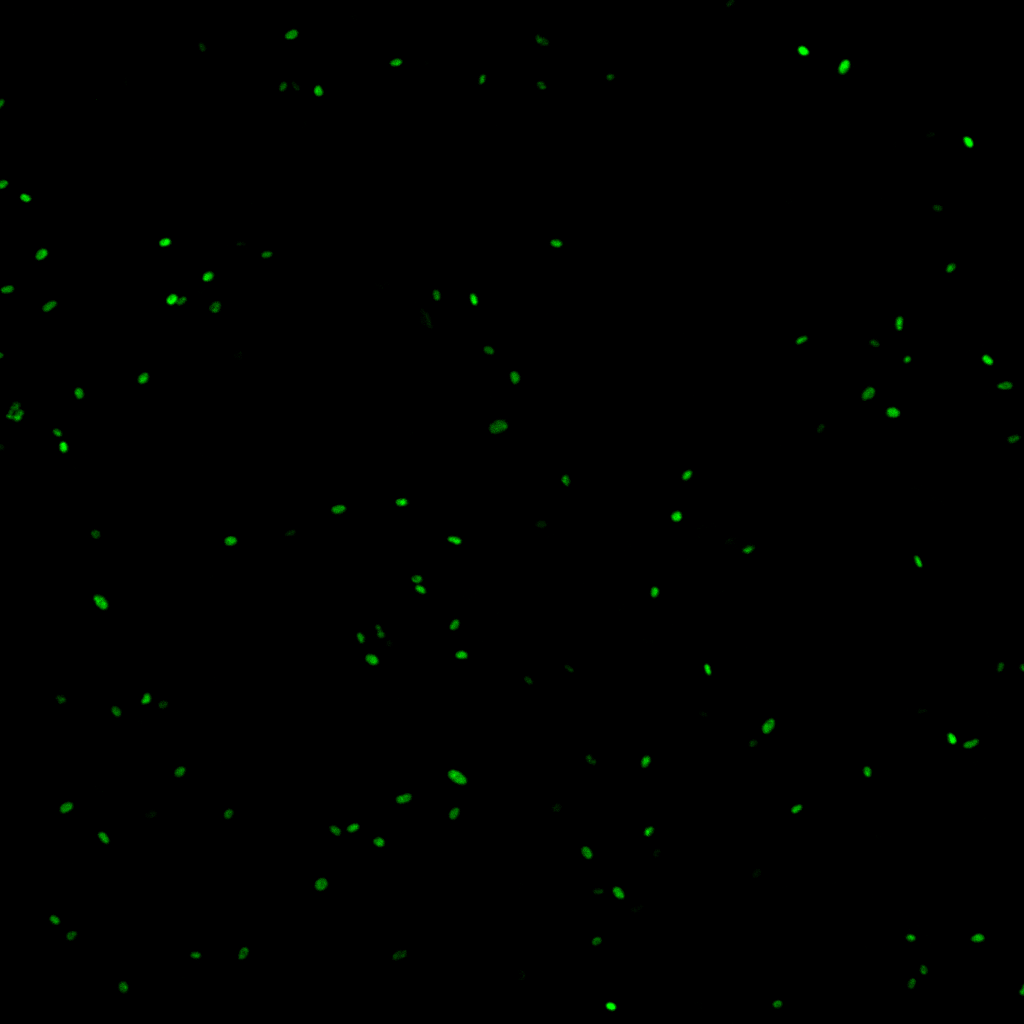

Supplement: Supplementary file 2 [file Presentation1.zip › EDU/U87-PLCB4/3 edu.tif]

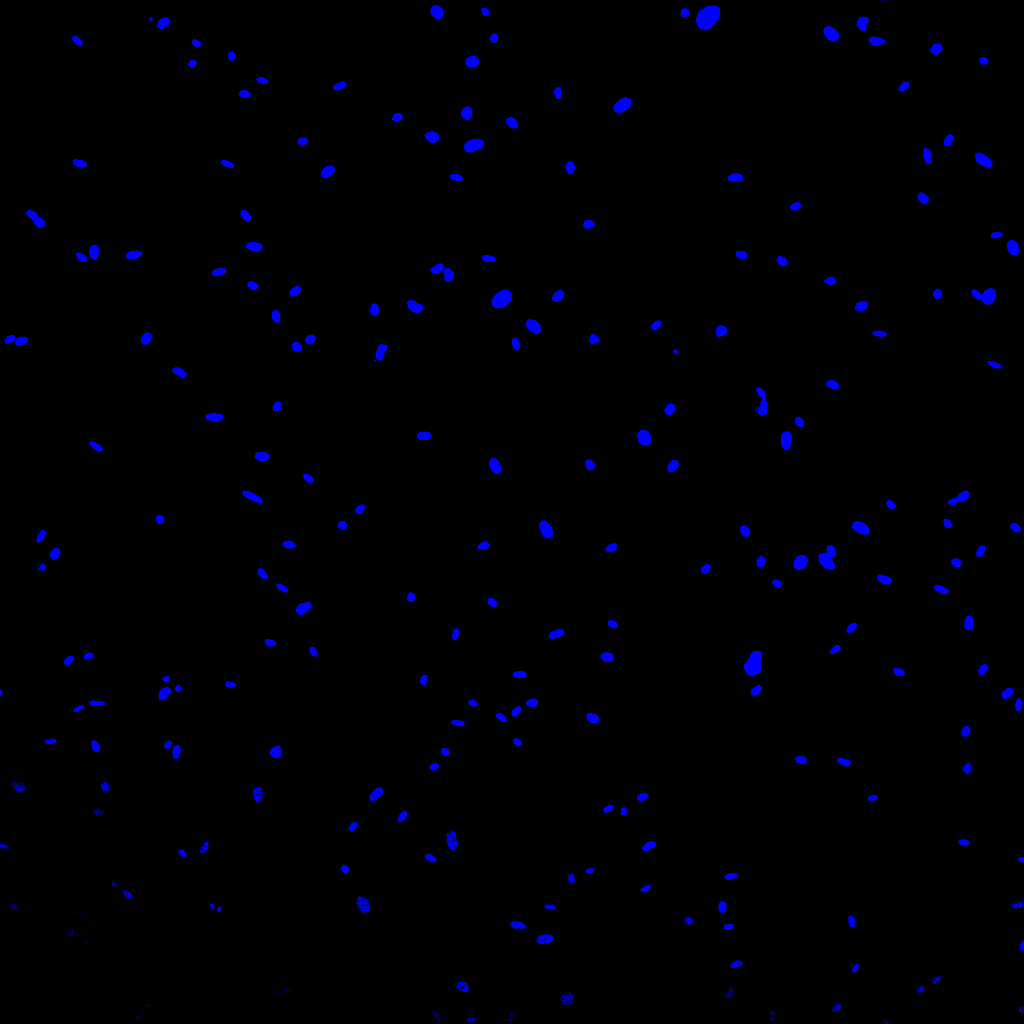

Supplement: Supplementary file 2 [file Presentation1.zip › EDU/U87-Sham/1 dapi.tif]

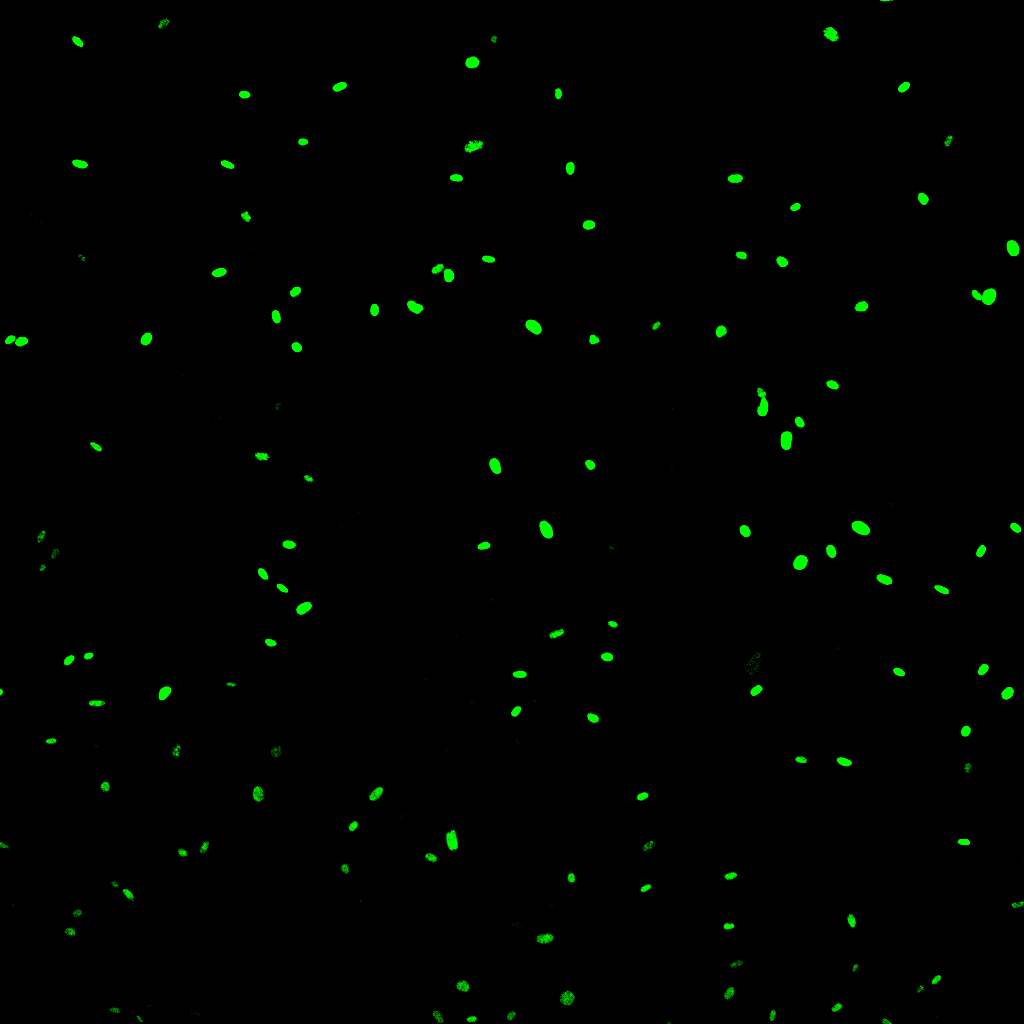

Supplement: Supplementary file 2 [file Presentation1.zip › EDU/U87-Sham/1 edu.tif]

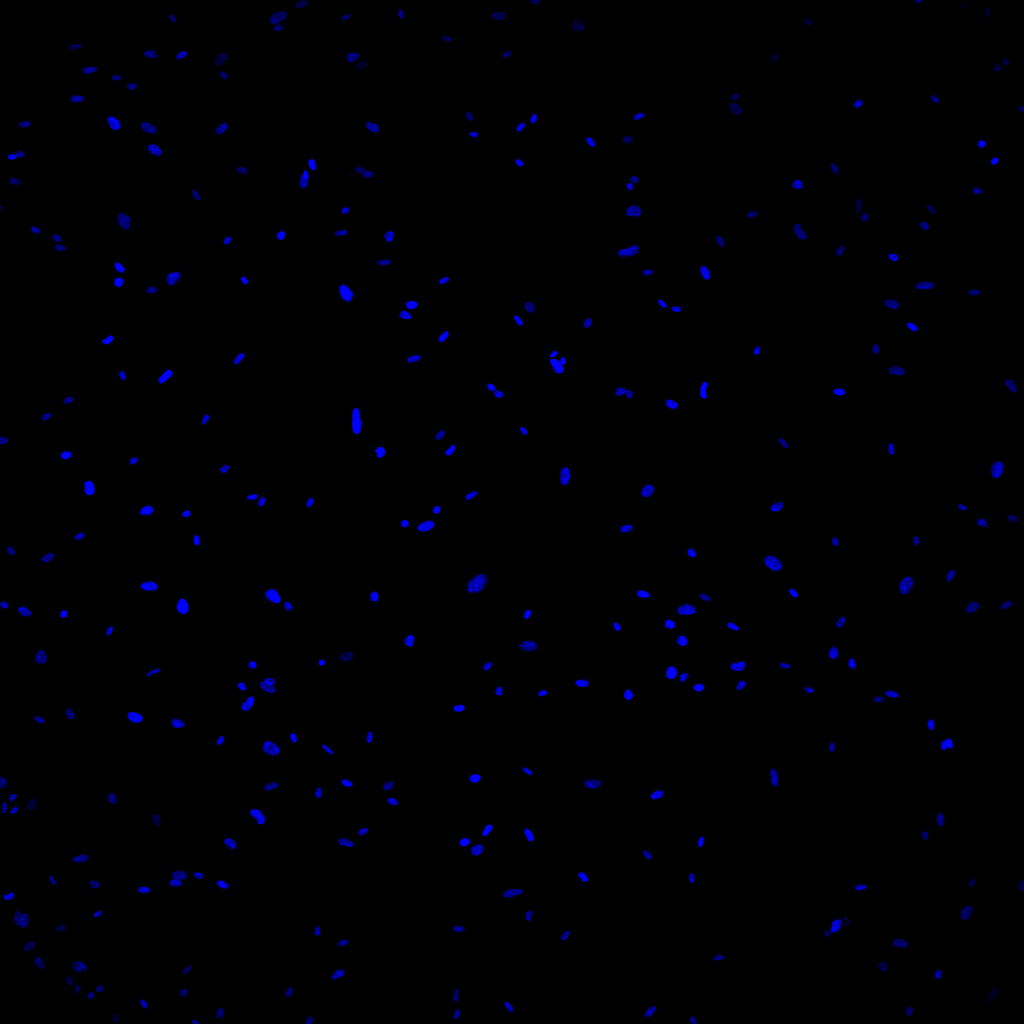

Supplement: Supplementary file 2 [file Presentation1.zip › EDU/U87-Sham/2 dapi.tif]

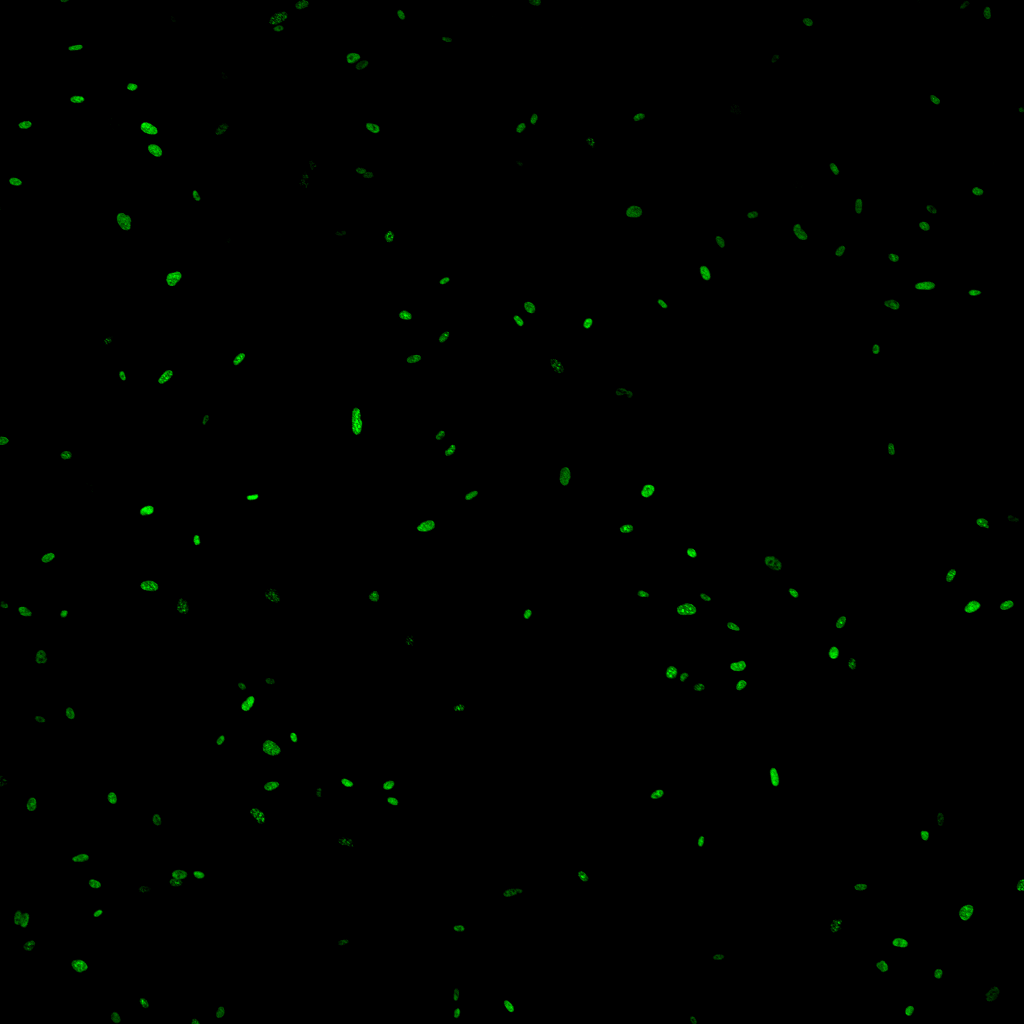

Supplement: Supplementary file 2 [file Presentation1.zip › EDU/U87-Sham/2 edu.tif]

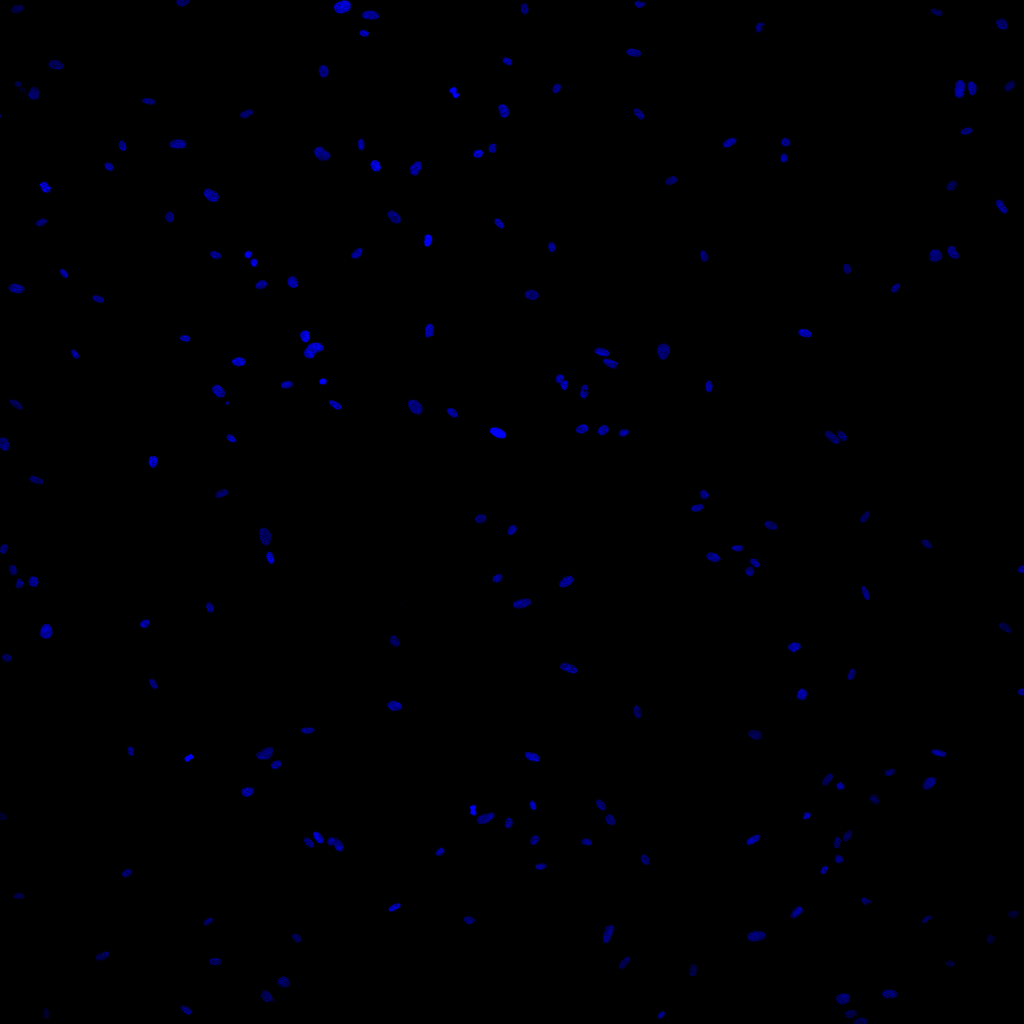

Supplement: Supplementary file 2 [file Presentation1.zip › EDU/U87-Sham/3 dapi.tif]

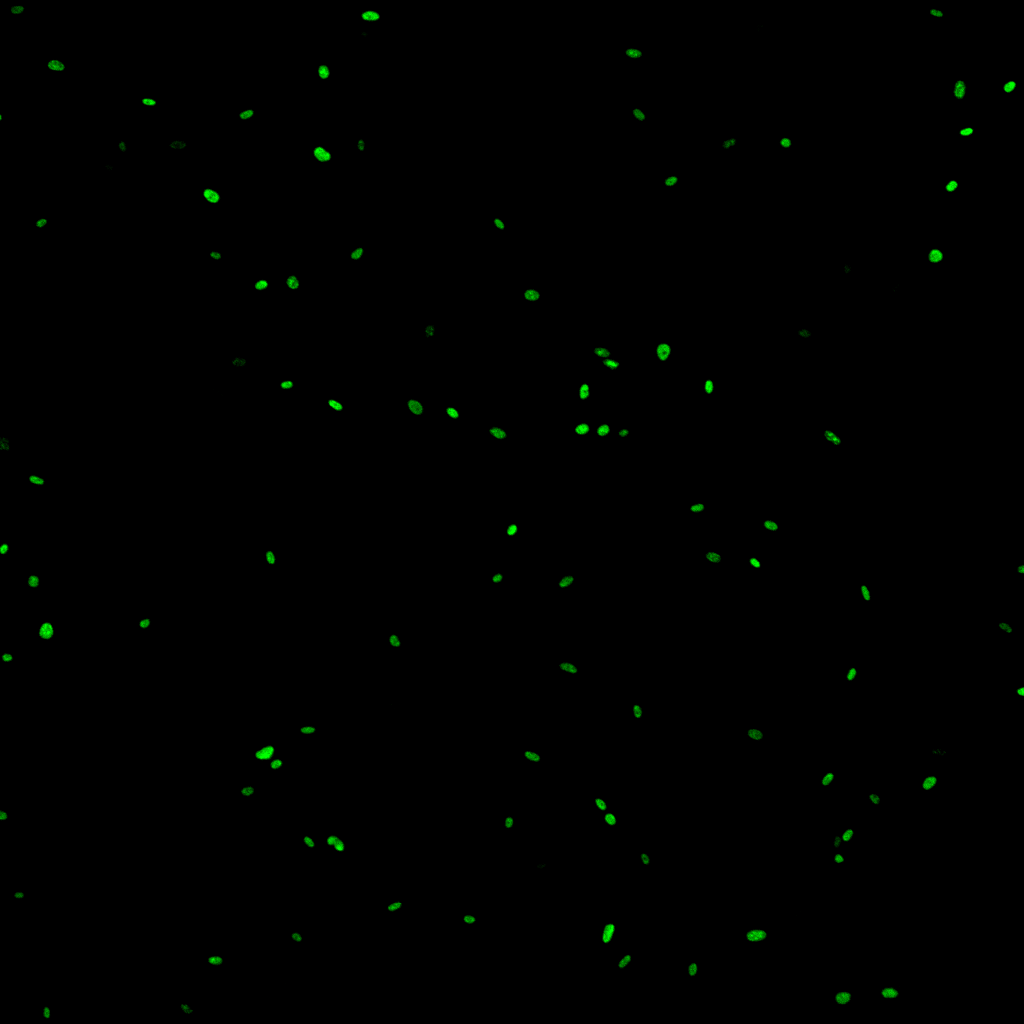

Supplement: Supplementary file 2 [file Presentation1.zip › EDU/U87-Sham/3 edu.tif]
